# Diffusion-rate sieving of propylene and propane mixtures in a cooperatively dynamic porous crystal

**DOI:** 10.1038/s41467-024-47268-7

**Published:** 2024-04-04

**Authors:** Yan Su, Ken-ichi Otake, Jia-Jia Zheng, Ping Wang, Qing Lin, Susumu Kitagawa, Cheng Gu

**Affiliations:** 1 State Key Laboratory of Luminescent Materials and Devices, Institute of Polymer Optoelectronic Materials and Devices, South China University of Technology, Guangzhou, 510640 P. R. China; 2 Institute for Integrated Cell-Material Sciences, Kyoto University, Yoshida, Sakyo-ku, Kyoto 606-8501 Japan; 3 Laboratory of Theoretical and Computational Nanoscience, National Center for Nanoscience and Technology, Chinese Academy of Sciences, Beijing, 100190 P. R. China; 4 College of Polymer Science and Engineering, State Key Laboratory of Polymer Materials Engineering, Sichuan University, Chengdu, 610065 P. R. China; 5 ReadCrystal Biotech Co., Ltd., Suzhou, 215505 P. R. China

**Keywords:** Metal-organic frameworks, Metal-organic frameworks, Organic molecules in materials science

## Abstract

Selective molecular recognition is an important alternative to the energy-intensive industrial separation process. Porous coordination polymers (PCPs) offer designing platforms for gas separation because they possess precise controllability over structures at the molecular level. However, PCPs-based gas separations are dominantly achieved using strong adsorptive sites for thermodynamic recognition or pore-aperture control for size sieving, which suffer from insufficient selectivity or sluggish kinetics. Developing PCPs that work at high temperatures and feature both high uptake capacity and selectivity is urgently required but remains challenging. Herein, we report diffusion-rate sieving of propylene/propane (C_3_H_6_/C_3_H_8_) at 300 K by constructing a PCP material whose global and local dynamics cooperatively govern the adsorption process via the mechanisms of the gate opening for C_3_H_6_ and the diffusion regulation for C_3_H_8_, respectively, yielding substantial differences in both uptake capacity and adsorption kinetics. Dynamic separation of an equimolar C_3_H_6_/C_3_H_8_ mixture reveals outstanding sieving performance with a C_3_H_6_ purity of 99.7% and a separation factor of 318.

## Abstract (web summary)

Porous coordination polymers (PCPs) are commonly used in gas separation processes but developing PCPs that work at high temperatures and feature both high uptake capacity and selectivity remains challenging. Here, the authors report diffusion-rate sieving of propylene/propane at 300 K by constructing a PCP whose global and local dynamics cooperatively govern the adsorption process via gate opening for propylene and diffusion regulation for propane.

## Introduction

**Fig. 1:**
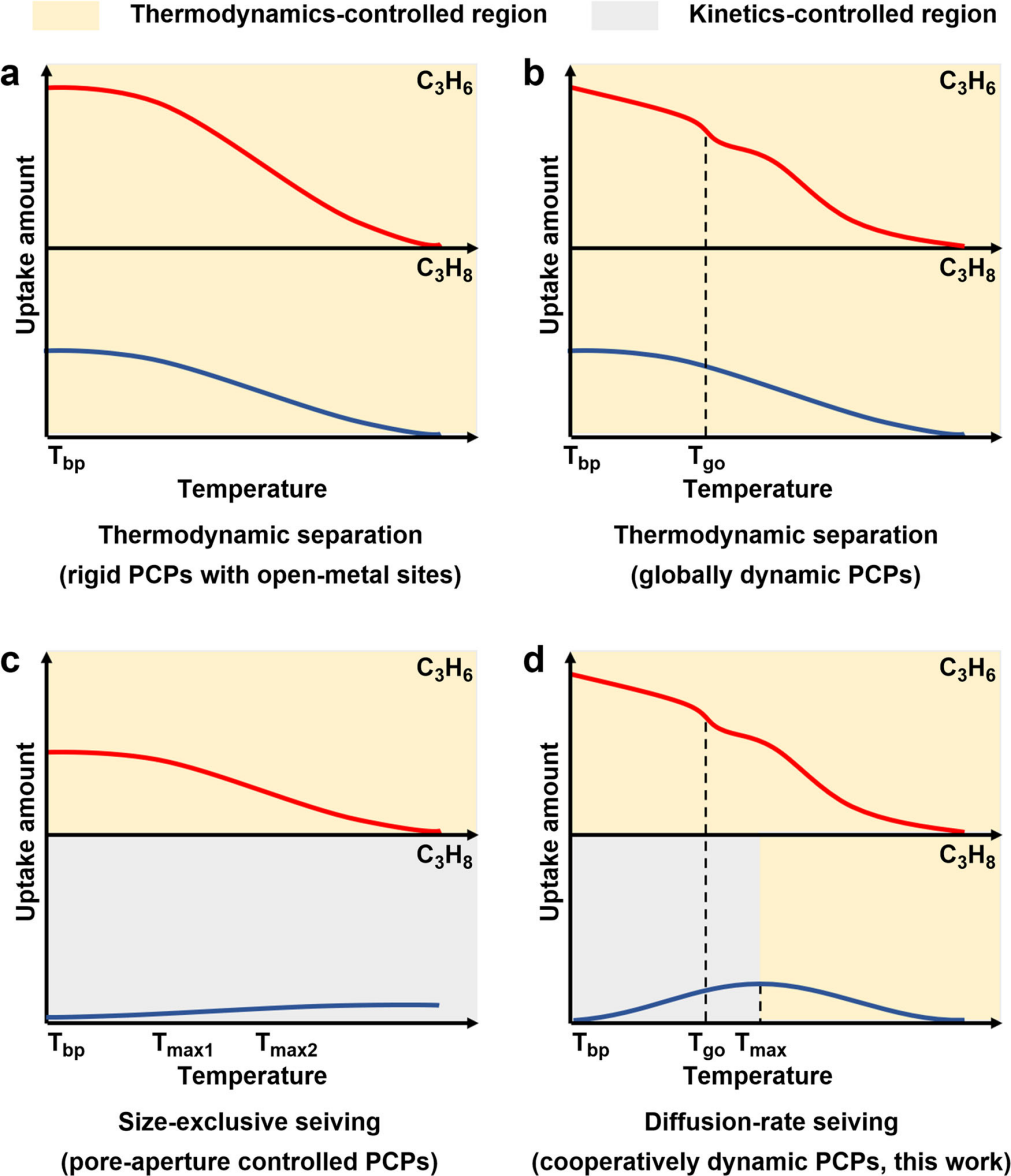
The Diffusion-rate sieving mechanism using a cooperatively dynamic PCP to separate C_3_H_6_ and C_3_H_8_. **a** Schematic representations of the C_3_H_6_/C_3_H_8_ adsorption isobars for the thermodynamic separation using rigid PCPs with open-metal sites. **b** Schematic representations of the C_3_H_6_/C_3_H_8_ adsorption isobars for the thermodynamic separation using globally dynamic PCPs. **c** Schematic representations of the C_3_H_6_/C_3_H_8_ adsorption isobars for the size-exclusive sieving using pore-aperture controlled PCPs. **d** Schematic representations of the C_3_H_6_/C_3_H_8_ adsorption isobars for the diffusion-rate sieving using a cooperatively dynamic PCP (this work); this mechanism involves a pore system featuring both gate-opening and diffusion-regulatory functionalities that can individually control the adsorption processes of different gases, thereby yielding substantial differences in both adsorption capacity and kinetics.

Propylene (C_3_H_6_) is an important petrochemical feedstock to manufacture polypropylene that requires purity of propylene higher than 99.5%^1^. Industrial separation of propylene with propane (C_3_H_8_) is dominantly accomplished by energy-intensive cryogenic distillation^2^, which motivates chemists to develop porous materials for adsorptive C_3_H_6_/C_3_H_8_ separation that is less energy-demanding. Porous coordination polymers (PCPs, or metal-organic frameworks) are attractive candidates as they provide precise controllability over the structures during adsorption and separation^3,4^. PCPs-based C_3_H_6_/C_3_H_8_ separation has been extensively studied and various separation mechanisms have been developed, such as isoreticular principle^5^, pore space partition^6^, open-metal sites (OMSs)^7–9^, surface engineering^10^, ligand modification^11^, molecular docking^12,13^, pore distortion^14^, inverse separation^15^, and size exclusion^16–18^. The essence of these approaches included the control of thermodynamics (Fig. 1a, b) and kinetics (Fig. 1c), with the size-exclusive sieving being an extreme scenario of the kinetically controlled process. Nevertheless, most of these approaches suffer from low to moderate separation factors or sluggish kinetics, even though rare reports have achieved both high selectivity and fast kinetics^19^. This is due to the similarity of C_3_H_6_ and C_3_H_8_ in physical properties and molecular size/shape (Supplementary Table 1), resulting in difficulty in designing pore systems with suitable environments and fine-tuned apertures with sub-nanometre precision.

Dynamic molecular sieving PCPs are expected to be advantageous if the targeted molecules could induce gate-opening at certain pressures^20^. However, the separation mechanism of such globally dynamic PCPs is still based on thermodynamics, with low-pressure gate-opening thresholds at low temperatures, which substantially move to higher pressures at high temperatures^21^, thus rendering many flexible PCPs exhibiting separation performance by gate-opening at low temperatures but losing this ability at high temperatures. By contrast, locally dynamic PCPs feature rigid framework structures with locally movable substituents that respond to external stimuli such as temperature^22,23^. By designing the motive substituents at the pore apertures with various motion mechanisms, such as chemical-triggered adaptative pore^24^, temperature-responsive pore apertures^25,26^, and orthogonal-array pore system^19^, locally dynamic PCPs have accomplished challenging gas separation. However, these PCPs encounter sluggish kinetics in the separation process because the thermodynamic equilibrium is difficult to achieve (Fig. 1c). Ideally, porous materials combining the advantages of global and local dynamic motions would offer a new type of separation, in which one gas triggers the gate-opening of the framework while its diffusion among the pores is unimpeded, whereas the other gas is not able to open the gate while its diffusion rate is lowered and regulated by the local dynamics of the framework. Thus far, such cooperative PCPs by means of combined global and local dynamic motions for efficient gas separation have yet to be demonstrated and accomplished.

Herein, we report efficient C_3_H_6_/C_3_H_8_ separation at 300 K by a diffusion-rate sieving mechanism, in which C_3_H_6_ and C_3_H_8_ in a PCP material exhibit over 60-fold differences in diffusion rates and thus ensuring the PCP to preferentially adsorb C_3_H_6_ and almost fully exclude C_3_H_8_. The essence of our mechanism is to construct a cooperative PCP, in which the global and local dynamics synergistically control the adsorption processes. C_3_H_6_ exhibits high gas-framework interaction energy and causes the PCP to globally deform accompanied by a gate-opening adsorption behavior, whereas the diffusion of C_3_H_8_ is regulated by the local motion of the ligand because the weak C_3_H_8_-PCP interaction renders the global structural change of PCP impossible (Fig. 1d). Consequently, the adsorptions of C_3_H_6_ and C_3_H_8_ at 300 K are individually governed by thermodynamics and kinetics, yielding remarkable C_3_H_6_/C_3_H_8_ selectivity, diffusion-rate difference, and C_3_H_6_ uptake amount, which make this separation mechanism distinct from the conventional size sieving with low C_3_H_6_ uptake and torpid kinetics. Diffusion-rate sieving from an equimolar C_3_H_6_/C_3_H_8_ mixture yields a separation factor of 318, C_3_H_6_ purity up to 99.7%, and C_3_H_6_ productivity of 19.5 L kg^–1^.

## Results

### PCP synthesis and structural analysis

**Fig. 2:**
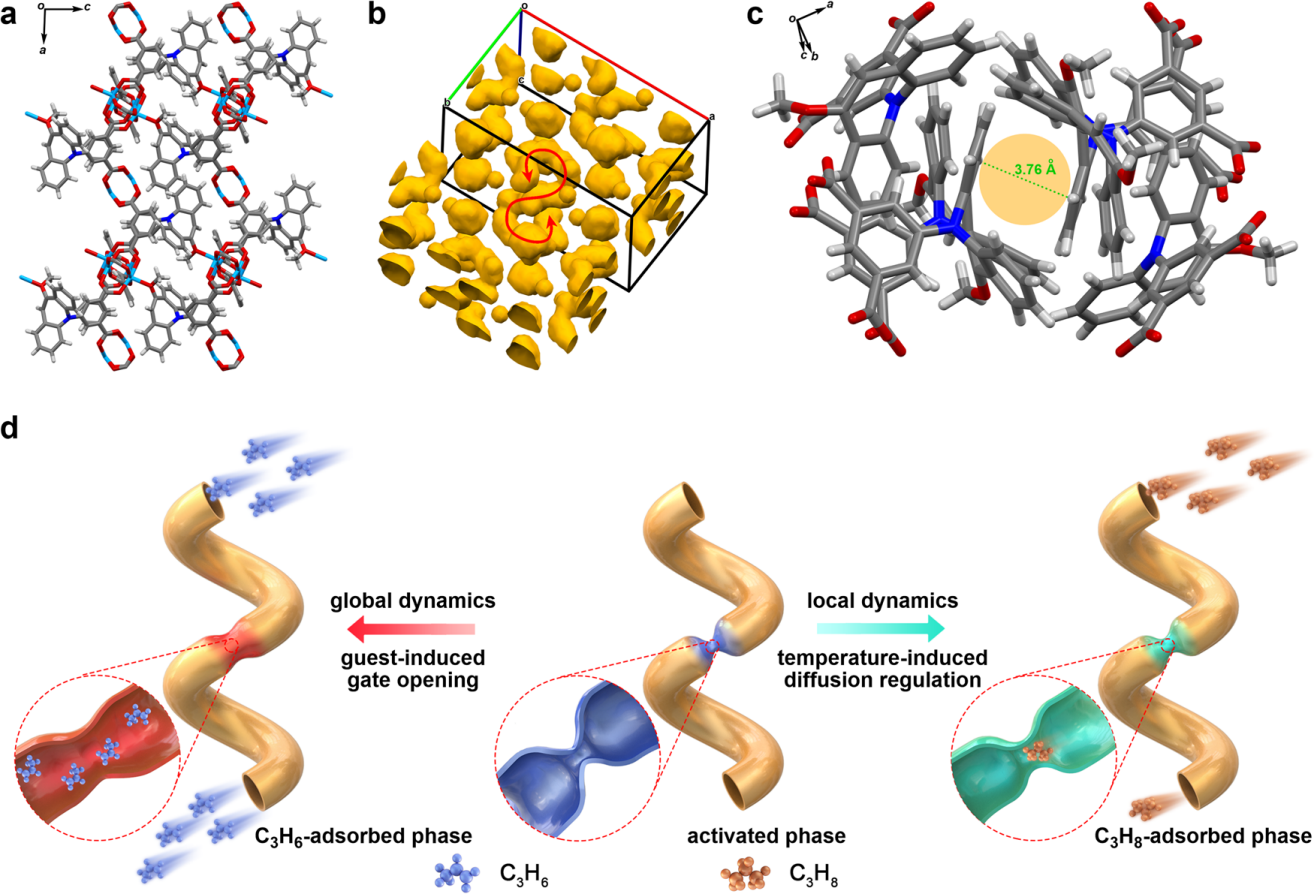
Depiction of the PCP structure. **a** The crystal structure of **FDC–4a** viewed along the *b* axis. Carbon, gray; nitrogen, blue; hydrogen, white; oxygen, red; copper, light blue. For clarity, the MODBAP moieties whose O atoms are uncoordinated with the OMSs of the Cu^2+^ paddle wheels are omitted. **b** The void in **FDC–4a** visualized by a small probe radius of 1.2 Å. The void volume is 1594 Å^3^ and corresponds to 12.3% of the unit-cell volume. The inner and outer surfaces of the pore are drawn in brown and yellow, respectively. The red arrow shows the helical pore. **c** The structure of the diffusion gate, which is surrounded by two H atoms on benzene rings of the two MODBAP moieties with a 3.76 Å distance. **d** Schematic diagram of the cooperatively dynamic PCP in this work. The PCP with one-dimensional (1D) transport pathways is constructed. The apertures are colored blue and red for the closed and open phases, respectively. In the closed phase, the pore entrances are smaller than the kinetic diameters of C_3_H_8_; hence, the pores are almost isolated, which regulates the diffusion of C_3_H_8_ by the thermal flipping of the gate moieties that slightly enlarge the gate (the apertures are colored green). In the open phase, the pore entrances become much larger because of the gate opening, and the pores open for C_3_H_6_ adsorption.

We designed a Cu-based PCP with an asymmetric, rhinoceros beetle-shape ligand comprising isophthalic acid and 5-(10-methoxy-5H-dibenzo[b,f]azepin-5-yl) (MODBAP) moieties (MODBAP-ipa; see Supplementary information and Supplementary Figs. 1–9), with the latter moiety exhibiting effective flip-flop local motion with flipping energy of 34.8 kJ mol^–1^ (Supplementary Fig. 10). The as-synthesized PCP, namely, Cu(MODBAP) (termed **FDC–4**, FDC = flip-flop dynamic crystal) (Supplementary Figs. 11, 12, Supplementary Table 2, Supplementary Data 1), possessed a Kagomé-type layered structure with the neighboring layers stacking in a staggered mode; one hexagonal pore was horizontally surrounded by the framework linked by Cu^2+^ paddle wheels and isophthalic groups, whereas six MODBAP moieties asymmetrically covered the pores from two vertical sides (Supplementary Fig. 13). Subsequently, **FDC–4** was subjected to solvent exchange and vacuum heating at 393 K to afford its activated phase (**FDC–4a**, Supplementary Figs. 14–18). The single-crystal structure of **FDC–4a** was determined by the continuous rotation electron diffraction (cRED) technique (Supplementary Figs. 16, 17, Supplementary Table 3). Activation gave rise to one-third of the OMSs on the Cu^2+^ paddle wheels coordinating with the O atoms on MODBAP moieties in the neighboring layers (Fig. 2a), thereby transforming **FDC–4a** into a robust three-dimensional framework with one-dimensional helical pores (Fig. 2b), which possessed only one type of small diffusion gate, surrounded by two H atoms on benzene rings of the two MODBAP moieties with 3.76 Å distance (Fig. 2c). Therefore, the thermal flipping of MODBAP units was expected to enlarge the gate size to promote the gas diffusion at high temperatures and shrink the gate to block the gas diffusion at low temperatures (Fig. 2d).

### Gas sorption and in-situ PXRD

**Fig. 3:**
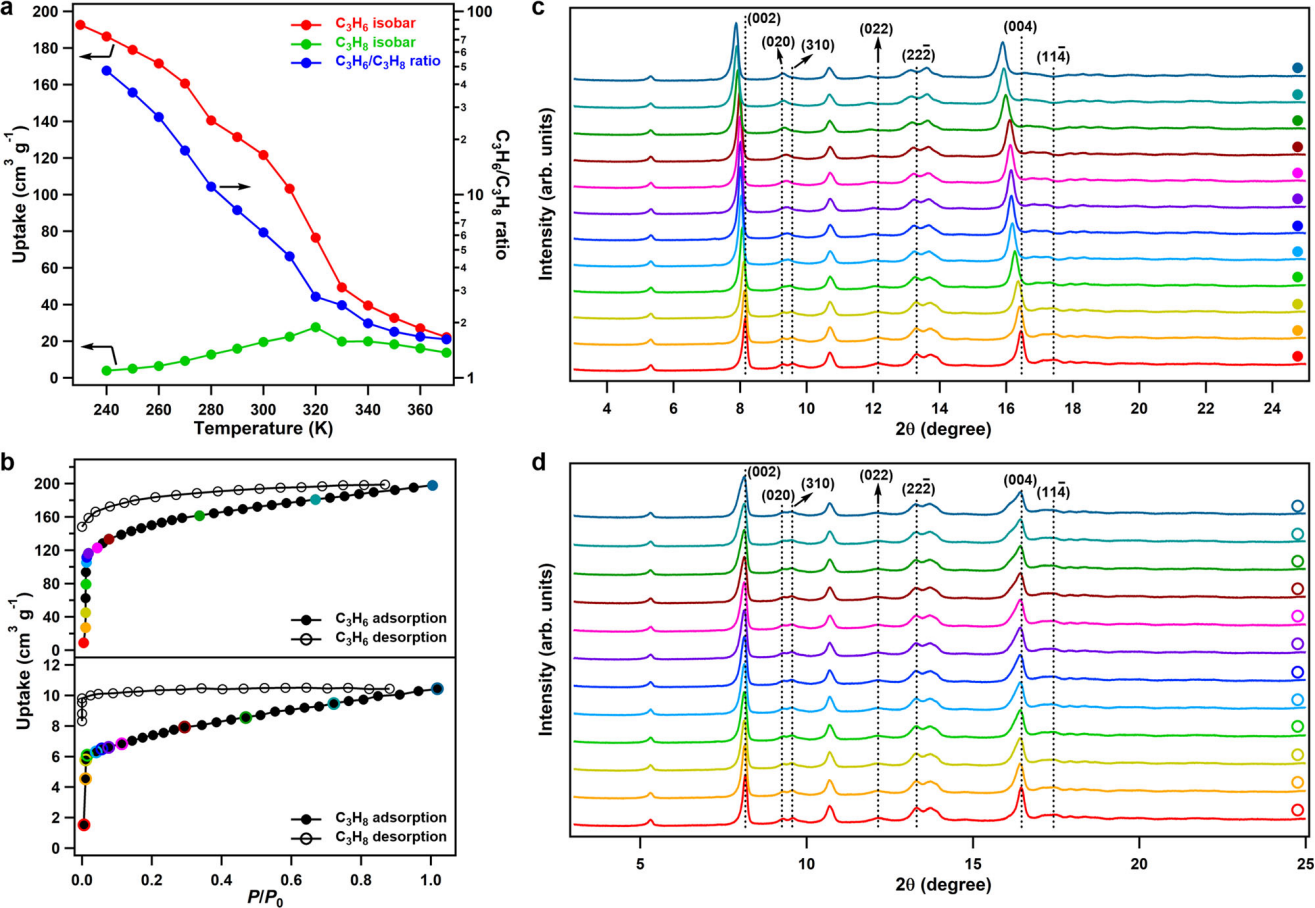
Gas adsorption and in-situ PXRD. **a** C_3_H_6_ and C_3_H_8_ adsorption isobars at 1 bar and the C_3_H_6_/C_3_H_8_ uptake ratio. **b** C_3_H_6_ and C_3_H_8_ sorption isotherms at 240 K. **c** Coincident in-situ adsorption/PXRD patterns during C_3_H_6_ adsorption measured at 240 K at given equilibrium pressures. **d** Coincident in-situ adsorption/PXRD patterns during C_3_H_8_ adsorption measured at 240 K at given equilibrium pressures.

**FDC–4a** exhibited distinct adsorption behavior depending on the sizes of the investigated gases, as revealed by their adsorption isobars (Supplementary Fig. 19)^27,28^. In the cases of CO_2_ and C_2_H_2_ whose kinetic diameters (3.30 Å) were smaller than the diffusion gate of **FDC–4a**, the adsorption amounts of the two gases monotonously decreased with increasing temperature from 200 to 370 K, indicating a thermodynamics-controlled process. By contrast, when the gases possessed kinetic diameters similar to (N_2_ and CO, 3.64–3.80 Å) or larger than (C_2_H_4_ and C_2_H_6_, 4.16–4.44 Å) the diffusion gate, they showed volcano-type isobars that demonstrated domination of kinetics and thermodynamics at low (80 to 150 K for N_2_/CO, 180 to 210 K for C_2_H_4_, and 190 to 270 K for C_2_H_6_) and high (160 to 370 K for N_2_/CO, 220 to 370 K for C_2_H_4_, and 200 to 370 K for C_2_H_6_) temperatures^25^, respectively. Remarkably, monotonously decreased and volcano-type isobars were observed for C_3_H_6_ and C_3_H_8_, respectively (Fig. 3a), evidencing that their adsorptions were governed by thermodynamical and diffusion-regulatory factors, respectively. Isotherm measurements at individual temperatures revealed the same trends with isobars (Supplementary Fig. 20). Therefore, the different adsorption behaviors of C_3_H_6_ and C_3_H_8_ suggested a high C_3_H_6_/C_3_H_8_ selectivity (Fig. 3a, Supplementary Table 4). More importantly, **FDC–4a** maintained a high C_3_H_6_ uptake of 122 cm^3^ g^–1^ at 300 K and such a balance between uptake and selectivity catapulted **FDC–4a** to the region of ideal sieving^18^. Additionally, the isosteric heat (*Q*_st_) value of C_3_H_6_ adsorption was calculated to be 35.0 kJ mol^–1^ (Supplementary Fig. 21), which was lower than many of the C_3_H_6_-selective materials^4–19^ (Supplementary Table 4) and thus indicating an easy regeneration in separation.

To unveil the structural change of **FDC–4a** during C_3_H_6_ and C_3_H_8_ adsorption, coincident in-situ powder X-ray diffraction (PXRD) patterns were recorded^29^. The C_3_H_6_-sorption isotherm at 240 K revealed a steep increase in the uptake amount at the low-pressure range (Fig. 3b) accompanied by the gate-opening behavior, as shown in the in-situ PXRD (Fig. 3c). The peaks attributed to the (002), (022), (22-2), (004), and (11-4) facets continuously shifted to lower angles, indicative of the increased distances of these facets, whereas the peaks belonging to the (020) and (310) facets merged into a new peak. These results indicated that the gate opening gradually and smoothly occurred, rather than the abrupt or stepped gate opening in many soft PCPs. We further collected the coincident in-situ isobar/PXRD patterns for C_3_H_6_ adsorption in the temperature range of 230 to 370 K (Supplementary Figs. 22, 23), showed temperature-responsive gating-opening behavior due to the thermodynamics-controlled change of gas-framework affinity. By contrast, C_3_H_8_ hardly caused the structural change of **FDC–4a**, as confirmed by both isotherm and isobar measurements (Fig. 3, b, d, Supplementary Figs. 24, 25). Therefore, its diffusion in **FDC–4a** was regulated by the pore apertures and exhibited volcano-type adsorption isobar due to the competition of thermodynamics and kinetics.

### Adsorption kinetics

**Fig. 4:**
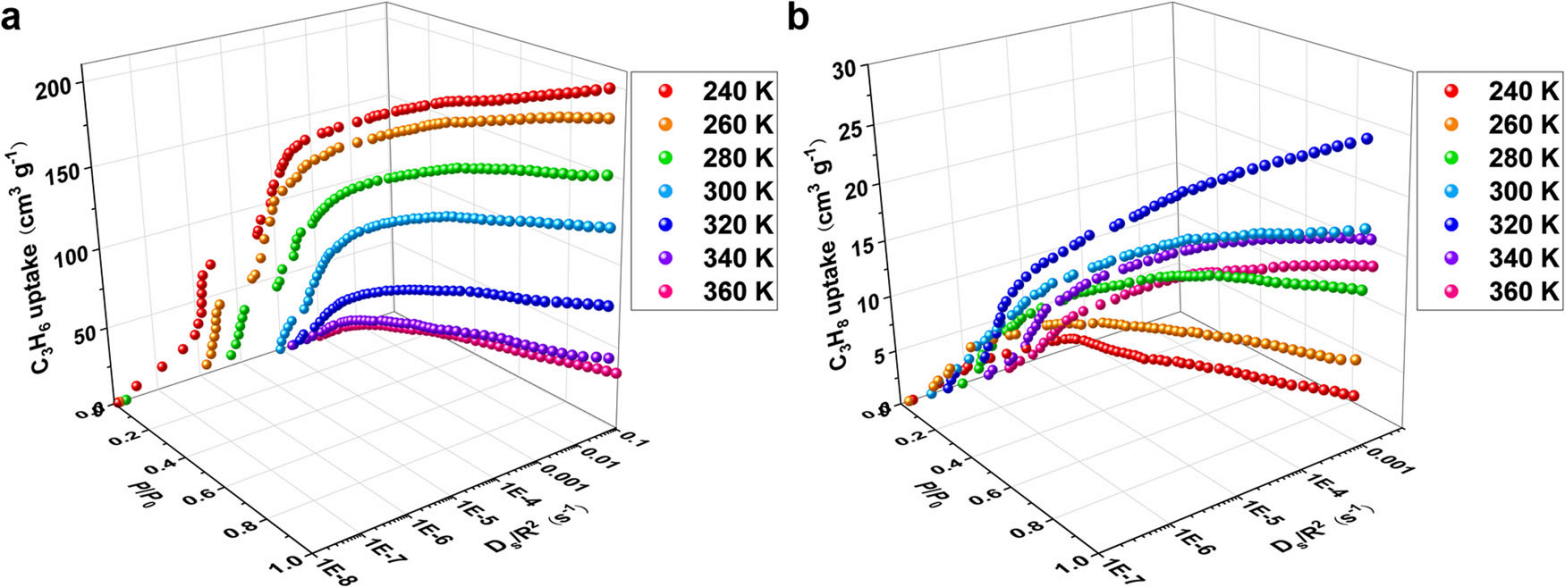
Diffusion rates of C_3_H_6_ and C_3_H_8_ in FDC–4a. **a** Global pressure–diffusion-rate–adsorption-amount (*P*–*D*_s_/*R*^2^–*V*) landscape for **FDC–4a** adsorbing C_3_H_6_, where R denotes the radius of a PCP particle. **b** *P*–*D*_s_/*R*^2^–*V* landscape for **FDC–4a** adsorbing C_3_H_8_.

To evaluate the adsorption kinetics for C_3_H_6_ and C_3_H_8_, we quantified the diffusion rate by Crank theory for every C_3_H_6_ or C_3_H_8_ adsorption plot in the 240 to 360 K range (supplementary information). This allowed us to produce the global *P*–*D*_s_/*R*^2^–*V* and *T*–*D*_s_/*R*^2^–*V* landscapes, where *P* (relative pressure), *T* (K), *V* (cm^3^ g^–1^), and *D*_s_ (R^2^ s^–1^) denote the pressure, temperature, uptake volume, and diffusion rate, respectively, where R represents the radius of a PCP particle (Fig. 4a, b, Supplementary Fig. 26). Both C_3_H_6_ and C_3_H_8_ exhibited increased diffusion rates along with pressure and temperature. At the beginning of the C_3_H_6_ adsorption at 240, 260, and 280 K, the C_3_H_6_ diffusion was largely hindered with substantially low *D*_s_ values of less than 10^–5^ R^2^ s^–1^. When the pressure was increased to reach the gate opening, the C_3_H_6_ diffusion rates were abruptly increased by about 10 folds. Afterward, the C_3_H_6_ diffusion rates markedly increased with pressure. At the relative pressure close to 1.0, the C_3_H_6_ diffusion rates were 5.05 × 10^–2^ and 7.09 × 10^–2^ R^2^ s^–1^ at 240 and 300 K, respectively. By contrast, the C_3_H_8_ diffusion rates smoothly increased with pressure without abrupt increment observed, and the *D*_s_ values at the relative pressure close to 1.0 were 8.73 × 10^–4^ and 1.13 × 10^–3^ R^2^ s^–1^ at 240 and 300 K, respectively. C_3_H_6_ exhibited diffusion rates of 57.8- and 62.7-folds of those of C_3_H_8_ at 240 and 300 K, respectively, indicating that the substantial differences could promote effective diffusion-rate sieving of C_3_H_6_/C_3_H_8_.

### VT-PXRD and computational studies

To understand the adsorption behavior from a structural perspective, synchrotron variable-temperature PXRD (VT-PXRD) patterns for **FDC–4a** were recorded, which revealed inconspicuous peak shifts to lower angles with increasing temperature under vacuum (Supplementary Figs. 27, 28). Taking the peak attributed to the (002) plane as an example, the peak shifted by 0.042° from 90 to 375 K. This slight structural change was apparently different from the global dynamics of **FDC–4a** showing obvious PXRD shifts, thereby demonstrating a temperature-responsive local dynamics in **FDC–4a**. The peak shift indicated the expansion of the [002] axis. Because there were two MODBAP moieties lying on the (002) plane, this small expansion could be induced by the thermal flipping of MODBAP moieties, which enlarged the diffusion gates to facilitate adsorption (Supplementary Fig. 29).

Density functional theory calculations were carried out to understand the difference between C_3_H_6_ and C_3_H_8_ adsorption and diffusion in **FDC–4a**. Because there was little change in lattices of **FDC–4a** during a low-amount C_3_H_6_ adsorption and overall C_3_H_8_ adsorption, we only considered adsorptions of these two gases in the activated phase of **FDC–4a** to elucidate the reason for the high selectivity of **FDC–4a** towards C_3_H_6_/C_3_H_8_ separation. Two different adsorption sites were found for both C_3_H_6_ and C_3_H_8_ in **FDC–4a** (Supplementary Fig. 30, see supplementary methods for computational details). The binding energies (BEs) of gas molecules with **FDC–4a** at these adsorption sites were calculated as ‒47.3 (site I) and ‒34.9 kJ mol^‒1^ (site II) for C_3_H_6_ and ‒48.7 (site I) and ‒23.2 kJ mol^‒1^ (site II) for C_3_H_8_ (Supplementary Table 5). The more negative BE of C_3_H_6_ at site II indicated its stronger affinity to **FDC–4a** than C_3_H_8_, consistent with the larger adsorption amount of C_3_H_6_ than that of C_3_H_8_ at high temperatures such as 360 K. On the contrary, the similar BE values of C_3_H_6_ and C_3_H_8_ at the site I suggested that the adsorption amount of C_3_H_8_ should be comparable to that of C_3_H_6_ at very low loadings (<20 mL g^‒1^), which was seemingly against the experimental observation. This is because the kinetic factor plays an important role in the selective adsorption of C_3_H_6_ over C_3_H_8_, as discussed above; the diffusion rate of C_3_H_8_ is so low that the C_3_H_8_ transport in **FDC–4a** is significantly hindered, thereby its adsorption amount is much lower than that in the equilibrium state. To further clarify this point, we calculated the diffusion barriers of C_3_H_6_ and C_3_H_8_ in **FDC–4a** (Supplementary Fig. 31). As expected, the calculated diffusion barrier of C_3_H_8_ was as high as 77.3 kJ mol^‒1^, which impeded the transport of C_3_H_8_ even near room temperature. On the other hand, the diffusion barrier for C_3_H_6_ was approximately 60.0 kJ mol^‒1^, which was much smaller than that of C_3_H_8_ diffusion, suggesting that C_3_H_6_ could enter the pores of **FDC–4a** more easily than C_3_H_8_. These results indicate that the adsorption and transport of C_3_H_6_ into **FDC–4a** are more favorable than those of C_3_H_8_ from the viewpoints of adsorption thermodynamics and kinetics, essentially promoting the C_3_H_6_/C_3_H_8_ separation performance.

### Mixed gas separation

**Fig. 5:**
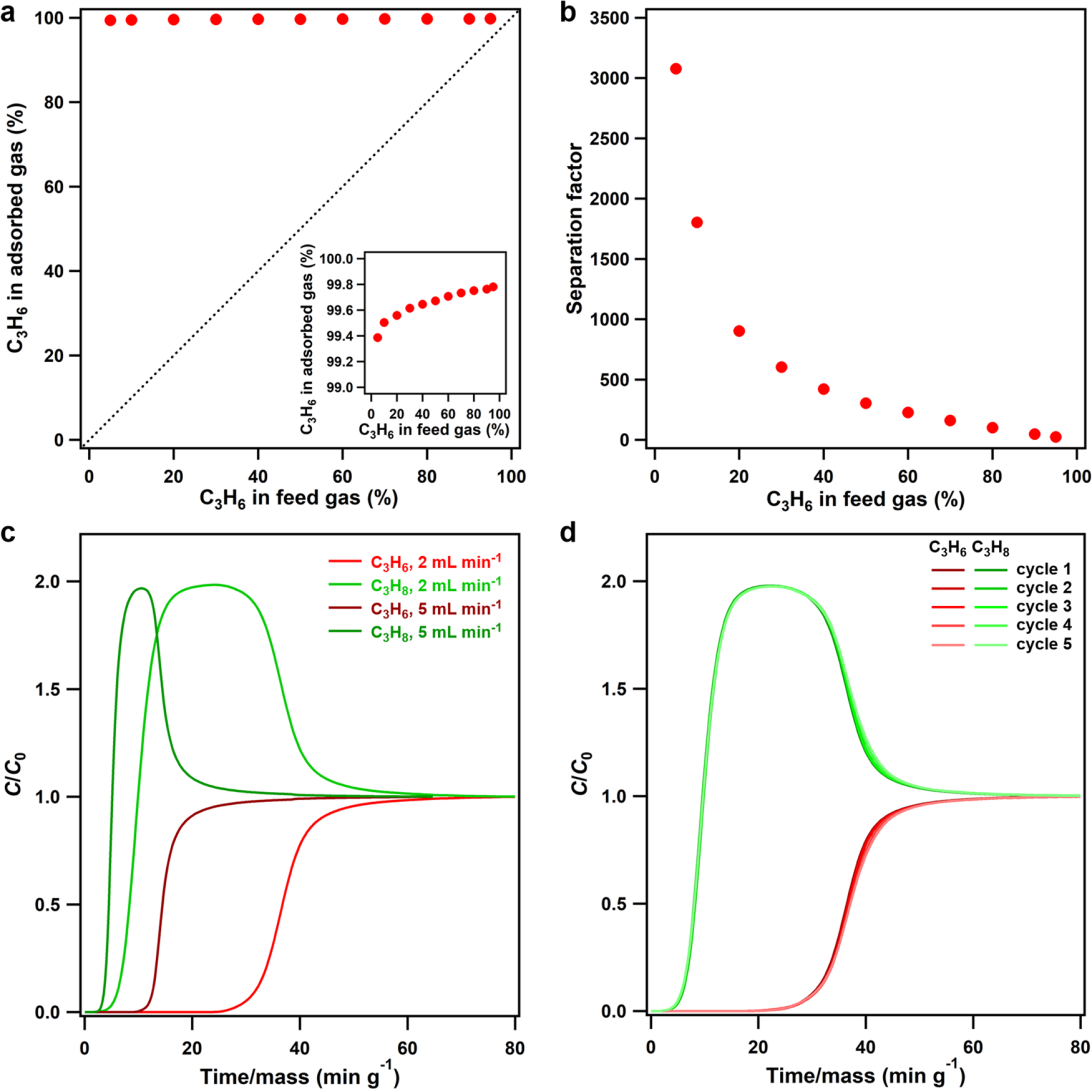
Mixed gas separation. **a** McCabe–Thiele diagram for C_3_H_6_/C_3_H_8_ separation by **FDC–4a** at 300 K, with the dashed line representing the theoretical behavior of showing no selectivity. The inset is the enlarged Y-axis showing the C_3_H_6_ fractions in the adsorbed phase. **b** The correlation between C_3_H_6_ concentration in the feed gas and the separation factor for **FDC–4a**. **c** The breakthrough curve of an equimolar C_3_H_6_/C_3_H_8_ mixture (total flow rate of 4.0 and 10.0 mL min^−1^, respectively) on **FDC–4a** at 300 K. *C* and *C*_0_ are the concentrations of each gas at the outlet and inlet, respectively. **d** The breakthrough curves for a cycling test of an equimolar C_3_H_6_/C_3_H_8_ mixture (total flow rate of 4.0 mL min^−1^) on **FDC–4a** at 300 K.

The sorption mechanism inspired us to perform dynamic separation of the C_3_H_6_/C_3_H_8_ mixtures by a temperature-programmed desorption (TPD) protocol (Supplementary Figs. 32, 33); the experiments were carried out at 300 K with an equimolar C_3_H_6_/C_3_H_8_ mixture. **FDC–4a** preferentially adsorbed C_3_H_6_ from the C_3_H_6_/C_3_H_8_ mixture within a short exposure time of 1 h, resulting in a remarkable C_3_H_6_ enrichment with a concentration as high as 99.7% in the adsorbed phase (Fig. 5a, Supplementary Fig. 34) and a separation factor of 318 (Fig. 5b). The productivity of C_3_H_6_ with 99.7% purity in a single adsorption-desorption cycle, estimated from the C_3_H_6_ TPD spectra and the calibration curve, was 19.5 L kg^–1^, which was comparable to JNU-3a (34.2 L kg^–1^, 99.5% purity)^19^, KAUST-7 (16.3 L kg^–1^, 90.0% purity)^16^, and Y-abtc (1.3 L kg^–1^, 90.0% purity)^6^. These results suggested the potential of **FDC–4a** for isolating C_3_H_6_ from the industrial equimolar C_3_H_6_/C_3_H_8_ mixture. The separation factor decreased with prolonged exposure time (Supplementary Figs. 34–39), indicating that C_3_H_6_ was adsorbed substantially faster than C_3_H_8_ and occupied most of the available space, which excluded C_3_H_8_ by the diffusion-rate sieving mechanism as a consequence. Remarkably, **FDC–4a** exhibited high separation performance over a wide range of feed-gas compositions (Fig. 5a, Supplementary Figs. 40–49). Even when the mixture contained ultralow C_3_H_6_ of 5 mol%, the C_3_H_6_ concentration in the adsorbed phase was 99.4%, corresponding to an outstanding separation factor of 3078 (Fig. 5b), demonstrating that the diffusion-rate sieving is key to achieving exceptional selectivity.

Dynamic column breakthrough experiments indicate practical aspects of the separation capability of **FDC–4a**. Initially, an equimolar C_3_H_6_/C_3_H_8_ mixture was passed through the **FDC–4a** column with a flow rate of 4 mL min^–1^. The mixture was efficiently separated with the C_3_H_6_ purity and productivity obtained from the desorption curve of 99.1% and 19.5 L kg^–1^, respectively (Fig. 5c, Supplementary Fig. 50). **FDC–4a** retained good separation performance with the equimolar C_3_H_6_/C_3_H_8_ mixture even under a much higher flow rate of 10 mL min^–1^, with the C_3_H_6_ purity and productivity of 98.9% and 21.2 L kg^–1^, respectively (Fig. 5c, Supplementary Fig. 51), indicating the fast kinetics during separation. On the other hand, **FDC–4a** was adaptable for separating C_3_H_6_/C_3_H_8_ mixtures with varied compositions; the C_3_H_6_/C_3_H_8_ mixtures with molar ratios of 10:90 and 90:10 were also separated by **FDC–4a** (Supplementary Figs. 52–55). The C_3_H_6_ productivity under different desorption conditions was also investigated; under the desorption temperatures of 393 and 300 K, the C_3_H_6_ productivity was 19.5 (99.1% purity) and 19.2 (99.1% purity) L kg^–1^, respectively (Supplementary Fig. 56), demonstrating that the physisorption with low gas-framework affinity in **FDC–4a** was beneficial for energy-saving production of C_3_H_6_. Finally, **FDC–4a** exhibited stability under continuous 5 breakthrough cycles (Fig. 5d), suggesting its potential in real separation applications.

## Discussion

This work demonstrates the efficient diffusion-rate sieving of C_3_H_6_/C_3_H_8_ in a PCP material by manipulating the global and local dynamics of the framework. The TPD experiments reveal kinetics-based sieving separation of an equimolar C_3_H_6_/C_3_H_8_ mixture at 300 K with a separation factor of 318, C_3_H_6_ purity up to 99.7%, and C_3_H_6_ productivity of 19.5 L kg^–1^ in a single adsorption-desorption cycle. These striking separation performances are ascribed to the underlying mechanism, which is realized by the cooperation of global dynamics of the framework upon C_3_H_6_ adsorption and local dynamics of gate constituents to regulate C_3_H_8_ diffusion. This design rationale can be more broadly suitable for various porous materials for efficient gas separation.

## Methods

### Synthesis of MODBAP-ipa ligand

#### Synthesis of dimethyl 5-(10-methoxy-5*H*-dibenzo[*b,f*]azepin-5-yl)isophthalate (1)

Dimethyl 5-iodoisophthalate (19.20 g, 60.0 mmol, 1.2 eq.), 10-methoxy-5*H*-dibenzo[*b,f*]azepine (11.16 g, 50.0 mmol, 1.0 eq.), 2-dicyclohexylphosphino-2’,4’,6’-triisopropylbiphenyl (XPhos, 1.19 g, 2.5 mmol, 0.05 eq.), tris(dibenzylideneacetone)dipalladium(0) (1.37 g, 1.5 mmol, 0.03 eq.), cesium carbonate (32.58 g, 100.0 mmol, 2.5 eq.), and toluene (200 mL) were placed in a flask whose inner gas was replaced by N_2_. The mixture was stirred at 115 °C for 48 h. After cooling down to room temperature, the reaction mixture was filtered through Celite®. The filtrate was diluted with ethyl acetate (200 mL) and washed with water. The organic phase was dried over MgSO_4_, filtered, and evaporated under reduced pressure. The residue was purified by column chromatography (SiO_2_, ethyl acetate/*n*-hexane with the ratio changing from 3 to 6%) to give **1** (11.22 g, yield = 54%) as a light-yellow solid. ^1^H NMR (500 MHz, DMSO-*d*_6_): δ (ppm) = 7.80 (2H, d, *J* = 11.5 Hz, Ph C_2_-*H* and MODBAP C_6_-*H*), 7.70 (1H, t, *J* = 7.6 Hz, MODBAP C_9_-*H*), 7.60 (1H, d, *J* = 7.9 Hz, MODBAP C_8_-*H*), 7.55 (3H, dd, *J* = 15.5, 6.6 Hz, MODBAP C_1,4,7_-*H*), 7.47 (1H, t, *J* = 7.5 Hz, MODBAP C_2_-*H*), 7.42 (1H, t, *J* = 7.4 Hz, MODBAP C_3_-*H*), 7.04 (2H, s, Ph C_4,6_-*H*), 6.29 (1H, s, MODBAP C_11_-*H*), 3.75 (9H, s, -CO_2_C*H*_3_ and -OC*H*_3_); ^13^C NMR (126 MHz, DMSO-*d*_6_): δ (ppm) = 165.97, 155.83, 149.06, 135.73, 133.89, 131.97, 131.14, 130.93, 129.73, 129.40, 128.89, 128.61, 128.46, 128.20, 119.35, 115.67, 102.71, 55.94, 52.81; MALDI-TOF-MS: calcd. *m/z* = 415.1420; found *m/z* = 415.654.

#### Synthesis of 5-(10-methoxy-5*H*-dibenzo[*b,f*]azepin-5-yl)isophthalic acid (MODBAP-ipa)

To the THF/MeOH (200 mL, 1/1 v/v) solution containing **1** (10.0 g, 25.9 mmol) was added 2 M NaOH aqueous solution (200 mL, 400 mmol) and the system was reflexed for 16 h. After cooling to 0 °C, the reaction mixture was acidified with concentrated HCl. The precipitate was collected by filtration, washed with water, and then dried under reduced pressure at 60 °C to give **MODBAP-ipa** (8.0 g, yield = 90%) as a white solid. ^1^H NMR (500 MHz, DMSO-*d*_6_): δ (ppm) = 13.03 (2H, s, -CO_2_*H*), 7.83-7.75 (2H, m, Ph C_2_-*H* and MODBAP C_6_-*H*), 7.68 (1H, t, *J* = 7.6 Hz, MODBAP C_9_-*H*), 7.59 (1H, d, *J* = 7.9 Hz, MODBAP C_8_-*H*), 7.53 (3H, dt, *J* = 15.6, 7.8 Hz, MODBAP C_1,4,7_-*H*), 7.46 (1H, t, *J* = 7.4 Hz, MODBAP C_2_-*H*), 7.40 (1H, t, *J* = 7.4 Hz, MODBAP C_3_-*H*), 7.03 (2H, s, Ph C_4,6_-*H*), 6.29 (1H, s, MODBAP C_11_-*H*), 3.76 (3H, s, -OC*H*_3_); ^13^C NMR (126 MHz, DMSO-*d*_6_): δ = 167.21, 155.84, 148.88, 142.12, 140.43, 135.81, 133.99, 132.15, 131.92, 130.89, 129.89, 129.55, 128.84, 128.56, 128.32, 128.08, 115.76, 102.78, 55.94; MALDI-TOF-MS: calcd. *m/z* = 387.1107; found *m/z* = 387.603.

### Synthesis of FDC–4

Firstly, 50 mg (0.13 mmol) **MODBAP-ipa** was dissolved in 6 mL DMF at room temperature. An aqueous solution (4 mL) of Cu(NO_3_)_2_·3H_2_O (62.4 mg, 0.26 mmol) was added to the above solution. Then the mixture was heated at 80 °C for 24 h. **FDC–4** was obtained as green lamellar crystals with sizes up to several hundreds of micrometers (70 mg, yield = 65%). The crystals were filtered, washed with DMF (10 mL, 3 times) and H_2_O (10 mL, 3 times), and dried in air. The as-synthesized **FDC–4** was characterized by infrared spectra (Supplementary Fig. 12). The adsorption peak of the stretching vibration of the C = O double bond shifted to a low wavenumber, indicative of the coordination bond formation in **FDC–4**.

### Solvent exchange and activation of FDC–4

To measure the adsorption property of **FDC–4**, we exchanged the guest and coordination solvents (DMF) with methanol by soaking **FDC–4** in methanol at 60 °C for 7 days. Every 24 h the methanol was replaced by a new one. After the solvent exchange, the exchanged **FDC–4** was dried under vacuum at 60 °C for 3 h. ^1^H NMR confirmed that the DMF in the exchanged **FDC–4** was exchanged by methanol (Supplementary Fig. 14). TG curve showed that the framework of the exchanged **FDC–4** was thermally stable until 170 °C (Supplementary Fig. 15). Thus, we activated the exchanged **FDC–4** at 120 °C for 11 h to afford **FDC–4a**; this temperature ensured the complete removal of the solvents meanwhile excluding the possibility of framework decomposition.

### Supplementary information

Supplementary Information

Peer Review Filef

### Source data

Source Data

## Data Availability

Source data are provided with the study. All other information can be obtained from the corresponding author upon request. The X-ray crystallographic coordinates for structures reported in this study have been deposited at the Cambridge Crystallographic Data Centre (CCDC), under deposition numbers 2236283-2236284. These data can be obtained free of charge from The Cambridge Crystallographic Data Centre via www.ccdc.cam.ac.uk/data_request/cif. Source data are provided with this paper.
