# Supplementary material for: Diffusion-rate sieving of propylene and propane mixtures in a cooperatively dynamic porous crystal
Source: Nat Commun. 2024 Apr 4;15:2898. doi: 10.1038/s41467-024-47268-7 (PMC10995200; doi:10.1038/s41467-024-47268-7)
Supplement: Supplementary file 1 — Supplementary Information [file 41467_2024_47268_MOESM1_ESM.pdf]

Supplementary information

Diffusion-rate sieving of propylene and propane mixtures in a cooperatively dynamic porous crystal

Yan Su[1], Ken-ichi Otake[2], Jia-Jia Zheng[3], Ping Wang[4], Qing Lin[5], Susumu Kitagawa[2] & Cheng Gu[1,4]

*Corresponding to kitagawa@icems.kyoto-u.ac.jp; gucheng@scu.edu.cn

This PDF file includes:

Table of Contents
Supplementary Materials and methods
Supplementary Figures S1 to S56
Supplementary Tables S1 to S5
Supplementary References (S1−S30)

Table of Contents

1. Supplementary Materials and methods...P3−P8
Section 1: Materials and PCP synthesis...P3−P4
Section 2: Instruments and characterizations..P4−P6
Section 3: Theoretical calculation...P6−P7
Section 4: Gas separation...P7−P8

2. Supplementary Figures S1 to S56...P9−P64
Section 1: ^{1}H and ^{13}C NMR, mass spectra, and calculated flip-flop energy of the ligand................P9−P18
Section 2: Morphology, infrared spectra, and crystal structures of as-synthesized FDC–4............P19−P21
Section 3: ^{1}H NMR, TG, crystal structure, and PXRD of activated FDC–4a................................P22−P26
Section 4: Gas-sorption behaviors of FDC–4a...P27−P29
Section 5: *In-situ* PXRD studies...P30−P33
Section 6: Global temperature–diffusion-rate–adsorption amount curves for FDC–4a..........................P34
Section 7: VT-PXRD and mechanism for C_3H_6 and C_3H_8 adsorption...P35−P37
Section 8: Theoretical calculations..P38−P39
Section 9: Gas separation experiments for FDC–4a...P40−P64

3. Supplementary Tables S1 to S5...P65−P69
Section 1: Structures and physicochemical properties of C_3H_6 and C_3H_8..P65
Section 2: Crystallographic data for as-synthesized and activated FDCs......................................P66−P67
Section 3: Comparison of C_3H_6/C_3H_8 adsorption performances of the benchmark materials.................P68
Section 4: Calculated energies for C_3H_6 and C_3H_8 adsorptions and diffusions in FDC–4a...................P69

4. Supplementary References (S1−S30)...P70−P71

Materials and Methods

Section 1: Materials and PCP synthesis

Materials

Dimethyl 5-iodoisophthalate (98%, Jilin Chinese Academy of Sciences-Yanshen Technology Co., Ltd.), 10-methoxy-5*H*-dibenzo[*b,f*]azepine (98%, TCI), cesium carbonate (98%, TCI), tris(dibenzylideneacetone)dipalladium(0) (75%, TCI), sodium hydroxide (97%, Energy Chemical), 2-dicyclohexylphosphino-2',4',6'-triisopropylbiphenyl (98%, TCI), anhydrous hexane (99.5%, Energy Chemical), anhydrous *N,N*-dimethylformamide (99.5%, Energy Chemical), anhydrous toluene (99.5%, Energy Chemical), anhydrous tetrahydrofuran (99.5%, Energy Chemical), anhydrous methanol (99.5%, Energy Chemical), anhydrous ethyl acetate (99.5%, Energy Chemical), anhydrous magnesium sulfate (99.5%, Energy Chemical), 12 M HCl (Aldrich), 40 wt% NaOD in D_2O (Aldrich), copper(II) nitrate pentahydrate (99.99%, Aldrich), and deuterated solvents for nuclear magnetic resonance (NMR) spectroscopy (Cambridge Isotope Laboratories) were purchased and used without further purification.

Synthesis of MODBAP-ipa ligand

Synthesis of dimethyl 5-(10-methoxy-5*H*-dibenzo[*b,f*]azepin-5-yl)isophthalate (1): Dimethyl 5-iodoisophthalate (19.20 g, 60.0 mmol, 1.2 eq.), 10-methoxy-5*H*-dibenzo[*b,f*]azepine (11.16 g, 50.0 mmol, 1.0 eq.), 2-dicyclohexylphosphino-2',4',6'-triisopropylbiphenyl (XPhos, 1.19 g, 2.5 mmol, 0.05 eq.), tris(dibenzylideneacetone)dipalladium(0) (1.37 g, 1.5 mmol, 0.03 eq.), cesium carbonate (32.58 g, 100.0 mmol, 2.5 eq.), and toluene (200 mL) were placed in a flask whose inner gas was replaced by N_2. The mixture was stirred at 115 °C for 48 h. After cooling down to room temperature, the reaction mixture was filtered through Celite®. The filtrate was diluted with ethyl acetate (200 mL) and washed with water. The organic phase was dried over $MgSO_4$, filtered, and evaporated under reduced pressure. The residue was purified by column chromatography (SiO_2, ethyl acetate/*n*-hexane with the ratio changing from 3 to 6%) to give **1** (11.22 g, yield = 54%) as a light-yellow solid. ^{1}H NMR (500 MHz, DMSO-d_6): δ (ppm) = 7.80 (2H, d, *J* = 11.5 Hz, Ph C_2-*H* and MODBAP C_6-*H*), 7.70 (1H, t, *J* = 7.6 Hz, MODBAP C_9-*H*), 7.60 (1H, d, *J* = 7.9 Hz, MODBAP C_8-*H*), 7.55 (3H, dd, *J* = 15.5, 6.6 Hz, MODBAP $C_{1,4,7}$-*H*), 7.47 (1H, t, *J* = 7.5 Hz, MODBAP C_2-*H*), 7.42 (1H, t, *J* = 7.4 Hz, MODBAP C_3-*H*), 7.04 (2H, s, Ph $C_{4,6}$-*H*), 6.29 (1H, s, MODBAP C_{11}-*H*), 3.75 (9H, s, -CO_2CH_3 and -OCH_3); ^{13}C NMR (126 MHz, DMSO-d_6): δ (ppm) = 165.97, 155.83, 149.06, 135.73, 133.89, 131.97, 131.14, 130.93, 129.73, 129.40, 128.89, 128.61, 128.46, 128.20, 119.35, 115.67, 102.71, 55.94, 52.81; MALDI-TOF-MS: calcd. *m/z* = 415.1420; found *m/z* = 415.654.

Synthesis of 5-(10-methoxy-5*H*-dibenzo[*b,f*]azepin-5-yl)isophthalic acid (MODBAP-ipa): To the THF/MeOH (200 mL, 1/1 v/v) solution containing **1** (10.0 g, 25.9 mmol) was added 2 M NaOH aqueous solution (200 mL, 400 mmol) and the system was reflexed for 16 h. After cooling to 0 °C, the reaction mixture was acidified with concentrated HCl. The precipitate was collected by filtration, washed with water, and then dried under reduced pressure at 60 °C to give **MODBAP-ipa** (8.0 g, yield = 90%) as a white solid. ^{1}H NMR (500 MHz, DMSO-d_6): δ (ppm) = 13.03 (2H, s, -CO_2H), 7.83-7.75 (2H, m, Ph C_2-*H* and MODBAP C_6-*H*), 7.68 (1H, t, *J* = 7.6 Hz, MODBAP C_9-*H*), 7.59 (1H, d, *J* = 7.9 Hz, MODBAP C_8-*H*), 7.53 (3H, dt, *J* = 15.6, 7.8 Hz, MODBAP $C_{1,4,7}$-*H*), 7.46 (1H, t, *J* = 7.4 Hz, MODBAP C_2-*H*), 7.40 (1H, t, *J* = 7.4 Hz, MODBAP C_3-*H*), 7.03 (2H, s, Ph $C_{4,6}$-*H*), 6.29 (1H, s, MODBAP C_{11}-*H*), 3.76 (3H, s, -OCH_3); ^{13}C NMR (126 MHz, DMSO-d_6): δ = 167.21, 155.84, 148.88, 142.12, 140.43, 135.81, 133.99, 132.15,

131.92, 130.89, 129.89, 129.55, 128.84, 128.56, 128.32, 128.08, 115.76, 102.78, 55.94; MALDI-TOF-MS: calcd. *m/z* = 387.1107; found *m/z* = 387.603.

Synthesis of **FDC–4**

Firstly, 50 mg (0.13 mmol) **MODBAP-ipa** was dissolved in 6 mL DMF at room temperature. An aqueous solution (4 mL) of $Cu(NO_3)_2 \cdot 3H_2O$ (62.4 mg, 0.26 mmol) was added to the above solution. Then the mixture was heated at 80 °C for 24 h. **FDC–4** was obtained as green lamellar crystals with sizes up to several hundreds of micrometers (70 mg, yield = 65%). The crystals were filtered, washed with DMF (10 mL, 3 times) and H_2O (10 mL, 3 times), and dried in air. The as-synthesized **FDC–4** was characterized by infrared spectra (Supplementary Fig. 12). The adsorption peak of the stretching vibration of the C=O double bond shifted to a low wavenumber, indicative of the coordination bond formation in **FDC–4**.

Solvent exchange and activation of **FDC–4**

To measure the adsorption property of **FDC–4**, we exchanged the guest and coordination solvents (DMF) with methanol by soaking **FDC–4** in methanol at 60 °C for 7 days. Every 24 h the methanol was replaced by a new one. After the solvent exchange, the exchanged **FDC–4** was dried under vacuum at 60 °C for 3 h. ^{1}H NMR confirmed that the DMF in the exchanged **FDC–4** was exchanged by methanol (Supplementary Fig. 14).

TG curve showed that the framework of the exchanged **FDC–4** was thermally stable until 170 °C (Supplementary Fig. 15). Thus, we activated the exchanged **FDC–4** at 120 °C for 11 h to afford **FDC–4a**; this temperature ensured the complete removal of the solvents meanwhile excluding the possibility of framework decomposition.

Section 2: Instruments and characterizations

General instrumental analysis

^{1}H NMR spectra were recorded at 25 °C on a Brucker models Ultrashild 500 Plus NMR spectrometer operating at 500 MHz, where chemical shifts (δ in ppm) were determined with respect to tetramethylsilane (TMS) as an internal reference. The mass measurements for solid-state samples were carried out on a rapifleXTM MALDI-TOF/TOF mass spectrometer from Bruker Daltonik GmbH using 7,7,8,8-tetracyanoquinodimethane (TCNQ) as the matrix. IR spectra were obtained with a Thermo Scientific Nicolet Summit FT-IR equipped with a diamond ATR accessory under an ambient atmosphere. Thermogravimetric analysis (TGA) was performed on a Rigaku Thermo plus EVO2 under a nitrogen atmosphere with a temperature ramp of 5 °C min^{-1}.

X-ray diffraction

Single-crystal X-ray diffraction (SCXRD) for **FDC–4** was performed on a Rigaku XtaLAB P200 diffractometer equipped with a Dectoris PILATUS 200 K detector, using a VariMax Mo Optic with Mo−Kα radiation (λ = 0.71075 Å). The structure was solved using direct methods and refined by full-matrix least-squares cycles in SHELX 2014/7[S1]. All non-hydrogen atoms were refined using anisotropic thermal parameters.

Powder X-ray diffraction (PXRD) measurements were performed on a Rigaku SmartLab X-ray diffractometer using Cu−Kα radiation (λ = 1.54178 Å) in the 2θ range of 4−40° with a scanning rate of 5° min^{-1}. Variable-temperature synchrotron powder X-ray diffraction measurements were conducted using high-resolution one-dimensional semiconductor (MYTHEN) detectors (Dectris, Switzerland) installed in the BL02B2 beamline of SPring-8 (λ = 0.80000 Å) at varied

temperatures[S2,S3]. The crystalline powder of **FDC–4a** in a borosilicate glass capillary (0.5 mm inside diameter) was activated at 393 K under vacuum for 1 h. The measurement temperature was changed from 375 to 90 K at the cooling rate of 10 K min^{-1} with nitrogen flow.

Continuous rotation electron diffraction (cRED)

The crystal powder was drop-casted onto a copper grid (R1.2/1.3, QUANTIFOIL), and the grid was plunged into liquid nitrogen rapidly. The grid was then transferred to the Fischone 2550 cryo holder and TEM at liquid nitrogen temperature (100 K). The cRED data were collected on a JEOL 2100-plus TEM equipped with MerlinEM direct electron detector under 200 kV acceleration voltage and installed with Heimdall data collection software (software developed by the ReadCrystal Tech Co.). The data were visualized with the program REDp (Wan et al., 2013) and processed using XDS (Kabsch, 2010) with the aid of Coeus (software developed by the ReadCrystal Tech Co.)[S4].

Crystal structure models were built directly using the Shelxt package, and more accurate structure models were obtained by the ShelxL refinement.

Gas sorption and *in-situ* PXRD measurements

Gas sorption measurements were performed on BELSORP-max and BELSORP-18PLUS (MicrotracBEL, Japan, Corp.) automated volumetric sorption analyzers, equipped with cryostatic temperature controllers.

The *in-situ* PXRD/adsorption measurements were carried out using a Rigaku SmartLab with Cu−Kα radiation connected to BELSORP-18PLUS volumetric adsorption equipment. Those apparatuses were synchronized with each other and each PXRD pattern was obtained at each point of the sorption isotherms or isobars.

Quantification of the diffusion rate

The diffusion rate was measured on a BEL-18 (BEL Japan, Inc.) automated volumetric sorption analyzer and was fitted automatically with BEL-Dyna software according to the Crank theory[S5] described as follows:

Adsorption rate equation in consideration of in-particle diffusion (assuming spherical particle):

$$\frac{\partial q}{\partial t} = D_s\left(\frac{\partial^2 q}{\partial r^2} + \frac{2}{r} \times \frac{\partial q}{\partial r}\right) \tag{1}$$

When boundary condition $r = R$, $q = q_{0n}$

Linear equilibrium equation:

$$q^* = H \times P \tag{2}$$

Batch adsorption operating equation:

$$W(q - q_{0n}) = V(P_{0n} - P) \tag{3}$$

where

D_s: surface diffusion coefficient [cm^2 s^{-1}]
H: equilibrium constant [cm^3 g^{-1}]
P: pressure [Pa]
P_0: initial pressure [Pa]
P_e: equilibrium pressure [Pa]
q: adsorbed amount [cm^3 g^{-1}]
q_0: initial adsorption amount [cm^3 g^{-1}]
R: radius of particle [cm]
t: time [s]
V: gas phase volume [cm^3]
W: adsorbent amount [g]

By solving simultaneous equations of the above (1), (2), and (3), the following solution is obtained:

$$\frac{P}{P_{0n}} = 1 - \frac{1}{\alpha + 1}\left\{1 - \sum_{n=1}^{\infty} \frac{6\alpha(\alpha+1)\exp(-q_n^2 \tau_s)}{9 + 9\alpha + q_n^2 \alpha^2}\right\} \tag{4}$$

Equation (4) is called the Crank equation.

$$\frac{P - P_{en}}{P_{0n} - P_{en}} = 1 - \left(\frac{P_{0n} - P_{en-1}}{P_{0n} - P_{en}}\right)\left(\frac{1}{\alpha + 1}\right)\left\{1 - \sum_{n=1}^{\infty} \frac{6\alpha(\alpha+1)\exp(-q_n^2 \tau_s)}{9 + 9\alpha + q_n^2 \alpha^2}\right\} \tag{5}$$

Providing that P = CRT, equation (5) is as follows:

$$\frac{C - C_{en}}{C_{0n} - C_{en}} = 1 - \left(\frac{C_{0n} - C_{en-1}}{C_{0n} - C_{en}}\right)\left(\frac{1}{\alpha + 1}\right)\left\{1 - \sum_{n=1}^{\infty} \frac{6\alpha(\alpha+1)\exp(-q_n^2 \tau_s)}{9 + 9\alpha + q_n^2 \alpha^2}\right\} \tag{6}$$

where

$$\tau_s = \frac{D_s}{R^2} t \,, \ \alpha = \frac{V}{W \times H}$$

For the adsorption rate analysis program, equation (6) is used.

The diffusion rate was simultaneously measured with every plot in the adsorption curves and was quantified in the temperature range of 240 to 360 K.

Section 3: Theoretical calculation

Theoretical calculation

The binding energy and diffusion barrier for adsorption and transport of C_3H_6 and C_3H_8 in **FDC–4a** were calculated using the spin-polarised density functional theory (DFT) method with periodic boundary conditions as implemented in the Vienna Ab initio Simulation Package (VASP 5.4.4)[S6,S7]. The primitive unit cell was used in these calculations (Supplementary Fig. 30). Because the adsorption amount of C_3H_8 is very small over a broad temperature range with little lattice changes, we considered only gas adsorption and transport in the activated phase of **FDC–4a**. To seek the adsorption positions of gas molecules, we carried out canonical Monte-Carlo (MC) simulations[S8], as implemented in RASPA[S9]. The Lennard-Jones (LJ) potentials were used to describe the Van der Waals interaction of gas molecules with the **FDC–4a** framework, where the LJ parameters for **FDC–4a** framework were taken from the standard universal force field (UFF)[S10] and those of C_3H_6 and C_3H_8 were taken from the TraPPE force field[S11,S12]. In the MC simulation, the first 1×10^5 cycles were consumed for obtaining equilibration and then the 3×10^5 cycles calculation was performed for seeking the best adsorption position. The final gas adsorption configuration obtained by the above MC simulation was used to construct the initial structure for performing geometry optimization with density functional theory.

The Perdew-Burke-Ernzerhof functional[S13] with Grimme's semiempirical "D3" dispersion term[S14] (PBE-D3) was employed. Plane-wave basis sets with an energy cut-off of 500 eV were used to describe valence electrons, while core electrons were described by the projector-augmented-wave pseudopotentials[S15,S16]. The Brillouin zone was sampled by a Γ-point because the cell size of **FDC–4a** is too big for DFT calculations. During the geometry optimization, only atomic positions were optimized until all atomic forces become smaller than 0.02 eV/Å because there is little change in crystal lattices upon gas adsorption at a low amount. A Hubbard *U* correction[S17] with the *U* value of 4.0 eV[S18] was applied to the localized *d* electrons of the Cu^{2+} center.

The binding energy (BE) of the gas molecule with **FDC–4a** was calculated with equation (7):

$$\mathrm{BE} = E_{\mathrm{PCP{\bullet}L}} - E_{\mathrm{PCP}} - E_{\mathrm{L}} \tag{7}$$

where $E_{\mathrm{PCP{\cdot}L}}$, E_{PCP}, and E_{L} are the energies of **FDC–4a** with 1 gas molecule L (L = C_3H_6 or C_3H_8), empty **FDC–4a**, and one free gas molecule, respectively. The SCS-MP2 correction was made to the binding energy calculated by equation (7), using cluster models shown in Supplementary Fig. S30. In these cluster models, the dangling bonds were capped with H atoms. The resolution of identity (RI) approximation[S19] was employed in SCS-MP2 calculations, as implemented in the PSI4 program[S20]. Dunning's correlation-consistent aug-cc-pVDZ basis sets[S21,S22] were used with appropriate auxiliary basis functions[S23,S24] for RI approximations. Basis set superposition error (BSSE) was corrected using the counterpoise method[S25]. The final adsorption energy was calculated with equation (8):

$$\mathrm{BE}^{\text{SCS-MP2:PBE-D3}} = \mathrm{BE}^{\text{PBE-D3}} + E_{\mathrm{int}}^{\text{SCS-MP2}}(\mathrm{CM}i) - E_{\mathrm{int}}^{\text{PBE-D3}}(\mathrm{CM}i) \tag{8}$$

where $E_{\mathrm{int}}^{\text{SCS-MP2}}(\mathrm{CM}i)$ and $E_{\mathrm{int}}^{\text{PBE-D3}}(\mathrm{CM}i)$ are interaction energies of ethylene molecule with cluster models (i = 1 and 2 for sites I and II, respectively), calculated by the SCS-MP2 and PBE-D3 methods.

The climbing-image nudged elastic band (CI-NEB) method[S26] was used to evaluate the diffusion barriers of C_3H_6 and C_3H_8 in **FDC–4a**. Because there are two kinds of adsorption sites, we considered two possible pathways in which one gas molecule moves from site I to site II and site II to another site II, respectively; the diffusion pathway in which a gas molecule moves directly from site I to another site I is geometrically impossible. In these calculations, 5 images along the diffusion pathways were employed and the convergence criterion for geometry optimization was chosen to be 0.03 eV/Å to save computational time.

The energy change for the flipping of the MODBAP ring in the free MODBAP-ipa ligand was calculated using the Gaussian 16 program[S27]. Geometry optimizations and vibrational frequency calculations were carried out using the DFT method. The dispersion-corrected[S14] B3LYP functional (B3LYP-D3) with the 6-311G(d,p) basis sets for all atoms were used in these calculations. The flipping barrier was estimated by scanning the potential energy surface (PES), in which one dihedral angle ($C^1N^1C^2C^3$, Supplementary Fig. 10) was changed from 90° to 160° with an interval of 10°.

Section 4: Gas separation

Gas separation experiments by temperature-programmed desorption (TPD) protocol

The TPD experiments on **FDC–4a** were carried out as the following steps using BEL-CAT II cooperated with a mass detector (MicrotracBEL, Japan, Corp.). All experimental steps were performed at atmospheric pressure. The steps below were carried out similarly to the steps reported previously[S28,S29].

1. Sample loading and activation:

The powder sample of **FDC–4a** (0.50 g) was filled in the cylindrical cell (8 mmϕ) and activated at 393 K under vacuum for 10 h. The sample cell was then placed on BEL-CAT II. The sample was then *in situ* activated by flowing He at a constant rate of 10 sccm for 1 h at 393 K.

2. Adsorption process:

The temperature was decreased from 393 to 300 K at a rate of 10 K min^{-1}. Once the temperature reached 300 K, the mixed gases of C_3H_6 and C_3H_8 (volume ratios controlled by mass flow controllers, the total flow rate at 10 sccm) flowed at ambient pressure, and the temperature was kept at 300 K for a certain period under flowing mixed gases.

3. Flowing away the non-adsorbed gases:

The remained gases in the cell and gas lines were flowed away by He with a flow rate of 10 sccm for 1 h at 300 K.

4. Release and detection of the adsorbed gases:

After flowing away the non-adsorbed gases, the temperature of the sample cell was increased to 393 K at a rate of 10 K min^{-1} for releasing the adsorbed gases, while the He flow was kept at a 10 sccm flow rate to carry the gases to the detector. The released gases were examined with a mass detector and the ratios between C_3H_6 and C_3H_8 were evaluated from the mass signal. The amounts of released C_3H_6 and C_3H_8 were calculated by integrating the area of the mass signal after the baseline subtraction.

The separation factor α is defined as:

$$\alpha = \frac{X_{C_3H_6} / Y_{C_3H_6}}{X_{C_3H_8} / Y_{C_3H_8}} = \frac{X_{C_3H_6} / X_{C_3H_8}}{Y_{C_3H_6} / Y_{C_3H_8}} \qquad (9)$$

where:

X_{C3H6} = the concentration of C_3H_6 in the adsorbed phase,
Y_{C3H6} = the concentration of C_3H_6 in the feed gas,
X_{C3H8} = the concentration of C_3H_8 in the adsorbed phase,
Y_{C3H8} = the concentration of C_3H_8 in the feed gas.

Breakthrough measurements

The initial activated samples (2.0 g) were tightly packed into a stainless-steel column (ϕ = 3.0 mm, L = 160 mm). The column was activated under vacuum at 393 K with sweeping by He flow to remove impurities for 1 h. Then the gas flow was dosed into the column. Breakpoints were determined by a mass detector. The amounts of desorbed C_3H_6 and C_3H_8 were calculated by integrating the area of the mass signal after the baseline subtraction.

Supplementary Figures

Section 1: ^{1}H and ^{13}C NMR, mass spectra, and calculated flip-flop energy of the ligand

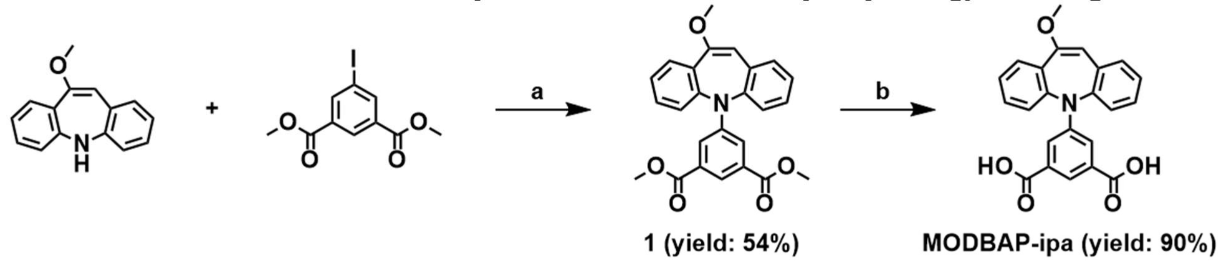

a: $Pd_2(dba)_3$, xphos, Cs_2CO_3, toluene, 115 °C, 48 h.

b: 1) NaOH, THF, MeOH, H_2O, 90 °C, 10 h; 2) HCl.

Supplementary Figure 1. Synthetic routes of MODBAP-ipa.

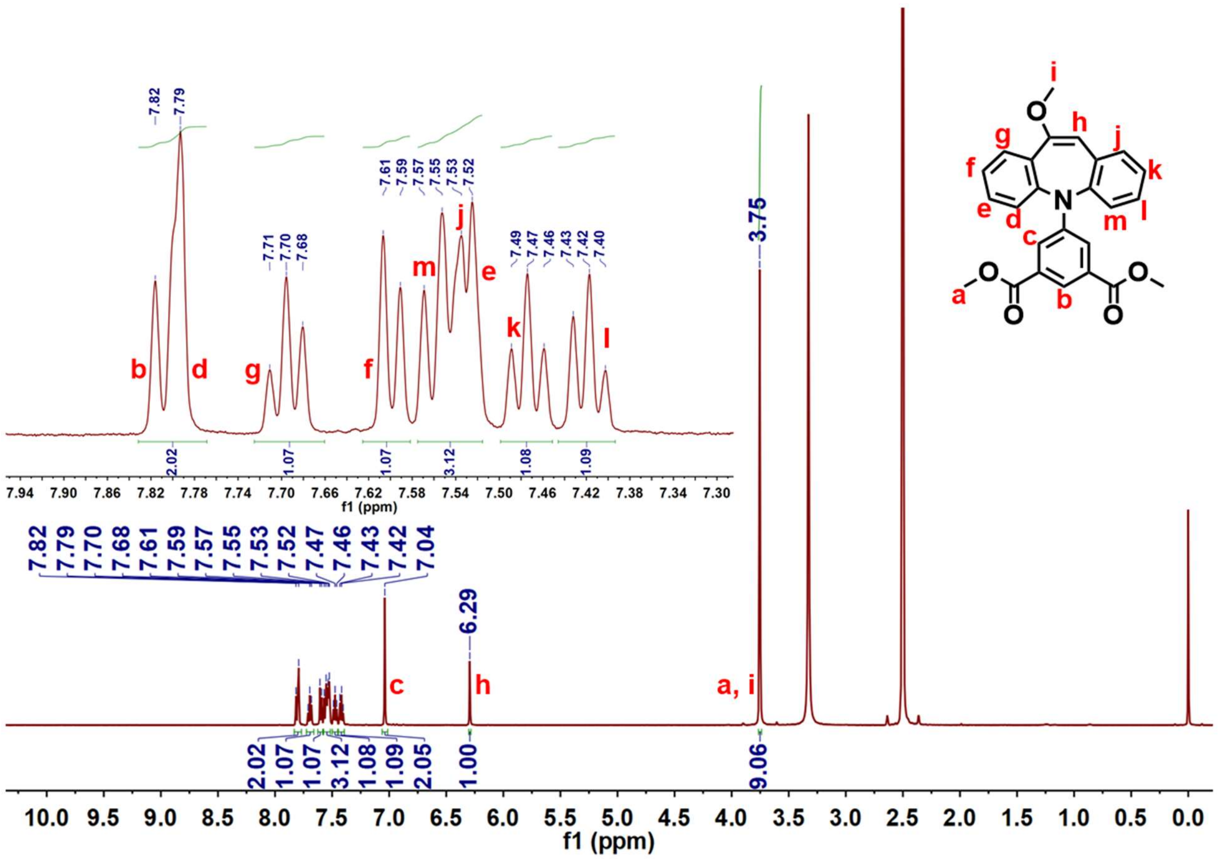

Supplementary Figure 2. ^{1}H NMR spectra of **1**.

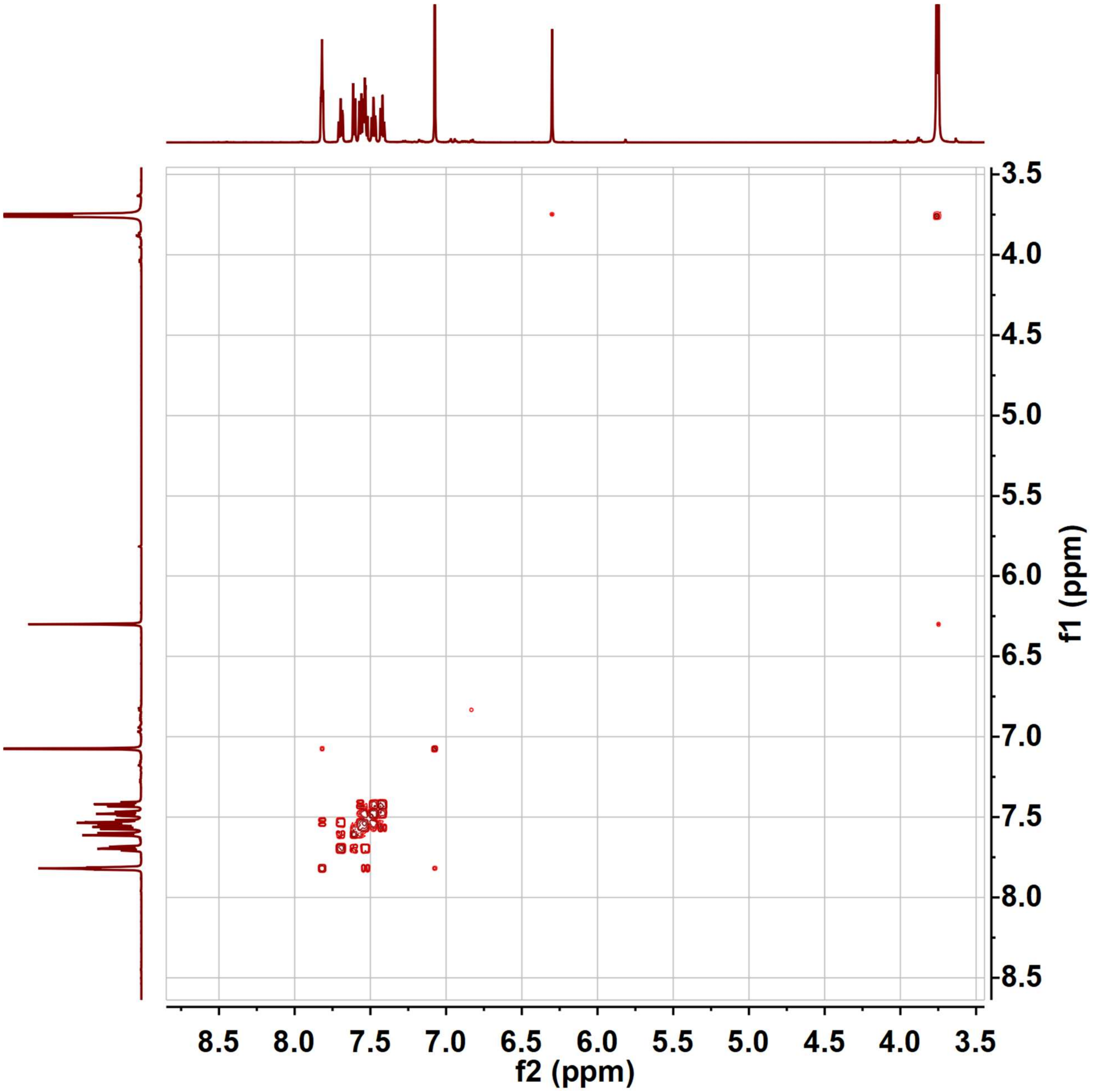

Supplementary Figure 3. ^1H-^1H correlation spectroscopy (COSY) of **1**.

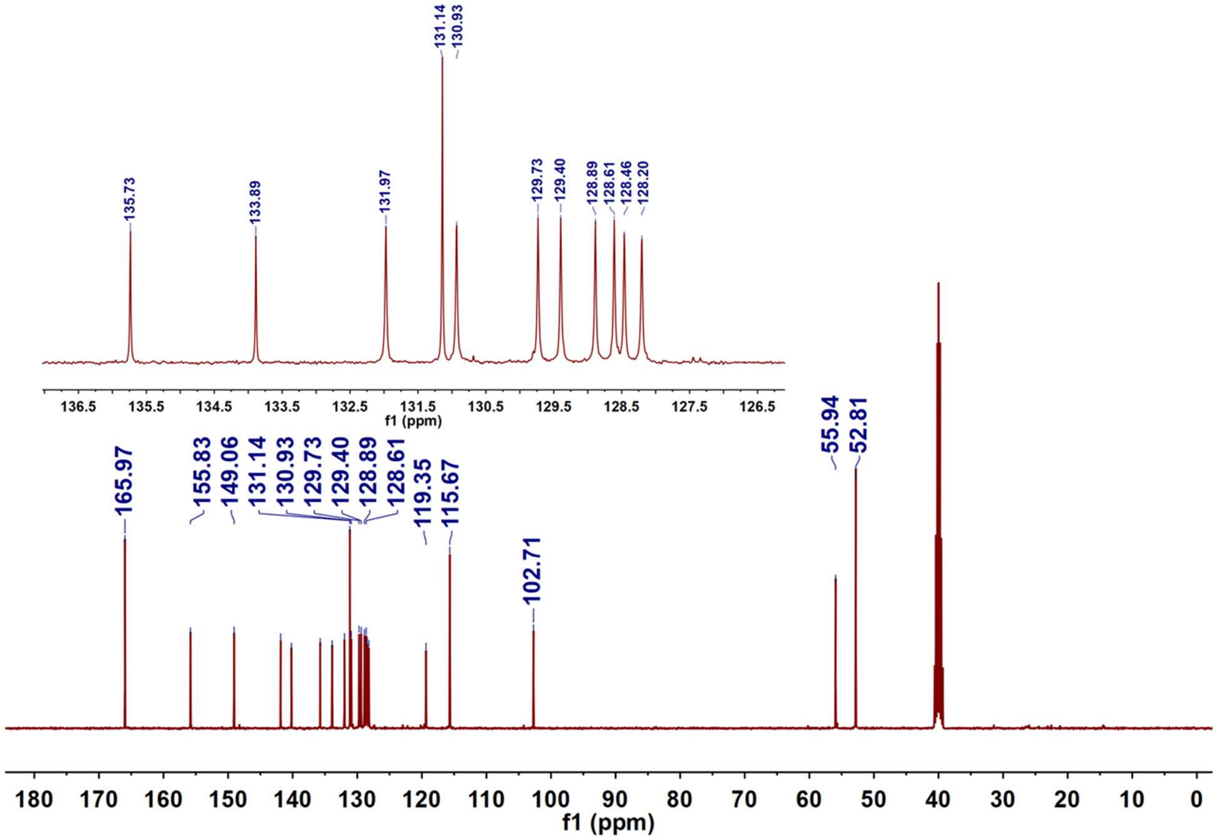

Supplementary Figure 4. ^{13}C NMR spectra of **1**.

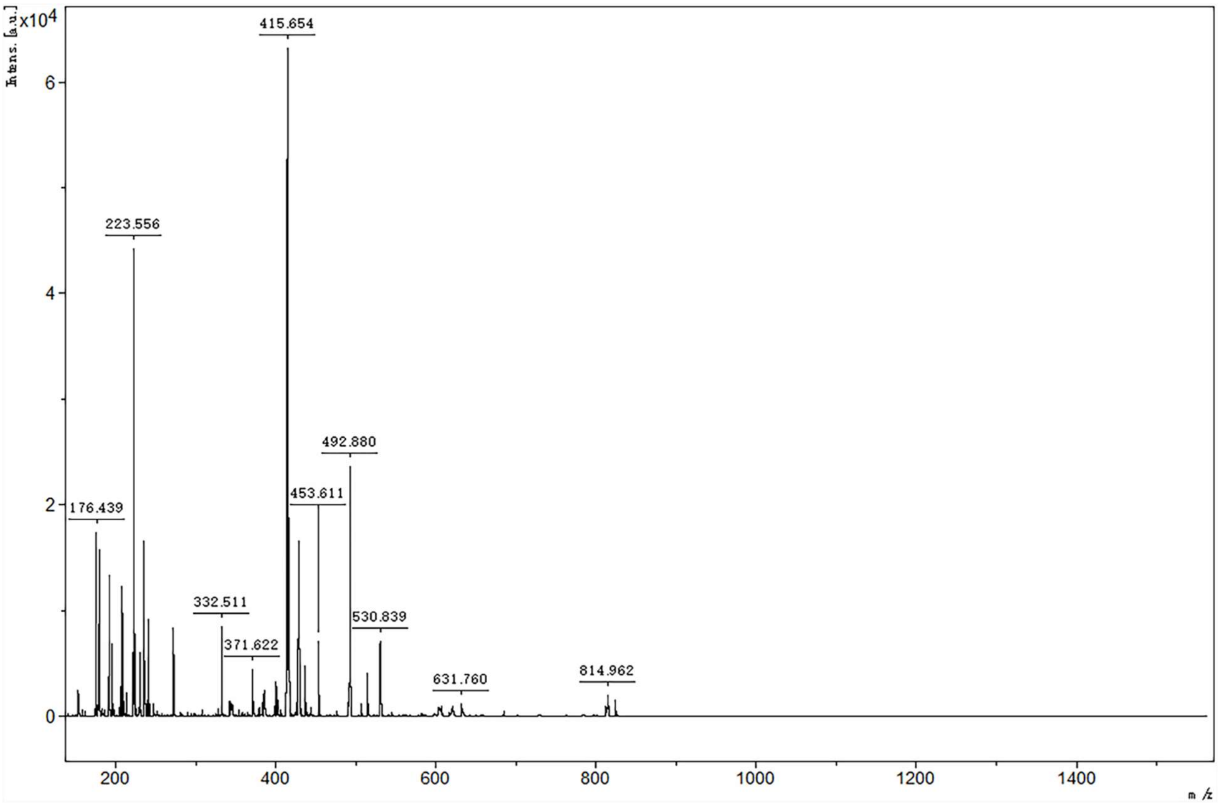

Supplementary Figure 5. MALDI-TOF mass spectra of **1**.

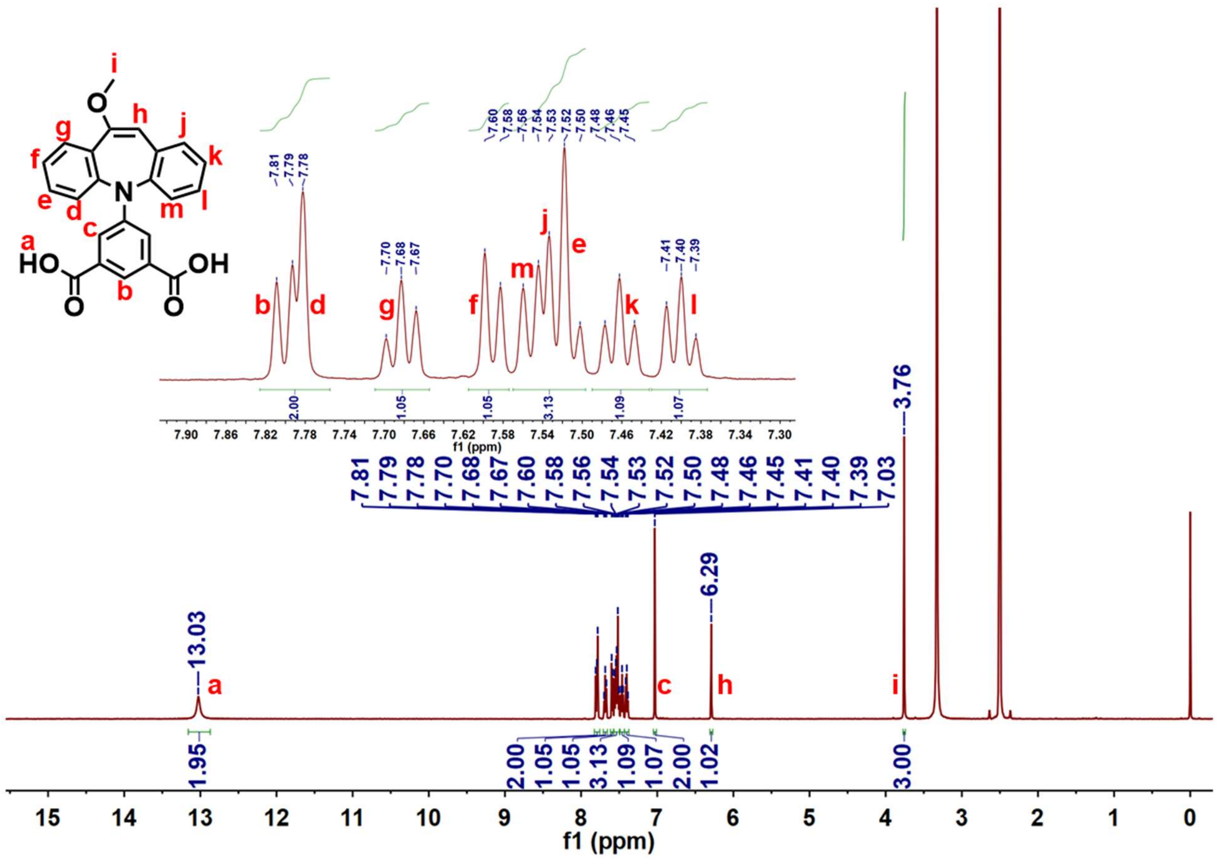

Supplementary Figure 6. ^{1}H NMR spectra of **MODBAP-ipa**.

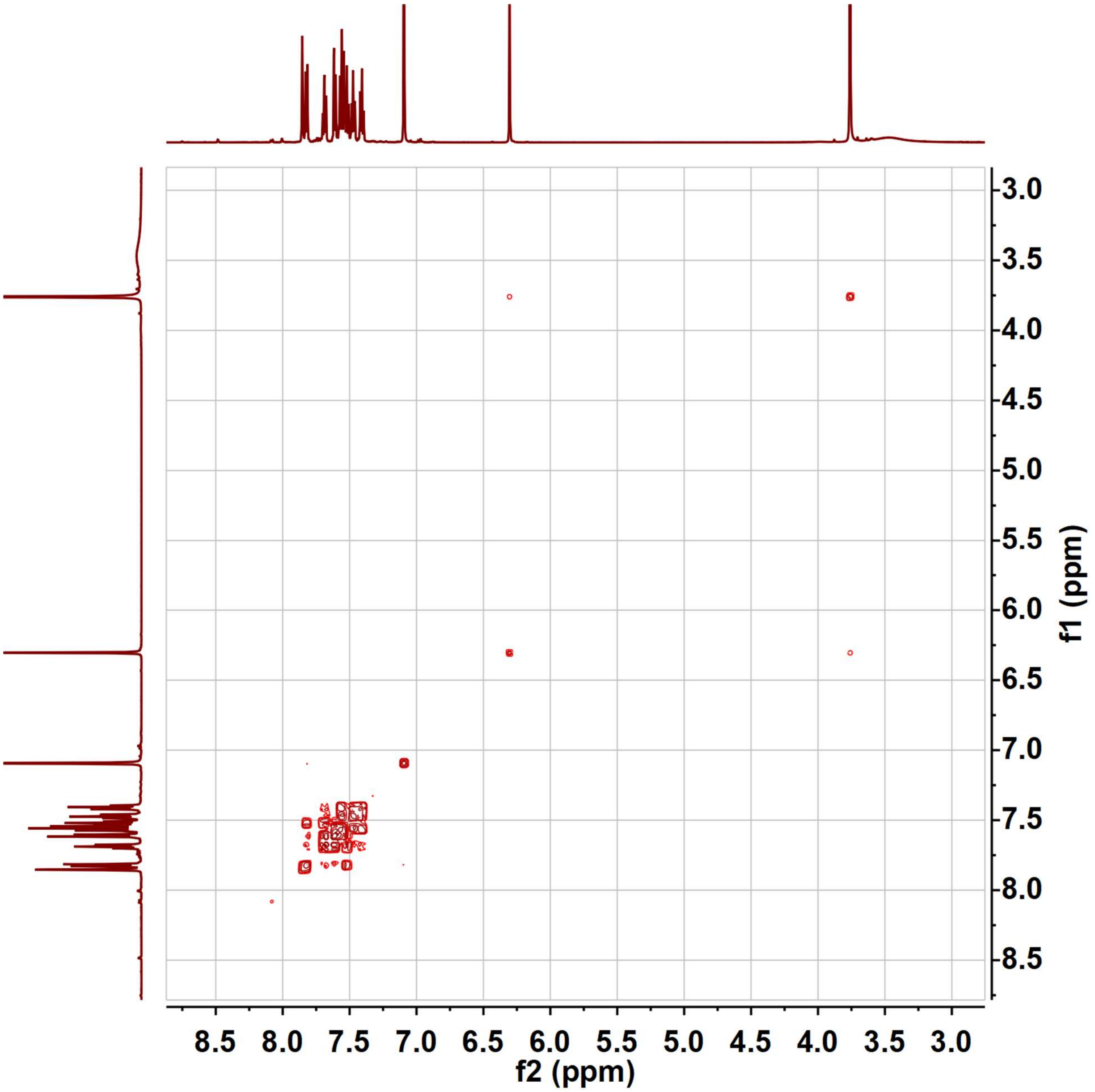

Supplementary Figure 7. ^1H-^1H COSY of **MODBAP-ipa**.

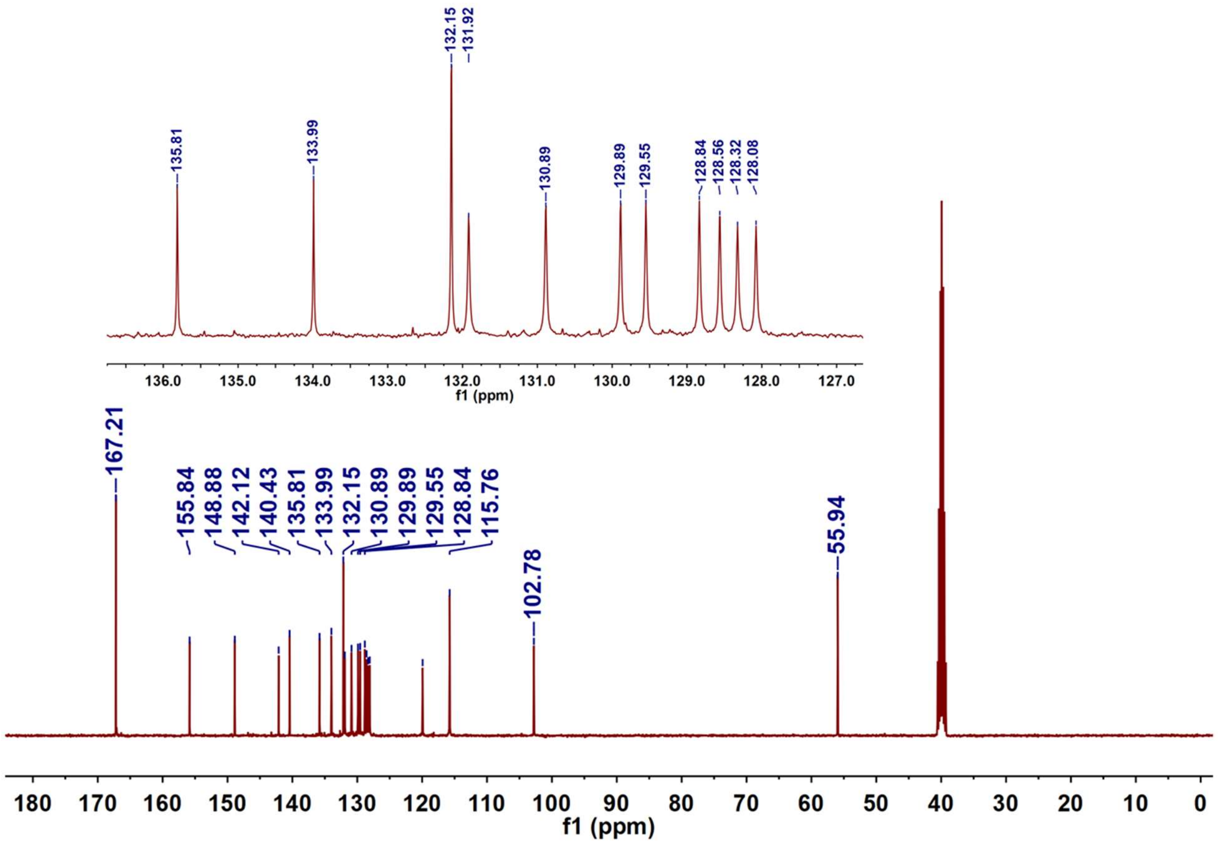

Supplementary Figure 8. ^{13}C NMR spectra of **MODBAP-ipa**.

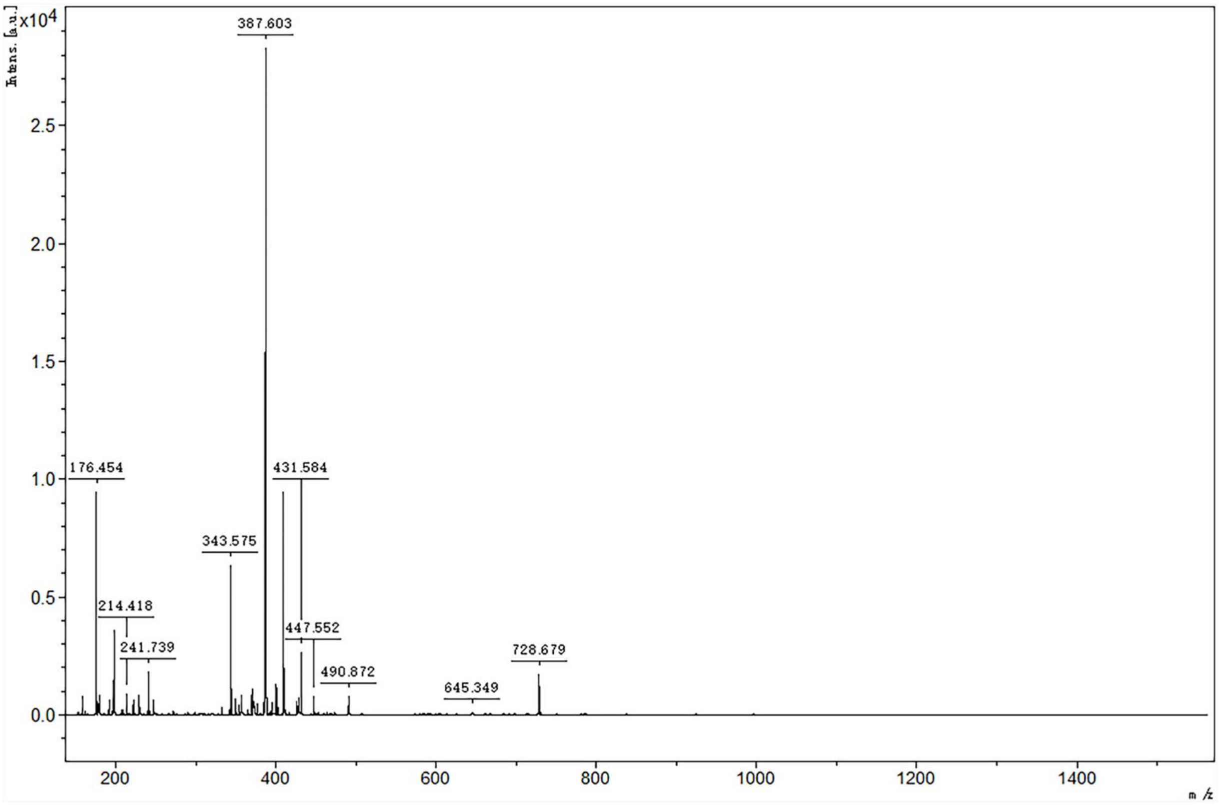

Supplementary Figure 9. MALDI-TOF mass spectra of **MODBAP-ipa**.

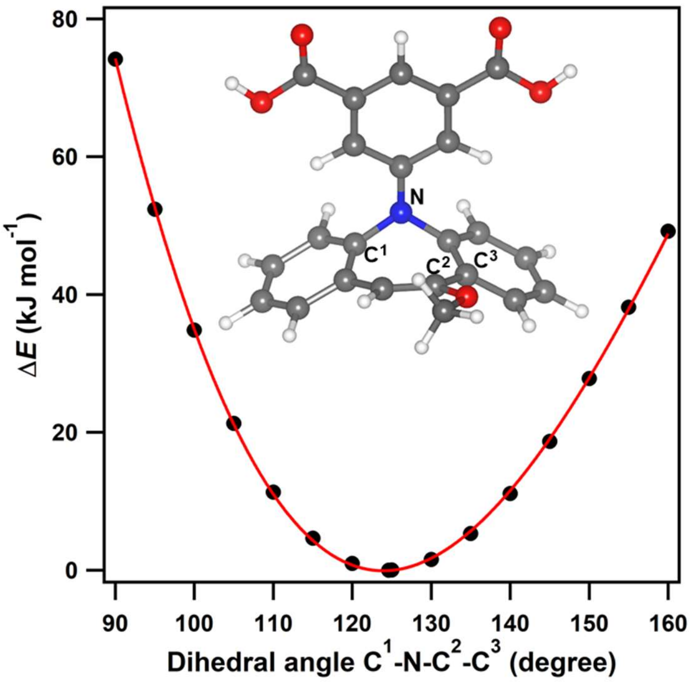

Supplementary Figure 10. Potential energy surface for the flip-flop of MODBAP ring in free ligand molecules. The energy change for the flipping of the MODBAP ring in the free MODBAP -ipa ligand was calculated using the Gaussian 16 program[S14]. The dispersion-corrected[S5] B3LYP functional (B3LYP-D3) with the 6-311G(d,p) basis sets for all atoms were used in these calculations. The ligand flipping was estimated by scanning the potential energy surface (PES), in which one dihedral angle ($C^1NC^2C^3$) was gradually changed from 90° to 160° with an interval of 5°. The energy change (ΔE) for MODBAP ring flipping is less than 40 kJ mol^{-1} with respect to a change of 25° in the dihedral angle. Therefore, the thermal flipping of the MODBAP ring is feasible.

Section 2: Morphology, infrared spectra, and crystal structures of FDCs

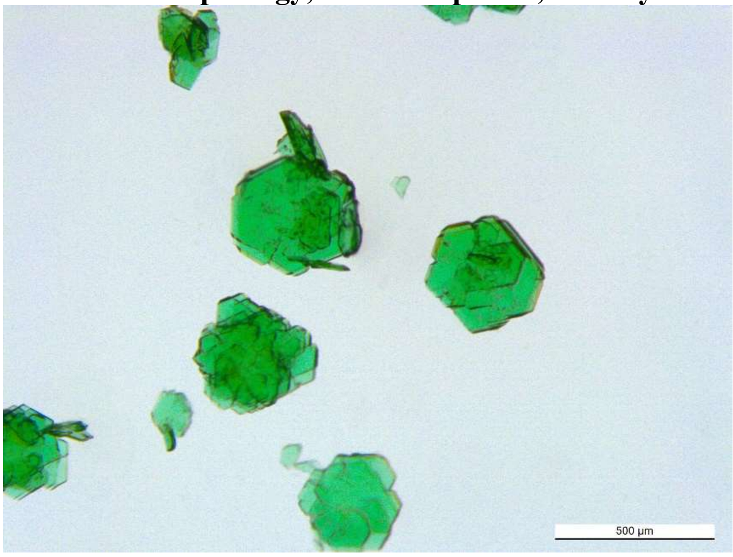

Supplementary Figure 11. Microscopy image of as-synthesized **FDC–4**.

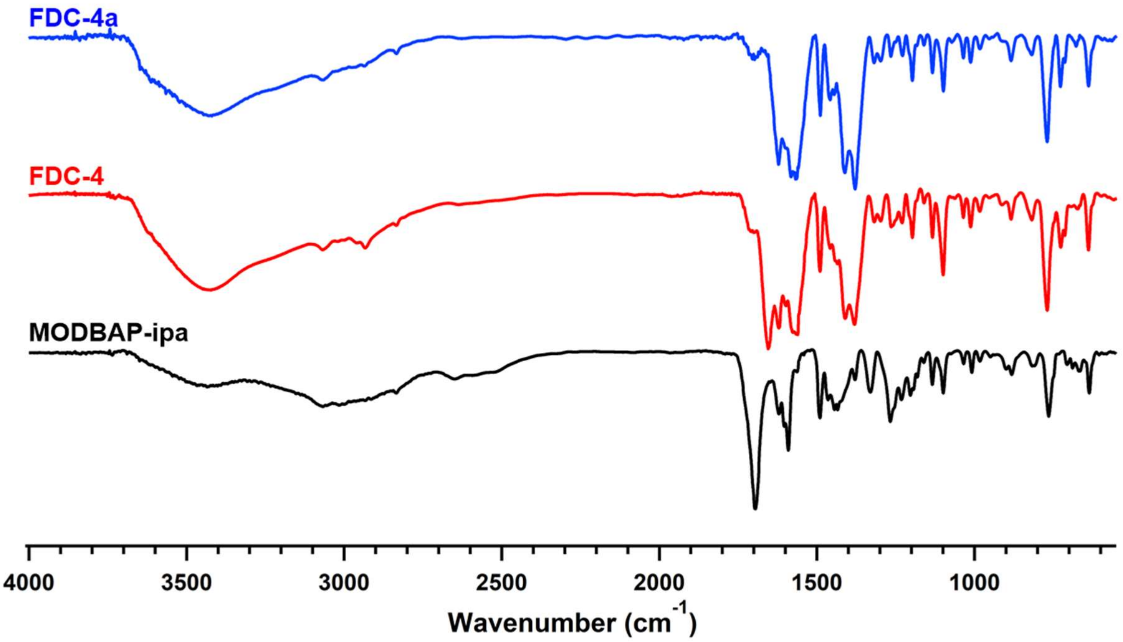

Supplementary Figure 12. Infrared spectra of the MODBAP-ipa ligand, the as-synthesized **FDC–4**, and activated **FDC–4a**.

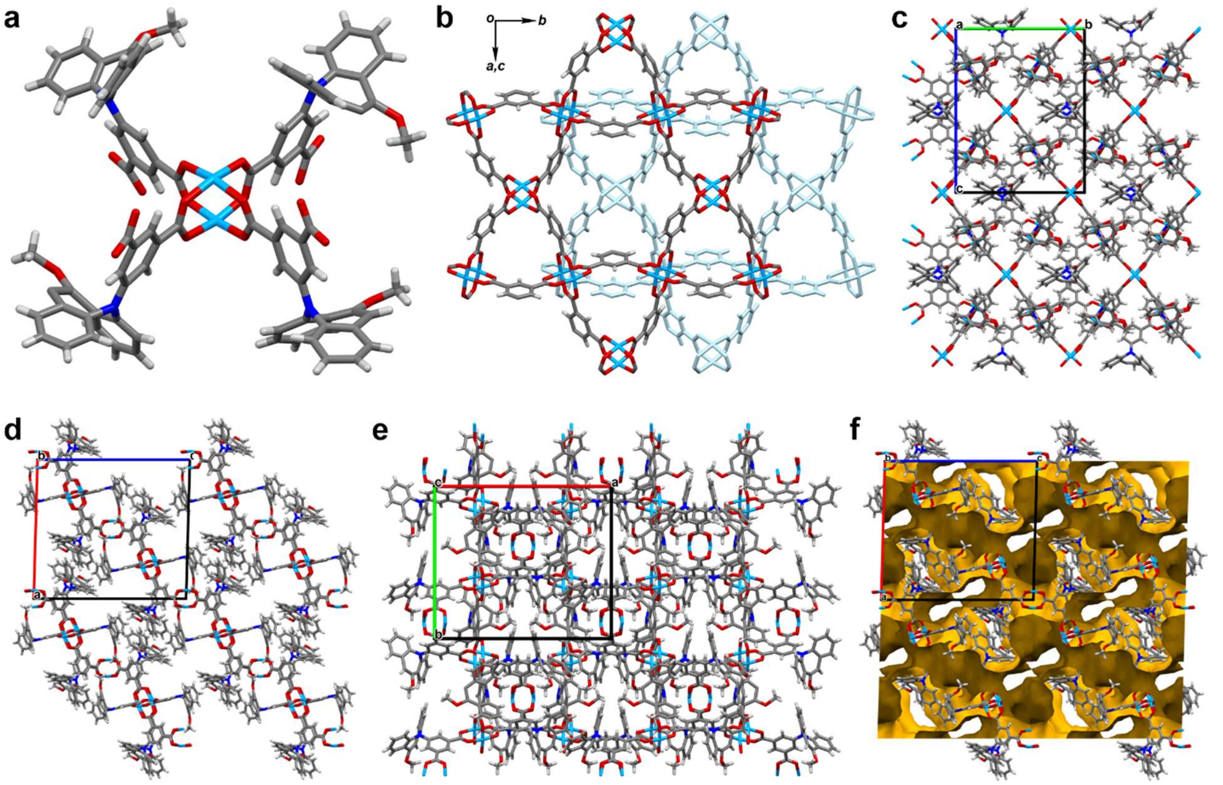

Supplementary Figure 13. Single-crystal structure of as-synthesized **FDC–4**. (a) Cu^{2+} paddle-wheel linked with MODBAP-ipa ligands. (b) Kagomé framework structure of **FDC–4**. For clarity, the MODBAP groups, the coordinated DMF, and H_2O are omitted. The light-blue framework denotes the staggered stacking of the neighboring layers. (c), (d) and (e) represent the view of the crystal structure along the *a*-, *b*-, and *c*-axis, respectively. For clarity, the coordinated DMF and H_2O are omitted. C: grey; N: blue; H: white; O: red; Cu: sky blue. (f) The porous structure of as-synthesized **FDC–4**.

Section 3: ^{1}H NMR, TG, crystal structure, and PXRD of activated FDC–4a

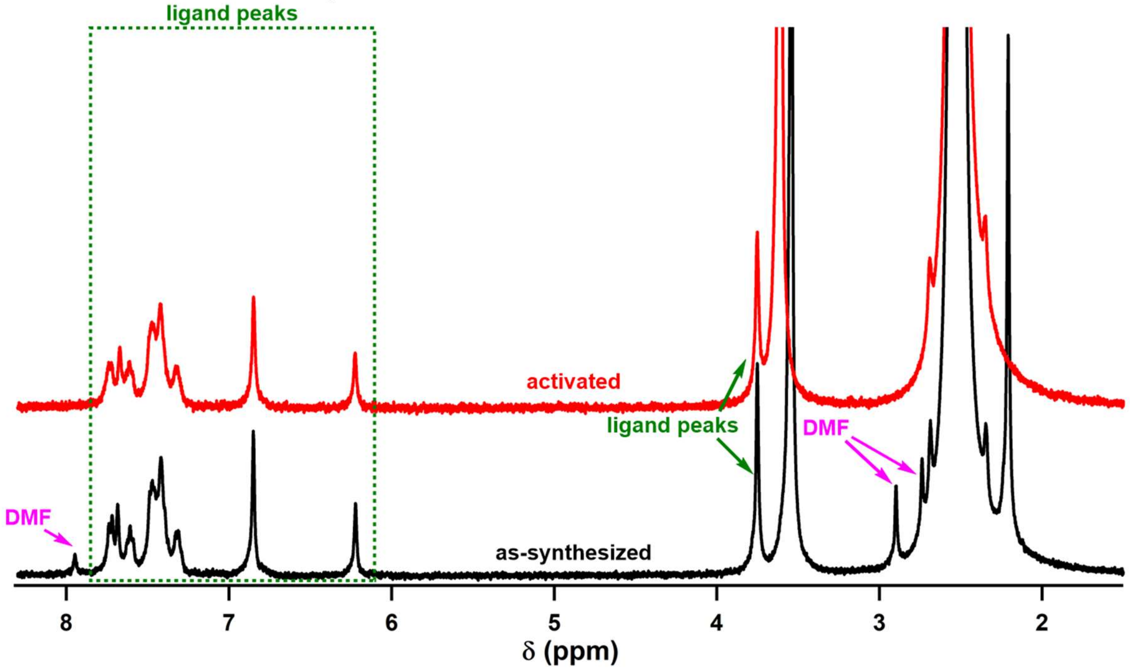

Supplementary Figure 14. ^{1}H NMR of the as-synthesized and activated **FDC–4** dissolved in DMSO-d_6 containing NaOD. The PCP samples were digested by NaOD in DMSO-d_6. The black and red curves represented the as-synthesized and activated PCPs, respectively. The peaks in the green box and green arrows were the peaks of the MODBAP-ipa ligand, whereas the peaks pointed by pink arrows were the peaks of DMF. The NMR clearly showed that the DMF molecules in **FDC–4** were exchanged by methanol.

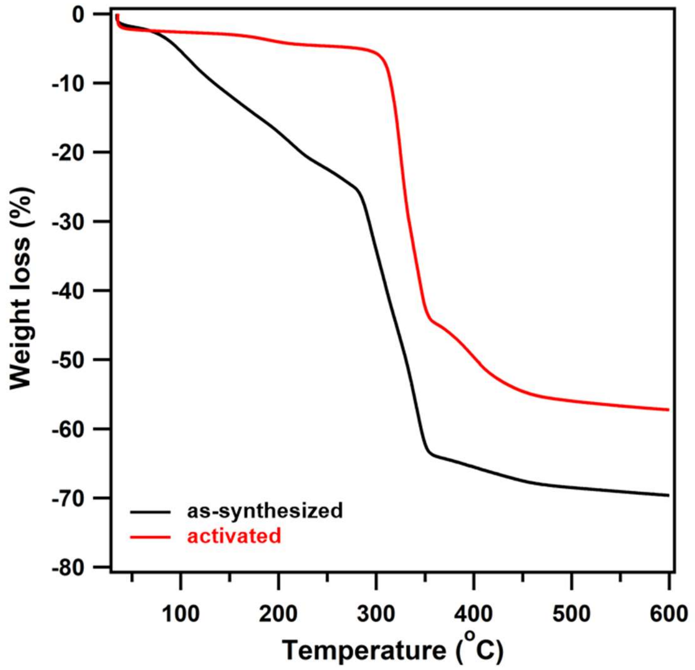

Supplementary Figure 15. TG curves of the as-synthesized and activated **FDC–4**. In the case of the as-synthesized **FDC–4**, the weight loss in the ranges of 70~230 °C corresponded to the loss of water and DMF, respectively. **FDC–4a** was thermally stable until 170 °C.

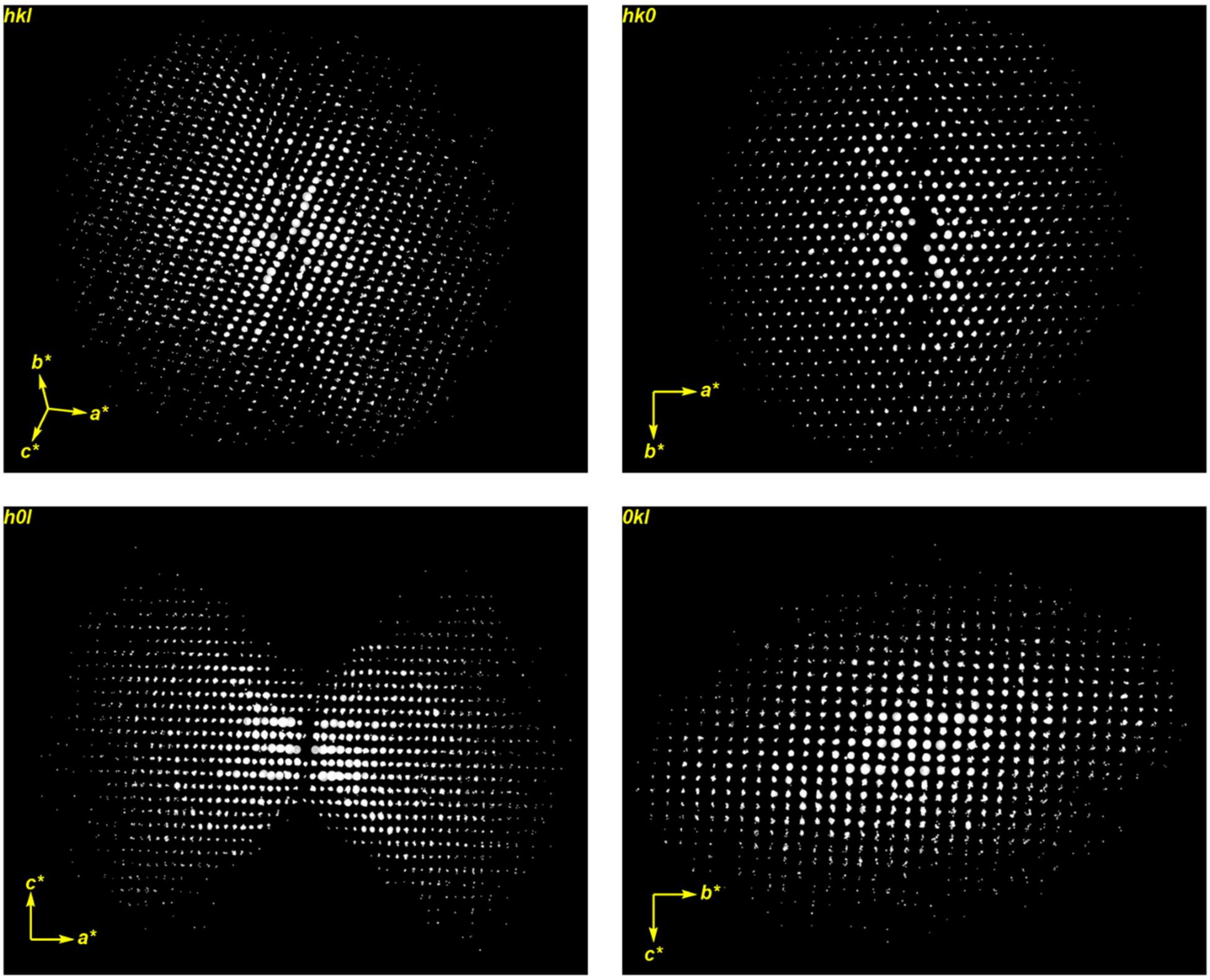

Supplementary Figure 16. The 3D reciprocal lattice of **FDC–4a** by the cRED technique.

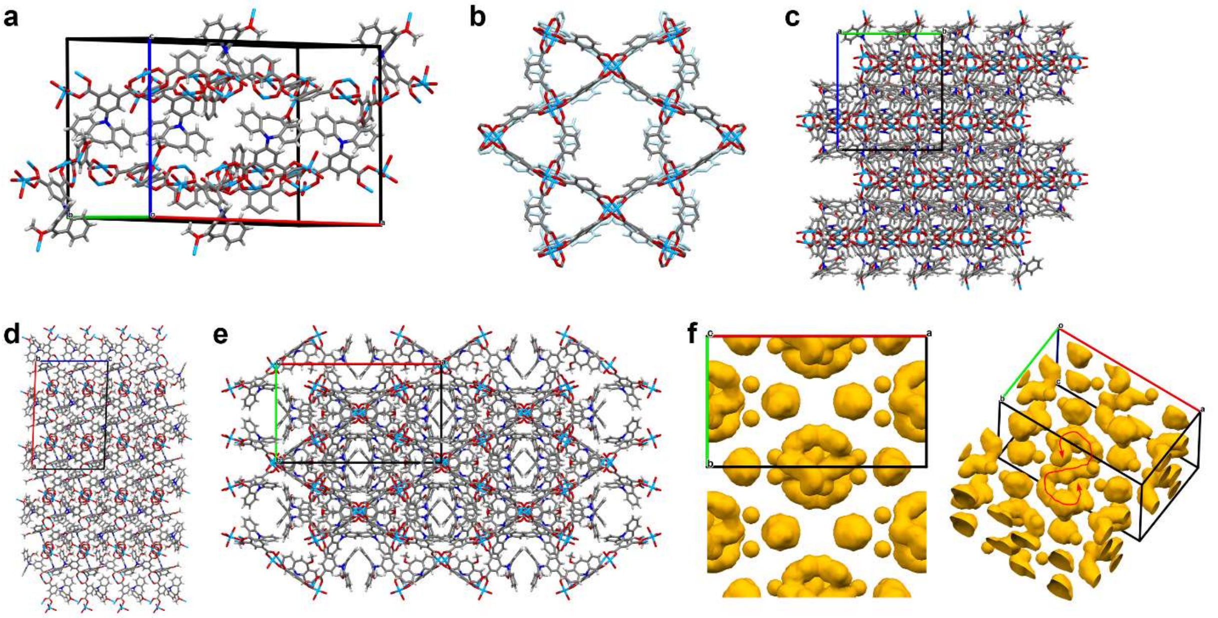

Supplementary Figure 17. Single-crystal structure of activated **FDC–4a**. (a) The O atoms on MODBAP moieties coordinate with the open-metal sites (OMSs) of the Cu^{2+} paddle wheel. For clarity, the MODBAP moieties whose O atoms are uncoordinated with the OMSs of the Cu^{2+} paddle wheels are omitted. (b) Kagomé framework structure of **FDC–4a**. For clarity, the MODBAP groups are omitted. The light-blue framework denotes the eclipsed stacking of the neighboring layers. (c), (d) and (e) represent the view of the crystal structure along the *a*-, *b*-, and *c*-axis, respectively. C: grey; N: blue; H: white; O: red; Cu: sky blue. (f) The porous structure of activated **FDC–4a**.

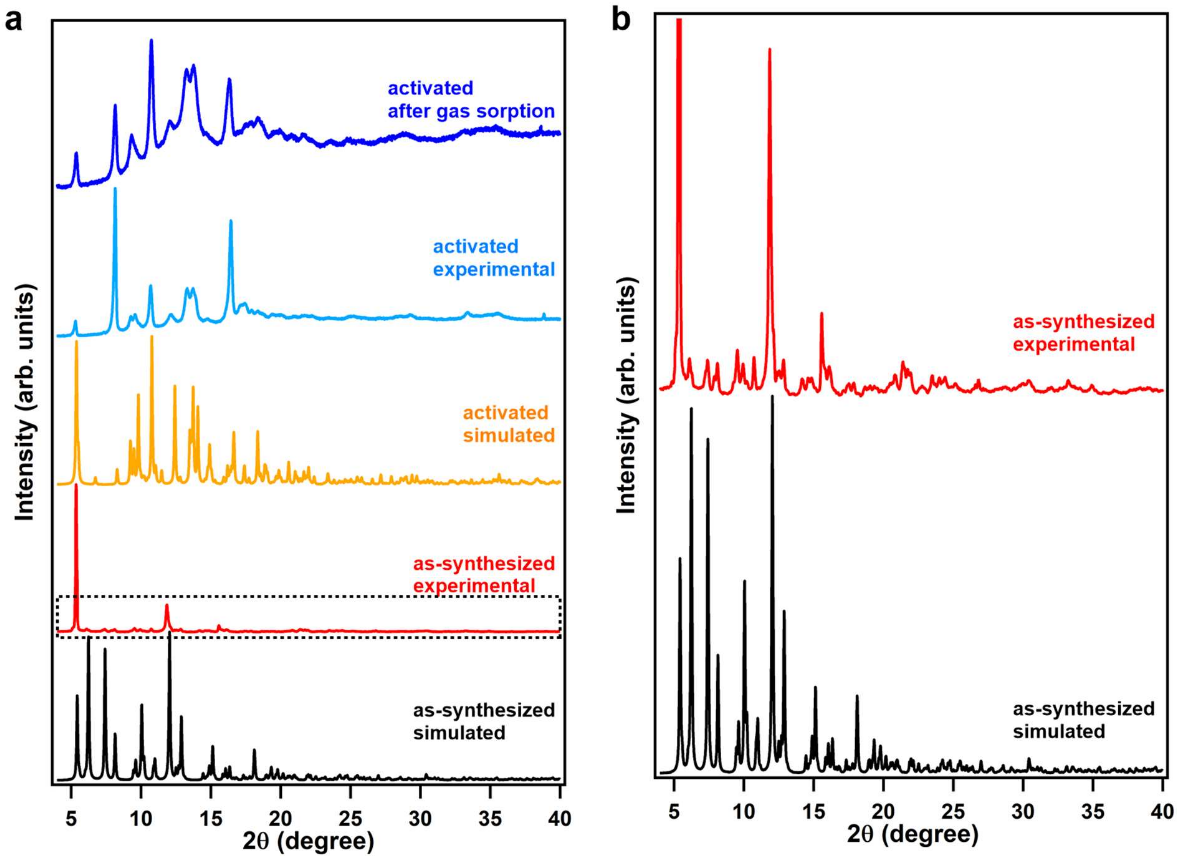

Supplementary Figure 18. (a) PXRD patterns of **FDC–4** (simulated and experimental), **FDC–4a** (simulated and experimental), and **FDC–4a** after gas-sorption experiments. (b) The enlarged experimental PXRD pattern (dotted box in Supplementary Figure 18a) of **FDC–4** and the simulated PXRD pattern. The PXRD patterns of as-synthesized **FDC–4** and activated **FDC–4a** matched well with the simulated ones. On the other hand, the PXRD pattern of the activated **FDC–4a** remained consistent after many times of the gas-sorption processes, indicative of the structural robustness of the activated phase.

Section 4: Gas-sorption behaviors of FDC–4a

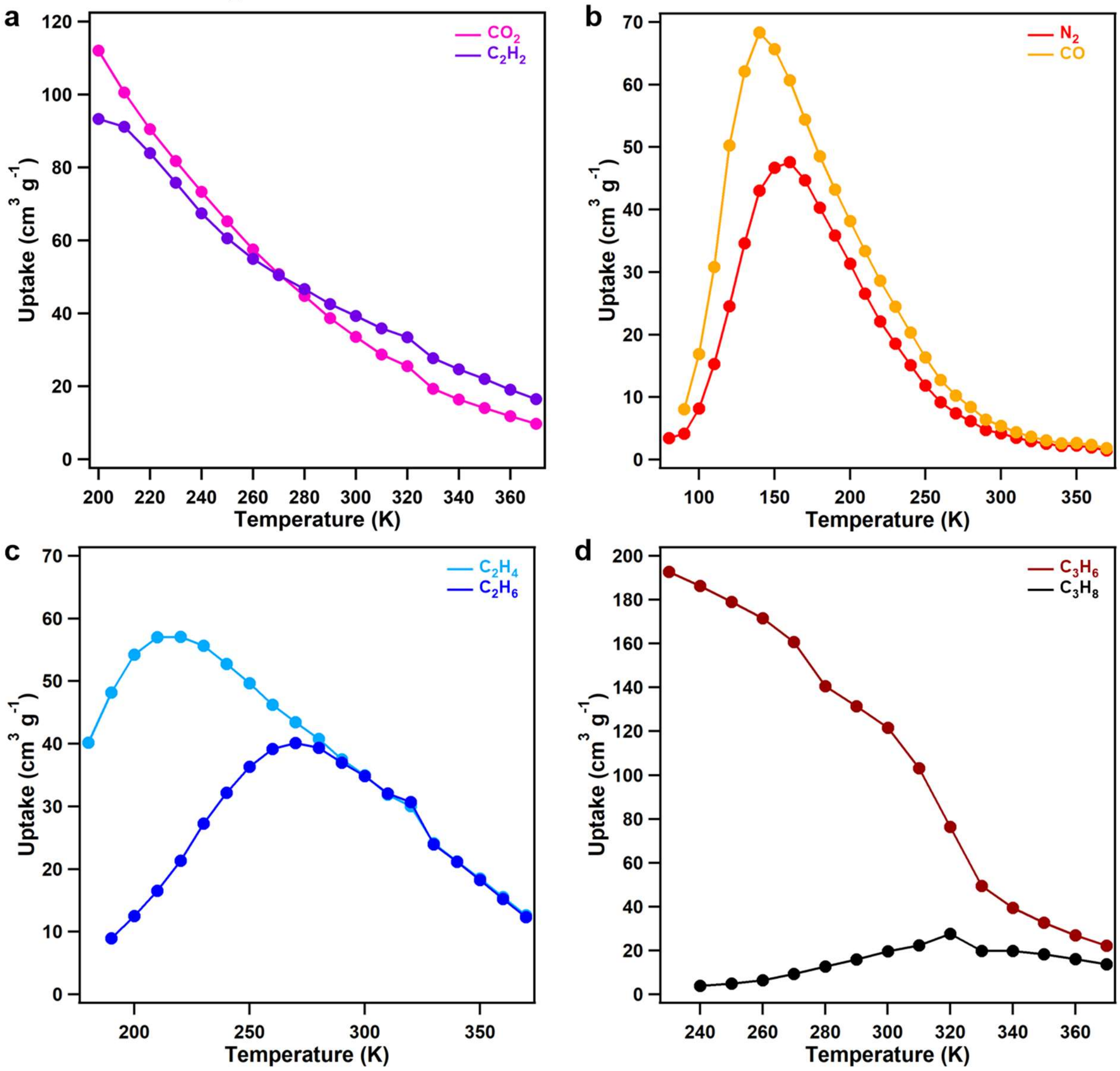

Supplementary Figure 19. Gas-adsorption isobar curves of (a) CO_2 and C_2H_2, (b) N_2 and CO, (c) C_2H_4 and C_2H_6, and (d) C_3H_6 and C_3H_8. The isobar measurements were conducted from low to high temperatures. Note that the starting temperatures were ca. 10 K higher than the T_{bp} of the gases. This parameter setting prevented the condensation of the gases on the surface of **FDC–4a**.

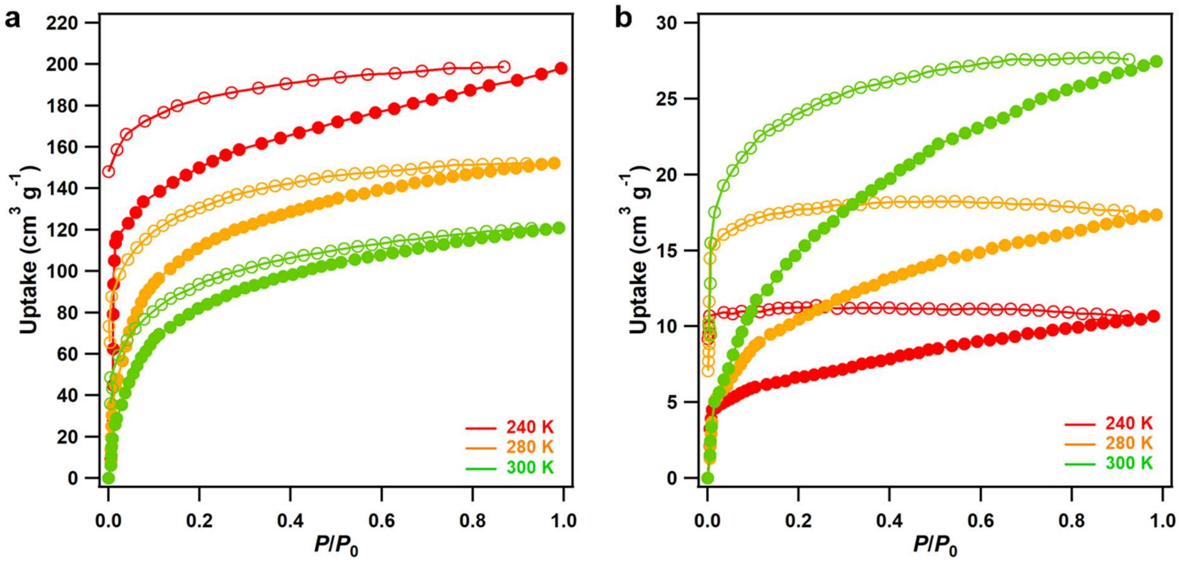

Supplementary Figure 20. (a) C_3H_6- and (b) C_3H_8-sorption isotherm curves at 240, 280, and 300 K.

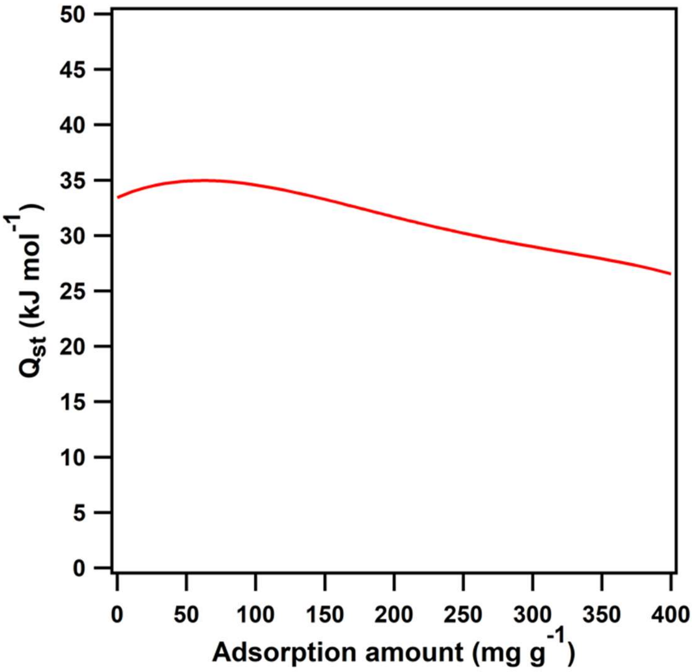

Supplementary Figure 21. Q_{st} curve for C_3H_6 adsorption.

Section 5: *In-situ* PXRD studies

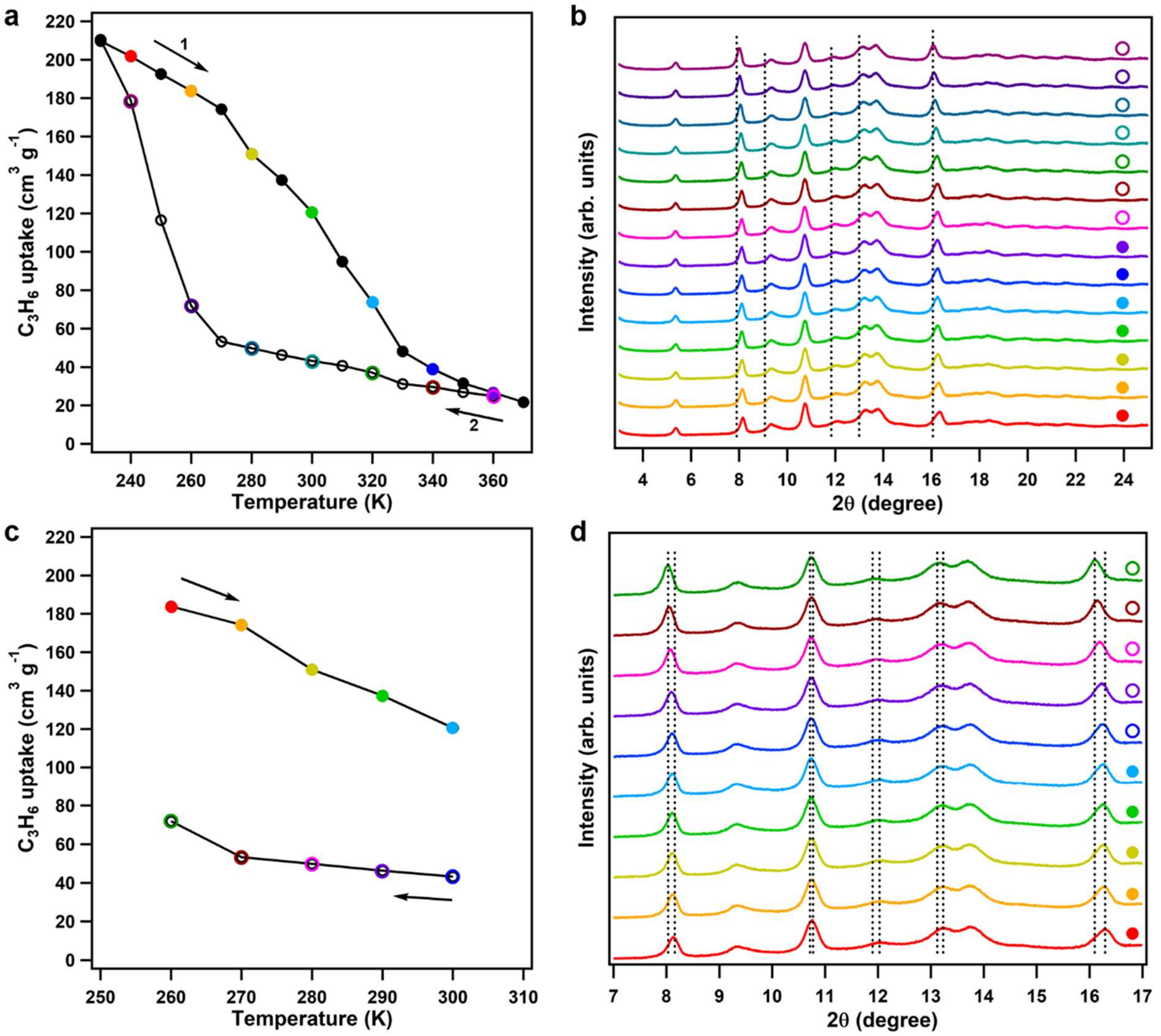

Supplementary Figure 22. (a) C_3H_6-sorption isobar of **FDC–4a** measured in the direction of 230 K → 370 K → 230 K. (b) Coincident *in-situ* isobar/PXRD patterns during C_3H_6 adsorption measured at given temperatures. The adsorptions of 230 K → 370 K and 370 K → 230 K directions showed different paths with almost the same uptakes at 230 K. The adsorptions of 230 K → 370 K and 370 K → 230 K directions showed different paths with almost the same uptakes at 230 K. (c) C_3H_6-sorption isobars of **FDC–4a** individually measured in the direction of 260 K → 300 K and 300 K → 260 K. (d) Coincident *in-situ* isobar/PXRD patterns during C_3H_6 adsorption measured at given temperatures. *In-situ* PXRD suggested continuous structural change during the adsorption process.

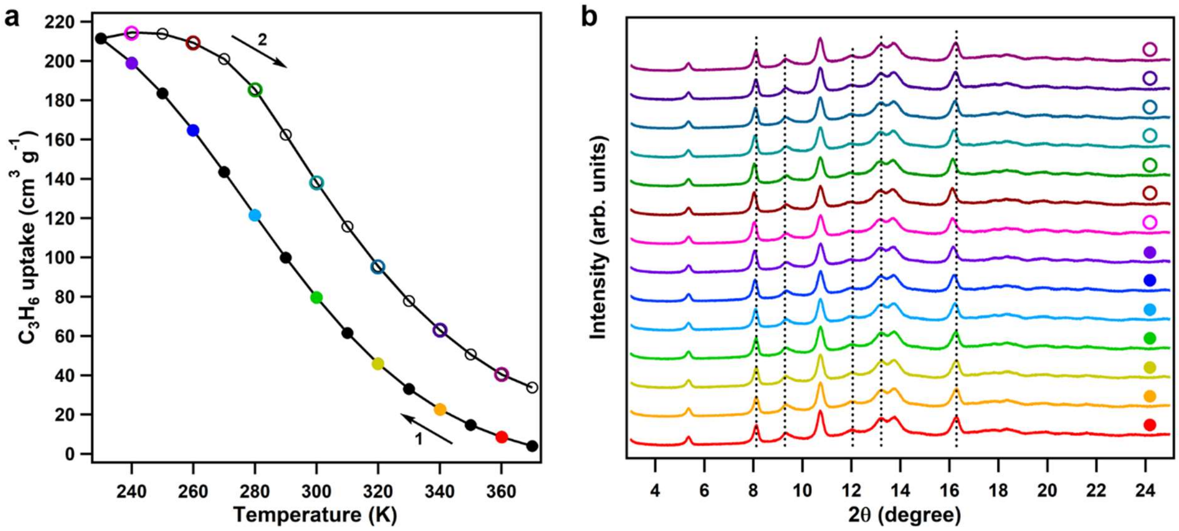

Supplementary Figure 23. (a) C_3H_6-sorption isobar of **FDC–4a** measured in the direction of 370 K → 230 K → 370 K. (b) Coincident *in-situ* isobar/PXRD patterns during C_3H_6 adsorption measured at given temperatures. The adsorptions of 370 K → 230 K and 230 K → 370 K directions showed the same path, whereas the different uptakes at a certain temperature were presumably because of the kinetic factors. *In-situ* PXRD suggested the structural change at low temperatures and the recovery of the structure at high temperatures.

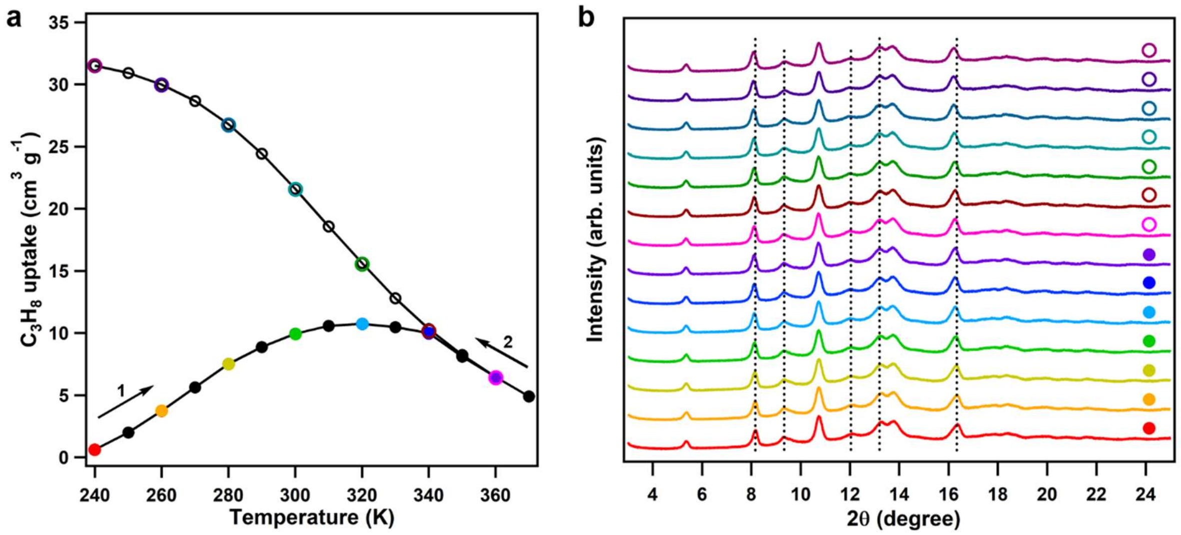

Supplementary Figure 24. (a) C_3H_8-sorption isobar of **FDC–4a** measured in the direction of 240 K → 370 K → 240 K. (b) Coincident *in-situ* isobar/PXRD patterns during C_3H_8 adsorption measured at given temperatures. When the C_3H_8 adsorption isobar was measured in the temperature direction of 240 K → 370 K → 240 K, the adsorption amount continuously increased to 31.5 $cm^3\ g^{-1}$ as the temperature decreased to 240 K. These results suggested that the initial condition of the adsorption is the key to determine the adsorption behavior. At low temperatures, the diffusion was initially impeded and gradually boosted as increasing the temperature, leading to temperature-assisted adsorption. *In-situ* PXRD suggested only very slight structural change at high temperatures in the direction of 370 K → 240 K.

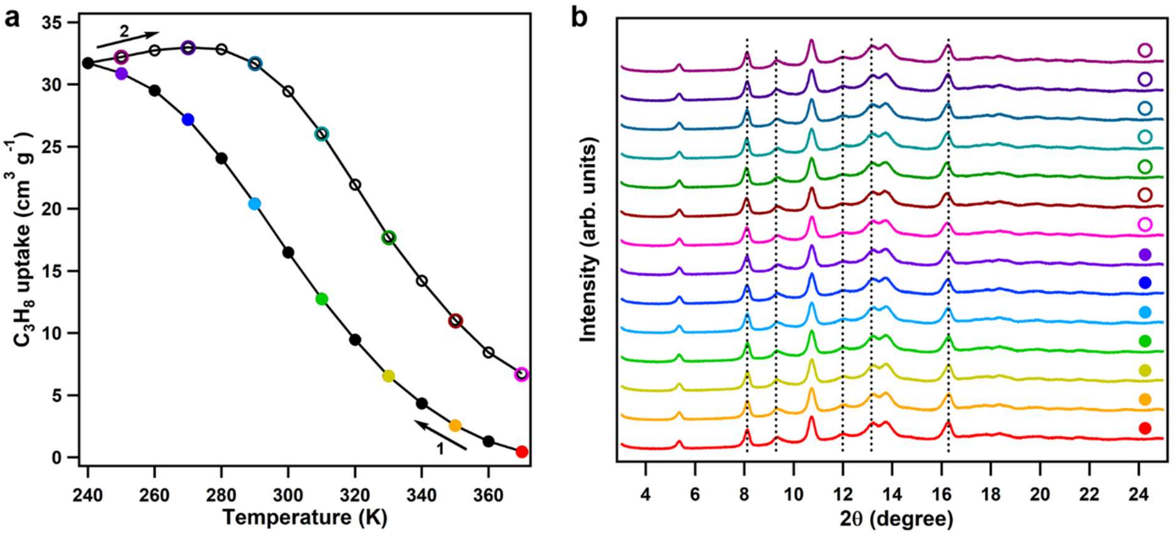

Supplementary Figure 25. (a) C_3H_8-sorption isobar of **FDC–4a** measured in the direction of 370 K → 240 K → 370 K. (b) Coincident *in-situ* isobar/PXRD patterns during C_3H_8 adsorption measured at given temperatures. The adsorptions of 370 K → 240 K and 240 K → 370 K directions showed the same path and obeyed the thermodynamic law, whereas the different uptakes at a certain temperature were presumably because of the kinetic factors. *In-situ* PXRD suggested no structural change during the whole process. The diffusion barrier was expected to be lower if the initial framework was at a high temperature. After adsorbing C_3H_8 the diffusion barrier was further decreased. As a result, **FDC–4a** exhibited ordinary adsorption behavior.

Section 6: Global temperature–diffusion-rate–adsorption amount curves for FDC–4a

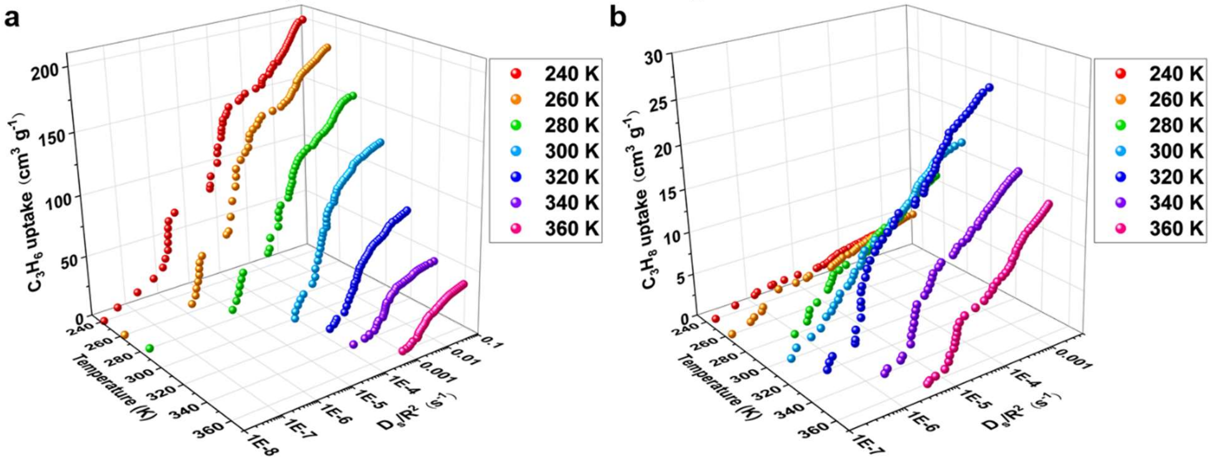

Supplementary Figure 26. Global temperature–diffusion-rate–adsorption amount (T–D_s/R^2–V) landscape for **FDC–4a** adsorbing (a) C_3H_6 and (b) C_3H_8, where R denotes the radius of a PCP particle.

Section 7: VT-PXRD and mechanism for C_3H_6 and C_3H_8 adsorption

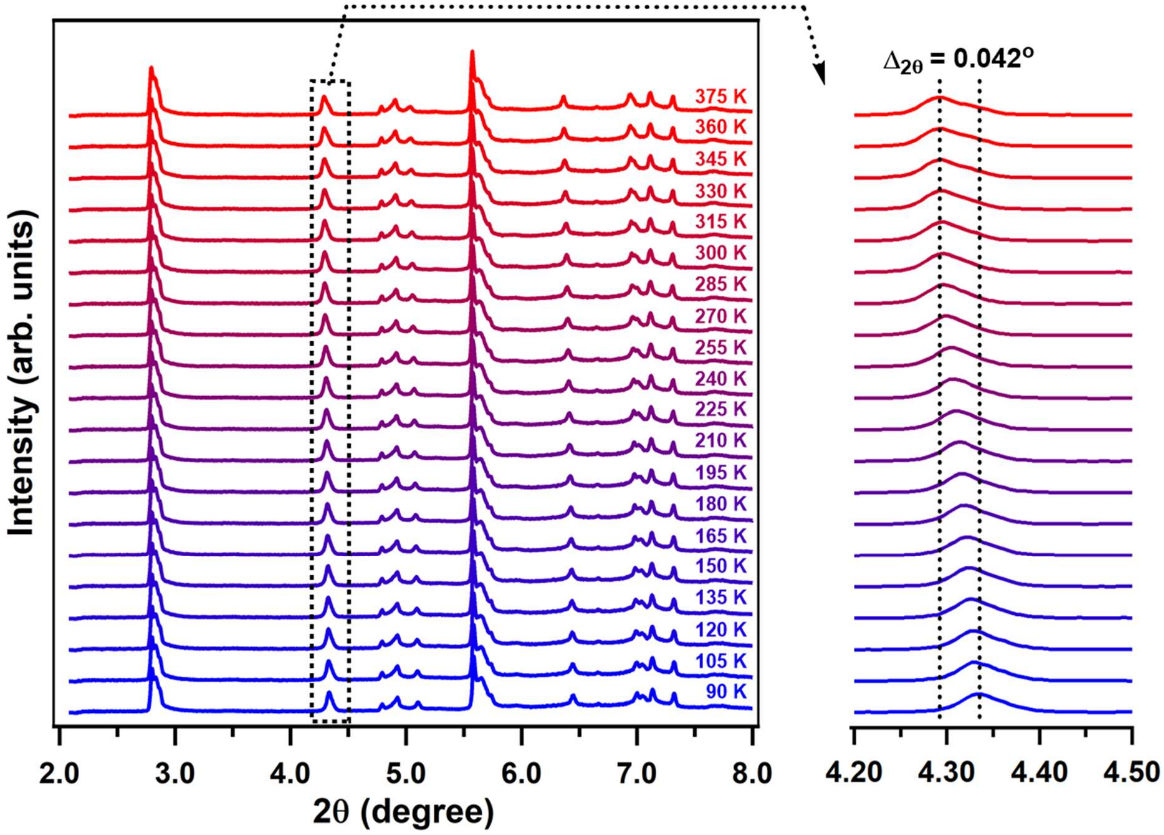

Supplementary Figure 27. Synchrotron VT-PXRD of **FDC–4a** under vacuum conditions in the temperature range from 90 to 375 K.

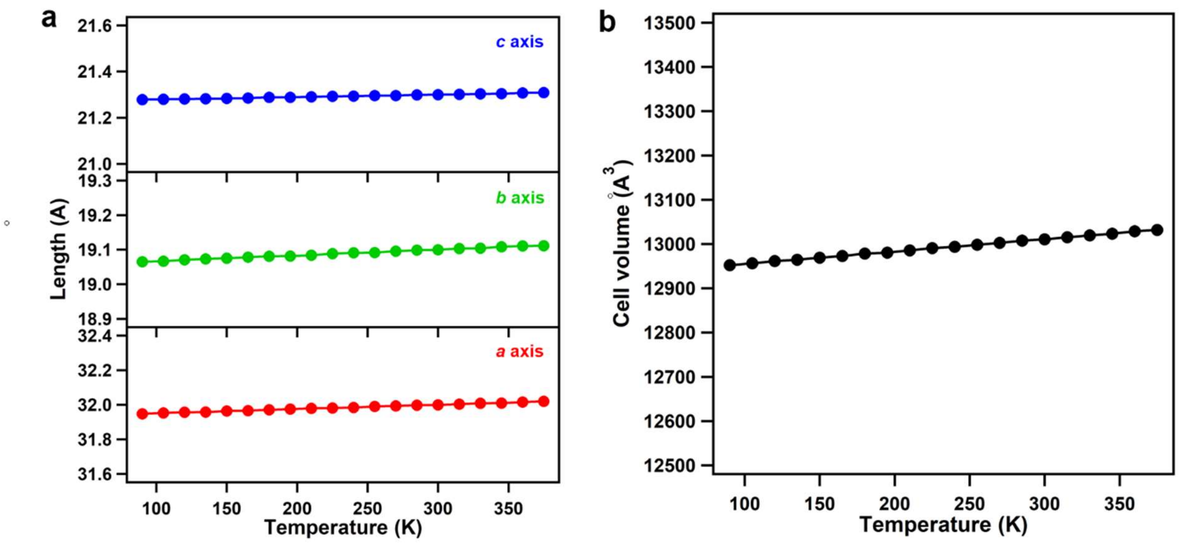

Supplementary Figure 28. (a) Crystal lattice parameters and (b) cell volumes of **FDC–4a** under vacuum at diverse temperatures. As an indication of the relatively “rigid” nature of the frameworks, the unit cell parameters of **FDC–4a** only showed negligible change in various temperatures. The cell volume only increased by 79.5 Å^3 as increasing the temperature from 90 to 375 K. The inconspicuous change of the cell parameters at diverse temperatures indicated the local flip-flop motion with temperatures.

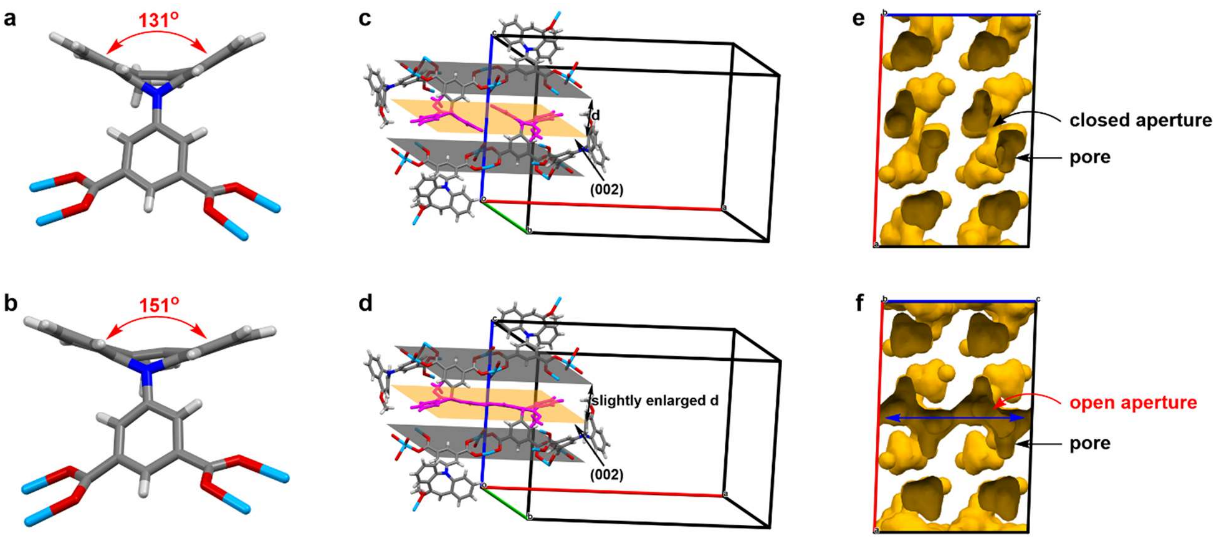

Supplementary Figure 29. Schematic diagram of the effects of the MODBAP flipping on the crystal structure. Configuration of MODBAP-ipa with different phenyl$_{(MODBAP)}$–phenyl$_{(MODBAP)}$ dihedral angles of (a) 131º (termed static) and (b) 151º (or 111º, termed flipping), respectively. Structure related to (002) facet in the unit cell of **FDC–4a** under (c) static and (d) flipping status, respectively. The MODBAP ring in pink color showed the MODBAP moiety on the (002) facet (orange plane). The change of pore aperture of **FDC–4a** under (e) static and (f) flipping status, respectively. The red and blue arrows indicate the pore apertures for diffusion and the diffusion pathways among the pores, respectively. To clearly show the effects of the MODBAP flipping on the crystal structure, we manually changed the phenyl$_{(MODBAP)}$–phenyl$_{(MODBAP)}$ dihedral angles on the MODBAP ring from 131º to 151º (or 111º), while keeping other parts of the crystal structure the same as the initial structure. In these two structures, the structure of the framework constructed with Cu^{2+} paddle-wheels and isophthalates kept the same, whereas the MODBAP moieties showed different phenyl$_{(MODBAP)}$–phenyl$_{(MODBAP)}$ dihedral angles. Thus, the two structures could stand for the non-flipping (static) and flipping modes of **FDC–4a** at low and high temperatures, respectively. The flipping structure revealed a slight expansion of the [002] axis compared to the static structure, in good agreement with the VT-PXRD results that the peaks corresponding to the (002) facet shifted to a lower angle. Since two MODBAP moieties are on the (002) plane, this tiny expansion of [002] distances could be correlated with the extent of thermal flipping of the MODBAP moiety. Notably, the flipping structure showed a remarkable enlargement of pore aperture for the diffusion of C_3H_6 and C_3H_8 compared to the static structure, which indicated that the flipping structure allowed accelerating the diffusion of C_3H_6 and C_3H_8 in response to increasing temperature.

Section 8: Theoretical calculations

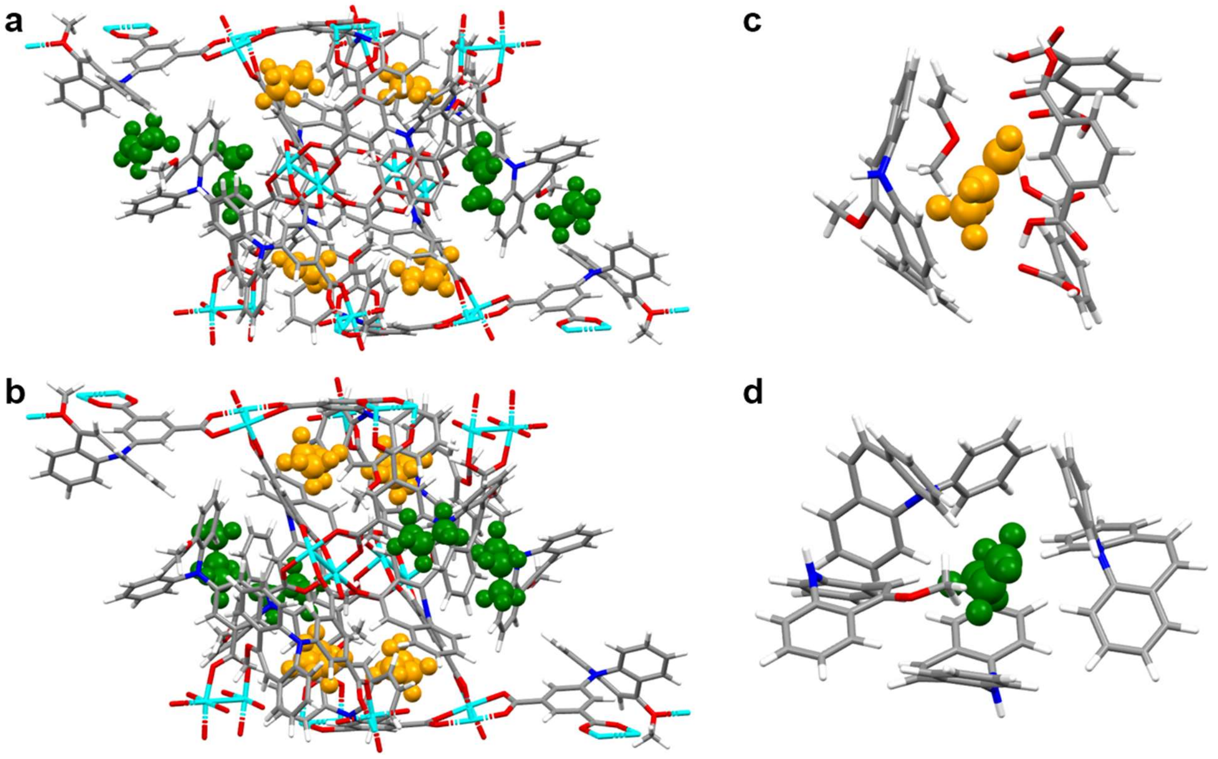

Supplementary Figure 30. Adsorption structures of (a) C_3H_6 and (b) C_3H_8 in **FDC–4a**. The primitive unit cells were used in the calculation. Gas molecules located at sites I and II are represented by orange and green colors, respectively. Cluster models used in RI-MP2 calculations for gas adsorption at site I (c) and site II (d).

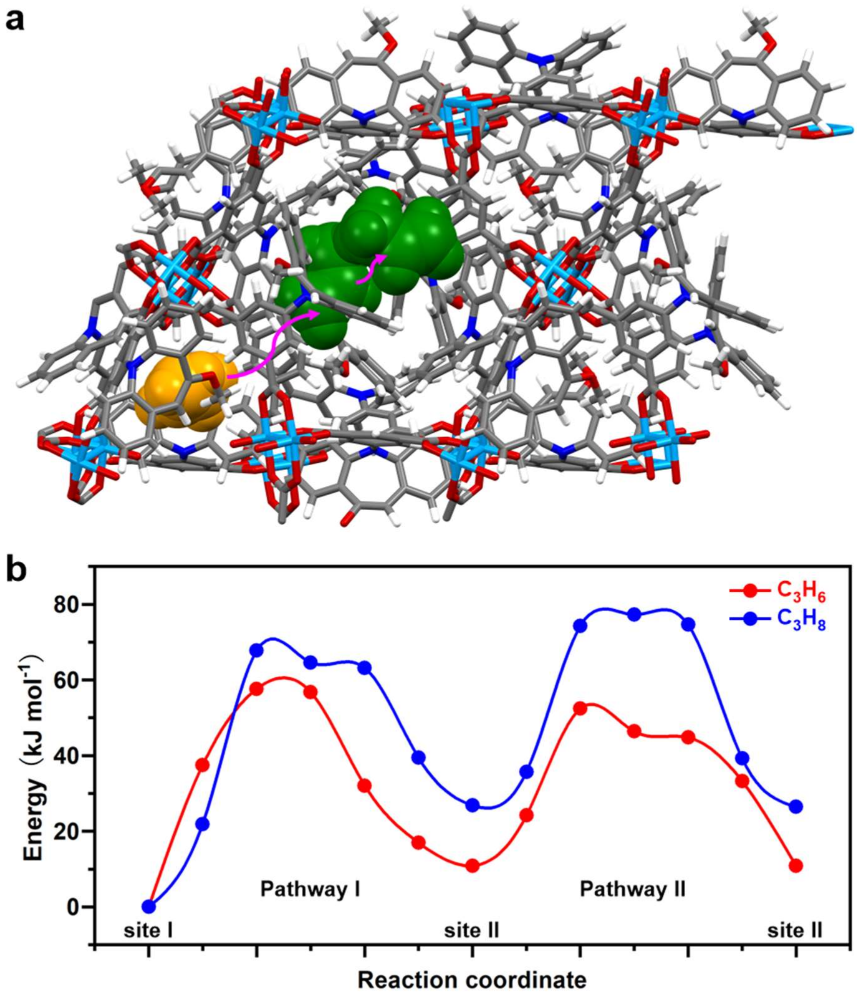

Supplementary Figure 31. (a) Diffusion pathways for C_3H_6 and C_3H_8 in **FDC–4a**; pathway 1 represents the gas transport from site I to site II and pathway II represents the gas transport from site II to another site II. Molecules at the sites I and II are marked as orange and green colors, respectively. (b) Reaction coordinate–energy profile for the diffusion of one C_3H_6 or C_3H_8 molecule through the small aperture in **FDC–4a**.

Section 9: Gas separation experiments for FDC–4a

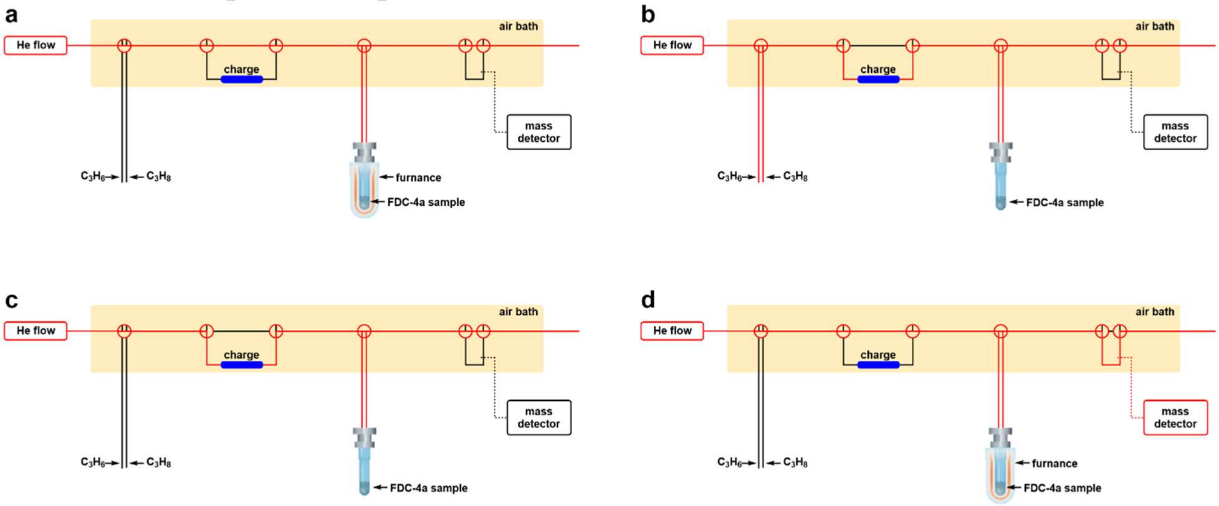

Supplementary Figure 32. Flow path diagram of TPD gas separation experiment for the process (a) No. 1, (b) No. 2, (c) No. 3 (d) No. 4.

Note: Experimental procedure for gas separation with flow diagrams:

No. 1. Activating the sample in the cell under He flow at 393 K for 2 h (a).

No. 2. Mixed gas flowing at 300 K (b).

No. 3. Flowing away remaining gases in the gas line and sample tube with He flush (c).

No. 4. Heating to 393 K to desorb gas from the sample and mass test (d).

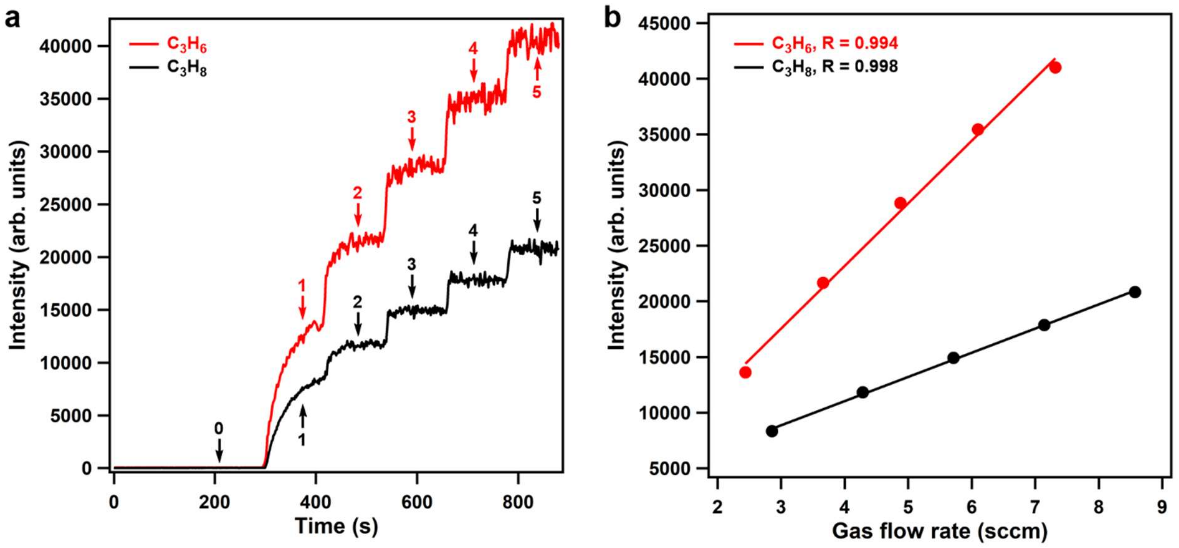

Supplementary Figure 33. Calibration of the C_3H_6/C_3H_8 components in the feed gas. (a) Raw data of the mass detector for five times of gas dosing. (b) Correlation of the electric intensity in the mass detector with the gas flow rate of C_3H_6/C_3H_8 in the feed gas.

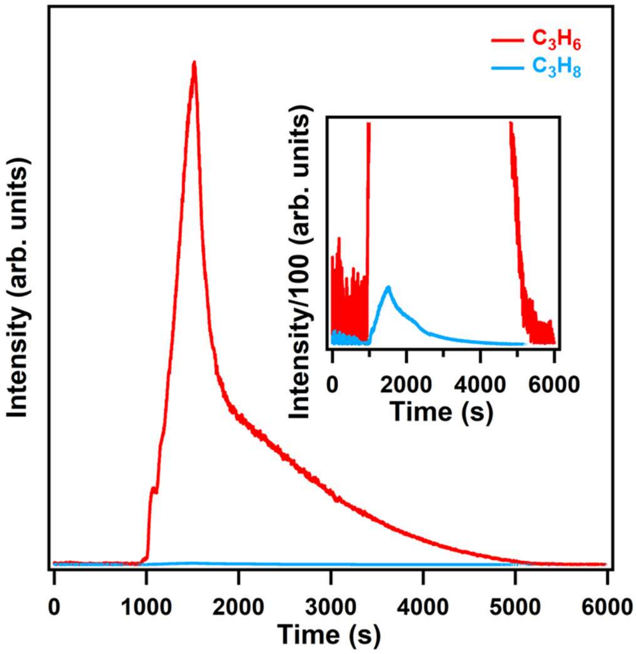

Supplementary Figure 34. TPD spectra of adsorbed gas in **FDC–4a** at 1 h exposure time at 300 K. The feed-gas ratio was C_3H_6:C_3H_8 = 50.0:50.0. The inset is the enlarged Y-axis showing the TPD spectra of C_3H_8.

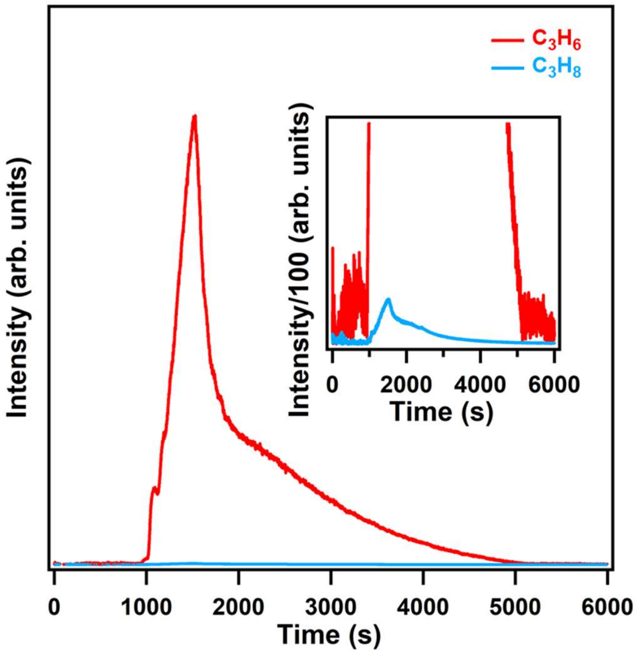

Supplementary Figure 35. TPD spectra of adsorbed gas in **FDC–4a** at 0.5 h exposure time at 300 K. The feed-gas ratio was C_3H_6:C_3H_8 = 50.0:50.0. The inset is the enlarged Y-axis showing the TPD spectra of C_3H_8.

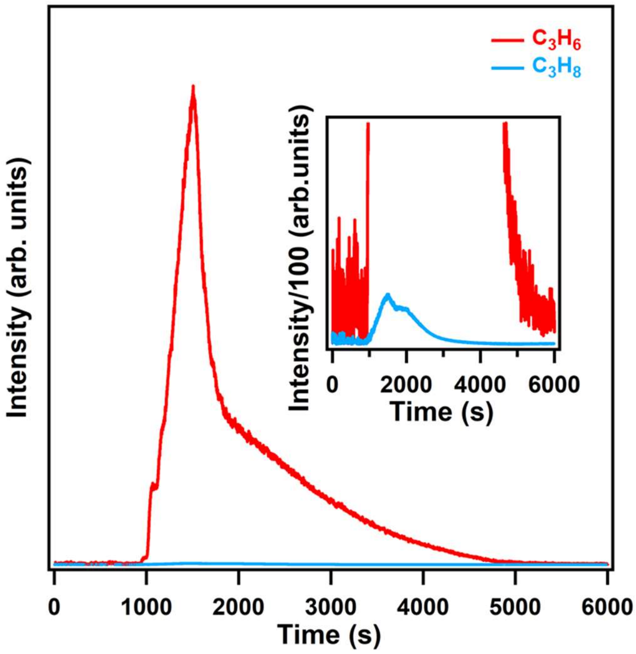

Supplementary Figure 36. TPD spectra of adsorbed gas in **FDC–4a** at 2 h exposure time at 300 K. The feed-gas ratio was C_3H_6:C_3H_8 = 50.0:50.0. The inset is the enlarged Y-axis showing the TPD spectra of C_3H_8.

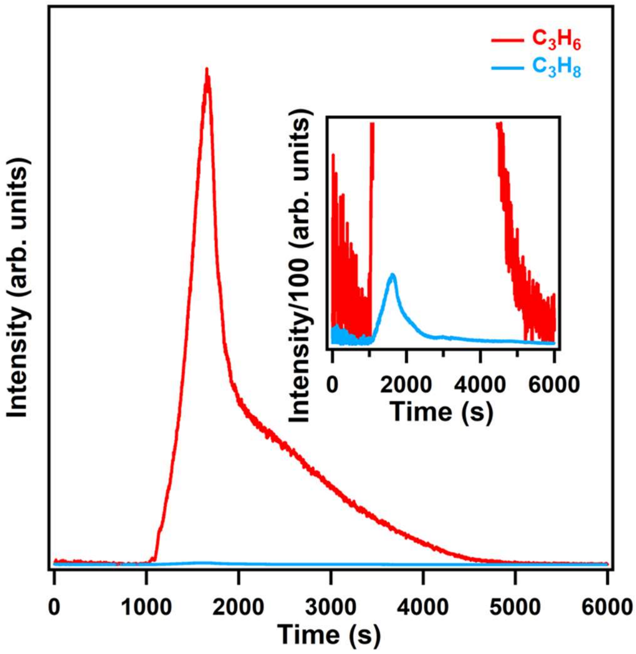

Supplementary Figure 37. TPD spectra of adsorbed gas in **FDC–4a** at 4 h exposure time at 300 K. The feed-gas ratio was C_3H_6:C_3H_8 = 50.0:50.0. The inset is the enlarged Y-axis showing the TPD spectra of C_3H_8.

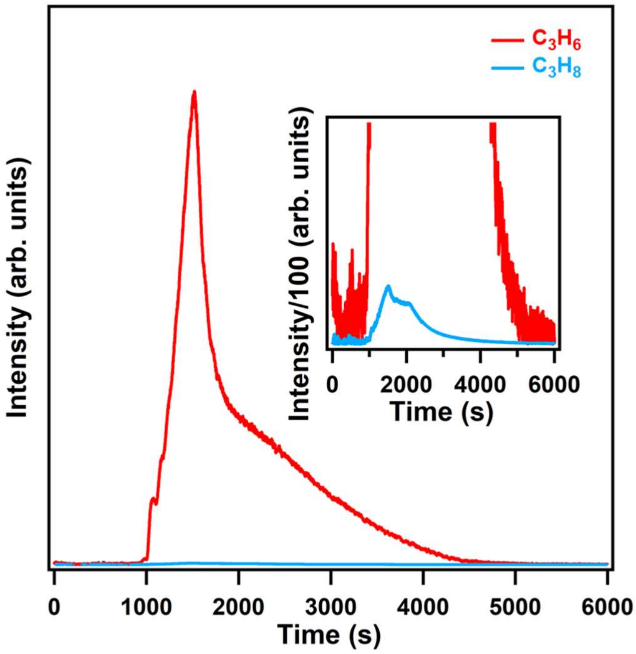

Supplementary Figure 38. TPD spectra of adsorbed gas in **FDC–4a** at 8 h exposure time at 300 K. The feed-gas ratio was C_3H_6:C_3H_8 = 50.0:50.0. The inset is the enlarged Y-axis showing the TPD spectra of C_3H_8.

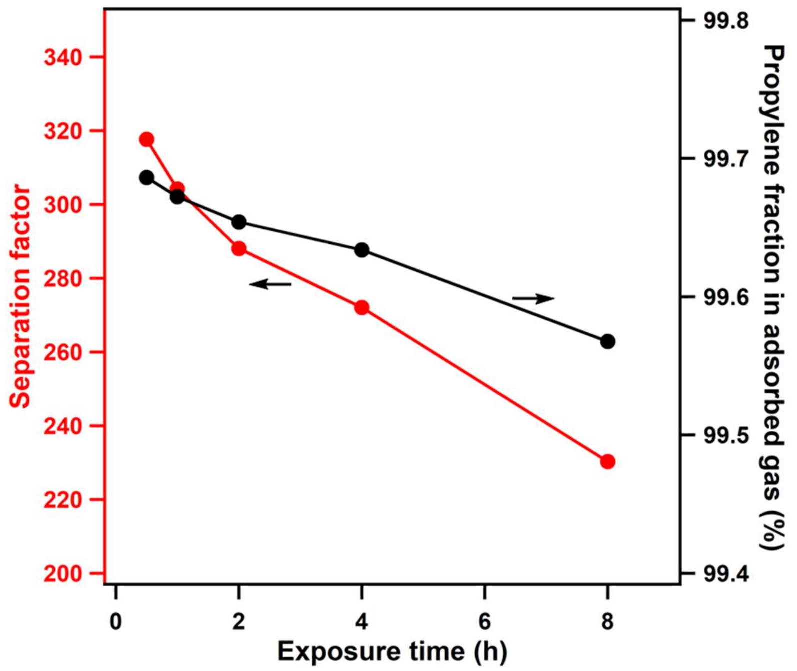

Supplementary Figure 39. Correlation of gas exposure time and separation factor for **FDC–4a**. A value of separation factor larger than unity means that the material preferentially adsorbs C_3H_6. C_3H_6 is preferentially adsorbed on **FDC–4a** over a wide range of exposure time.

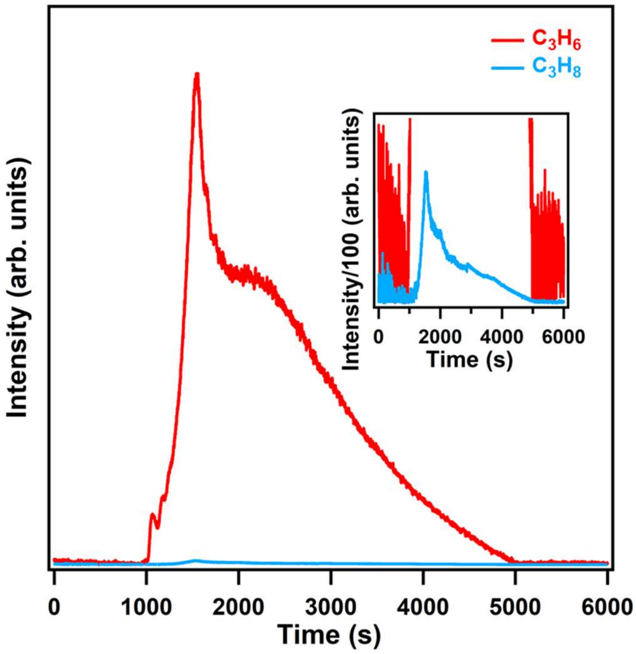

Supplementary Figure 40. TPD spectra of adsorbed gas in **FDC–4a** at 1 h exposure time at 300 K. The feed-gas ratio was C_3H_6:C_3H_8 = 5.0:95.0. The inset is the enlarged Y-axis showing the TPD spectra of C_3H_8.

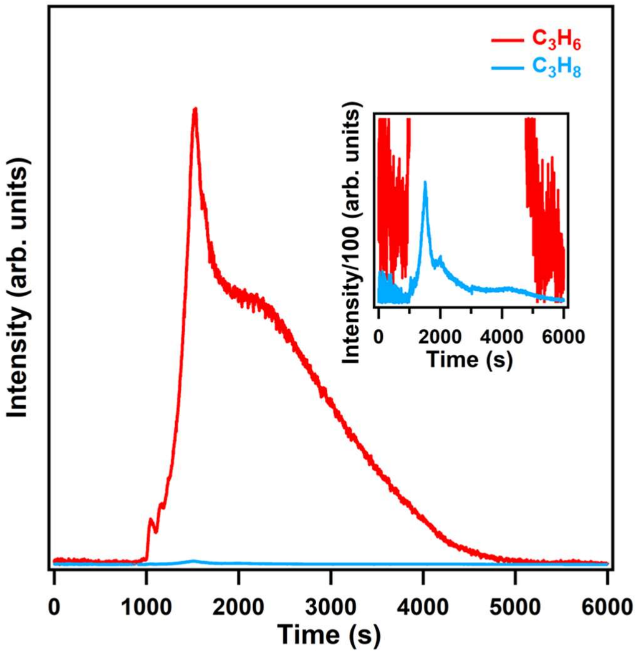

Supplementary Figure 41. TPD spectra of adsorbed gas in **FDC–4a** at 1 h exposure time at 300 K. The feed-gas ratio was C_3H_6:C_3H_8 = 10.0:90.0. The inset is the enlarged Y-axis showing the TPD spectra of C_3H_8.

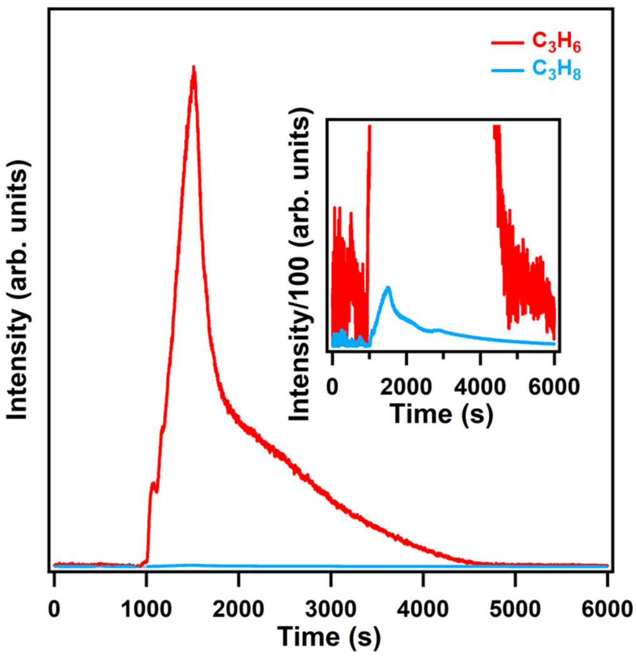

Supplementary Figure 42. TPD spectra of adsorbed gas in **FDC–4a** at 1 h exposure time at 300 K. The feed-gas ratio was C_3H_6:C_3H_8 = 20.0:80.0. The inset is the enlarged Y-axis showing the TPD spectra of C_3H_8.

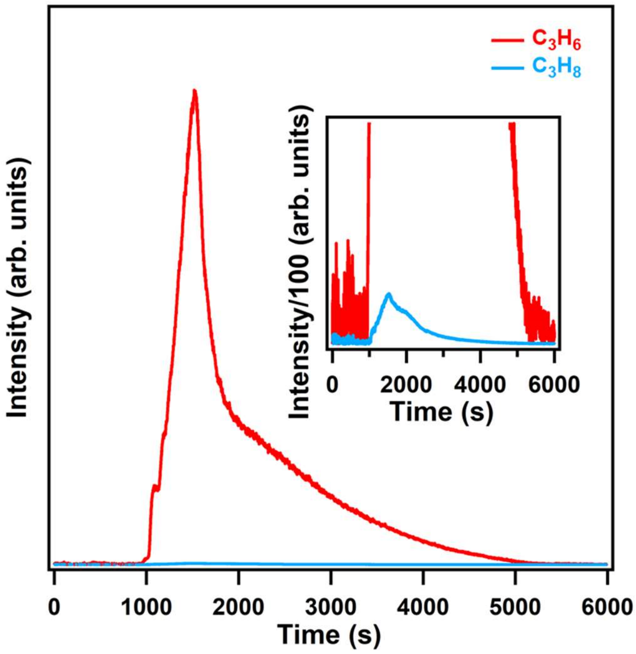

Supplementary Figure 43. TPD spectra of adsorbed gas in **FDC–4a** at 1 h exposure time at 300 K. The feed-gas ratio was C_3H_6:C_3H_8 = 30.0:70.0. The inset is the enlarged Y-axis showing the TPD spectra of C_3H_8.

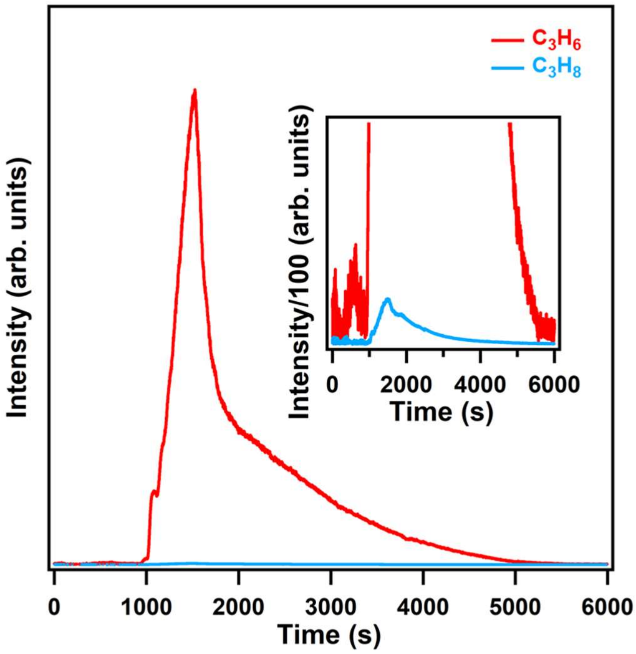

Supplementary Figure 44. TPD spectra of adsorbed gas in **FDC–4a** at 1 h exposure time at 300 K. The feed-gas ratio was C_3H_6:C_3H_8 = 40.0:60.0. The inset is the enlarged Y-axis showing the TPD spectra of C_3H_8.

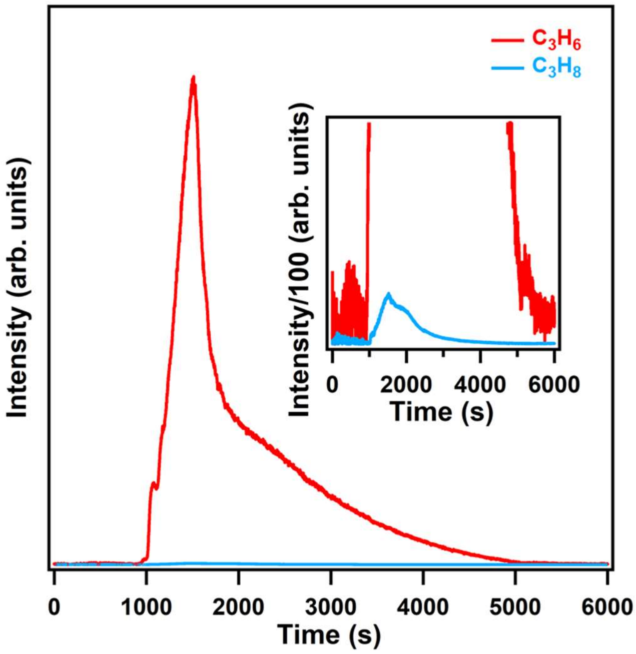

Supplementary Figure 45. TPD spectra of adsorbed gas in **FDC–4a** at 1 h exposure time at 300 K. The feed-gas ratio was C_3H_6:C_3H_8 = 60.0:40.0. The inset is the enlarged Y-axis showing the TPD spectra of C_3H_8.

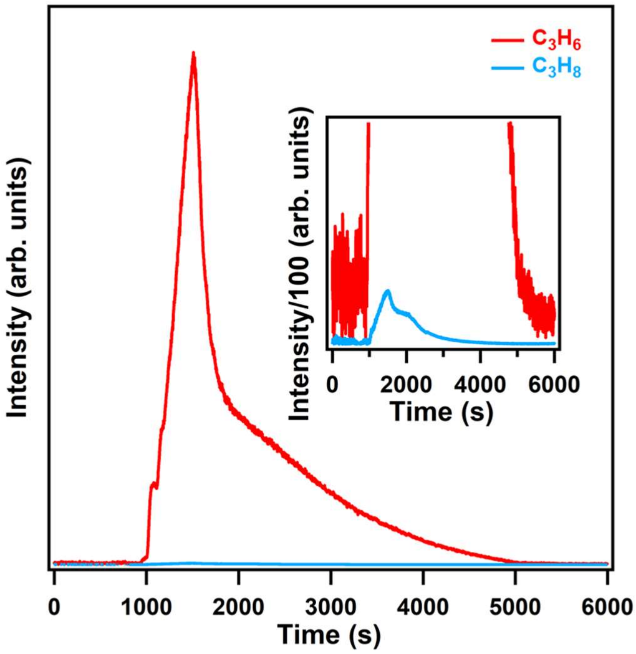

Supplementary Figure 46. TPD spectra of adsorbed gas in **FDC–4a** at 1 h exposure time at 300 K. The feed-gas ratio was C_3H_6:C_3H_8 = 70.0:30.0. The inset is the enlarged Y-axis showing the TPD spectra of C_3H_8.

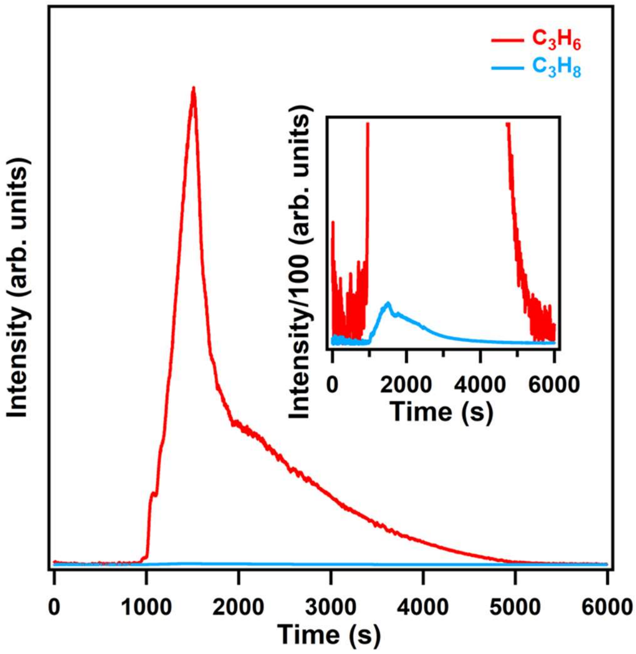

Supplementary Figure 47. TPD spectra of adsorbed gas in **FDC–4a** at 1 h exposure time at 300 K. The feed-gas ratio was C_3H_6:C_3H_8 = 80.0:20.0. The inset is the enlarged Y-axis showing the TPD spectra of C_3H_8.

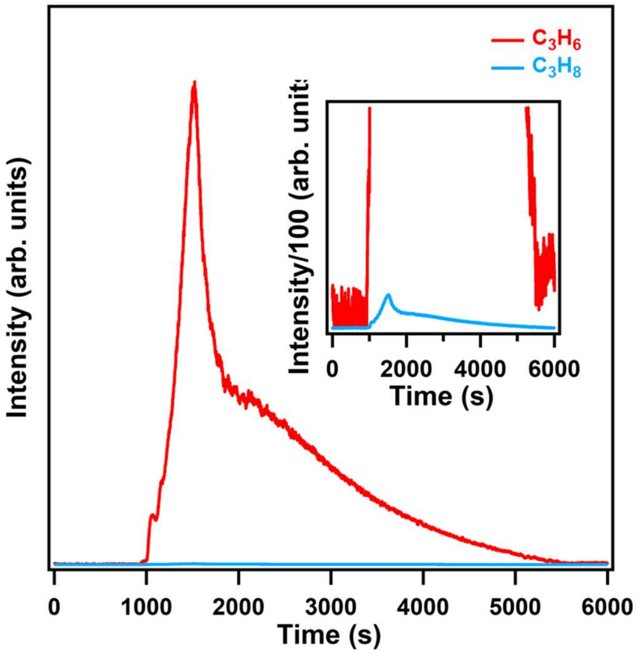

Supplementary Figure 48. TPD spectra of adsorbed gas in **FDC–4a** at 1 h exposure time at 300 K. The feed-gas ratio was C_3H_6:C_3H_8 = 90.0:10.0. The inset is the enlarged Y-axis showing the TPD spectra of C_3H_8.

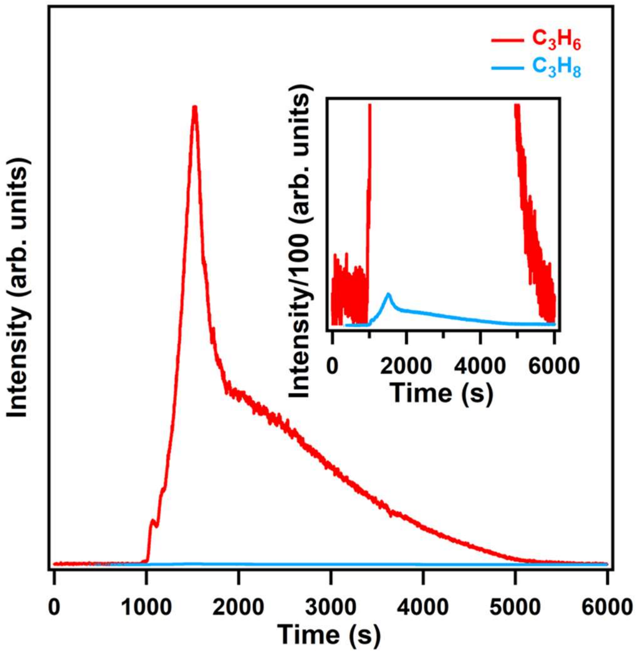

Supplementary Figure 49. TPD spectra of adsorbed gas in **FDC–4a** at 1 h exposure time at 300 K. The feed-gas ratio was C_3H_6:C_3H_8 = 95.0:5.0. The inset is the enlarged Y-axis showing the TPD spectra of C_3H_8.

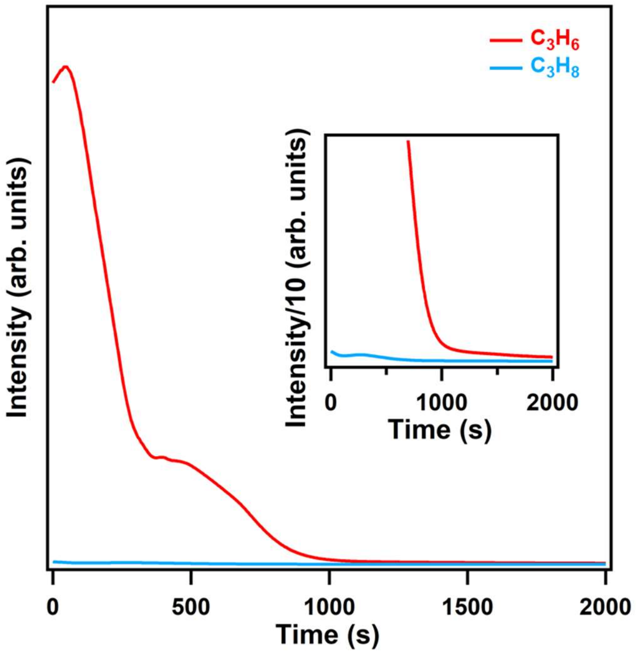

Supplementary Figure 50. The desorption curves of C_3H_6 and C_3H_8 in the breakthrough experiment with an equimolar C_3H_6/C_3H_8 mixture as the feed gas, the total flow rate of 4 mL min^{-1}, and the desorption temperature of 393 K. The dead time has been deducted. The inset is the enlarged Y-axis showing the desorption curve of C_3H_8.

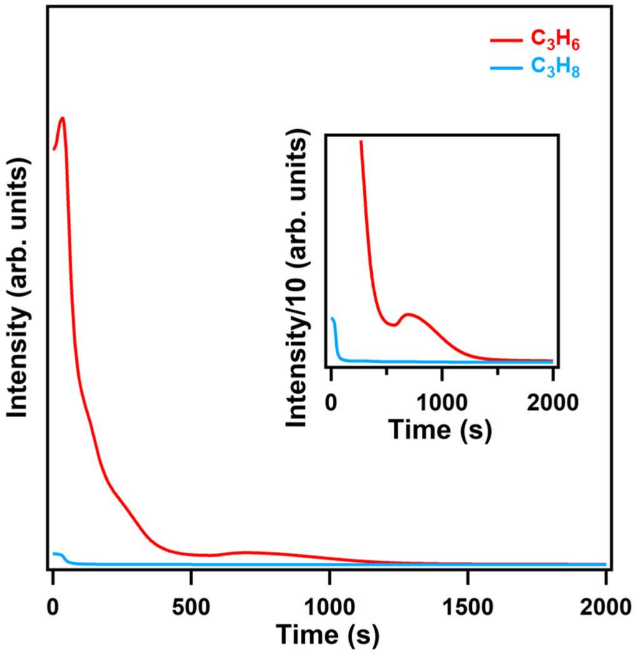

Supplementary Figure 51. The desorption curves of C_3H_6 and C_3H_8 in the breakthrough experiment with an equimolar C_3H_6/C_3H_8 mixture as the feed gas, the total flow rate of 10 mL min^{-1}, and the desorption temperature of 393 K. The dead time has been deducted. The inset is the enlarged Y-axis showing the desorption curve of C_3H_8.

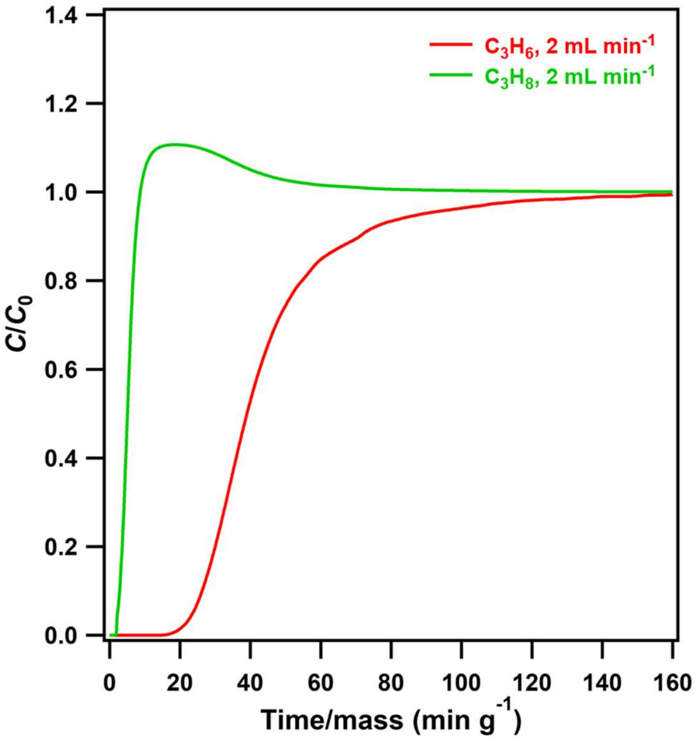

Supplementary Figure 52. The breakthrough curve of a 10:90 C_3H_6/C_3H_8 mixture (total flow rate of 4.0 mL min^{-1}) on **FDC–4a** at 300 K. *C* and C_0 are the concentrations of each gas at the outlet and inlet, respectively.

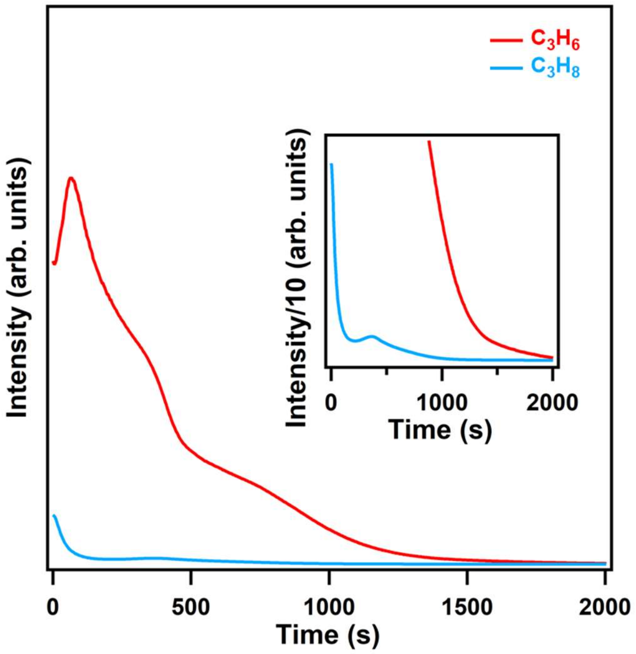

Supplementary Figure 53. The desorption curves of C_3H_6 and C_3H_8 in the breakthrough experiment with a 10:90 C_3H_6/C_3H_8 mixture as the feed gas, the total flow rate of 4 mL min^{-1}, and the desorption temperature of 393 K. The dead time has been deducted. The inset is the enlarged Y-axis showing the desorption curve of C_3H_8.

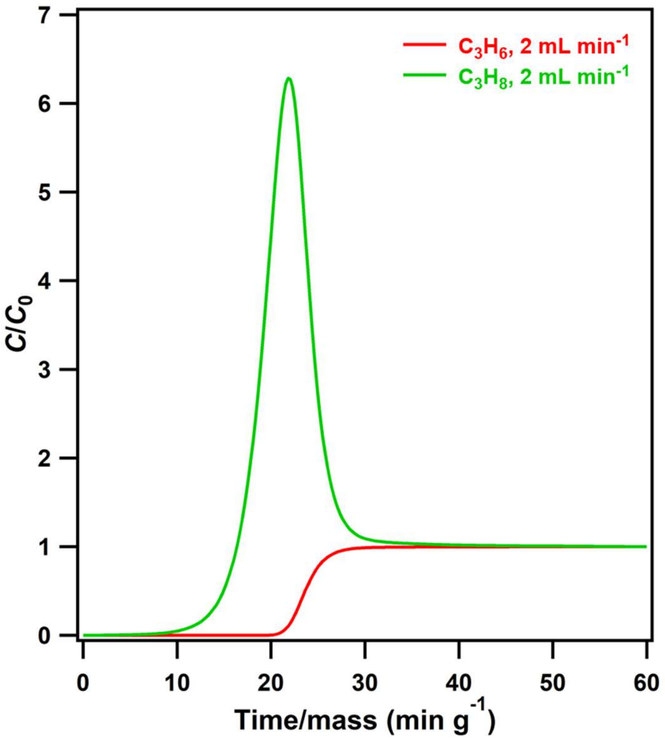

Supplementary Figure 54. The breakthrough curve of a 90:10 C_3H_6/C_3H_8 mixture (total flow rate of 4.0 mL min^{-1}) on **FDC–4a** at 300 K. C and C_0 are the concentrations of each gas at the outlet and inlet, respectively.

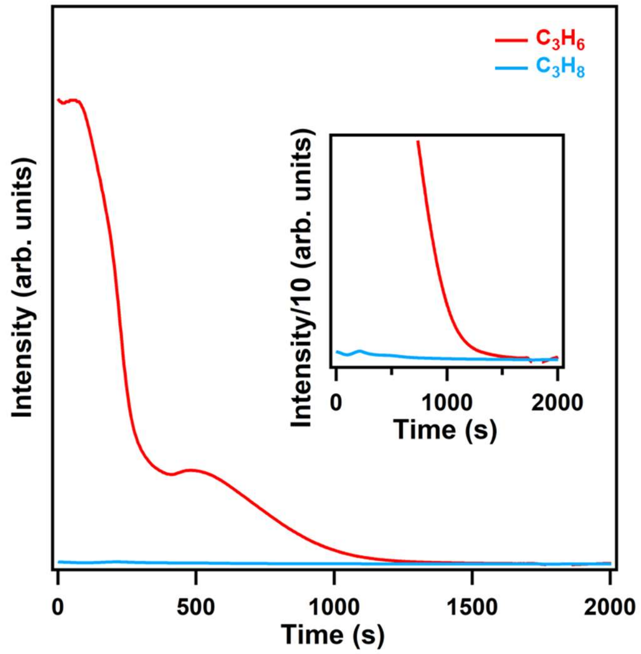

Supplementary Figure 55. The desorption curves of C_3H_6 and C_3H_8 in the breakthrough experiment with a 90:10 C_3H_6/C_3H_8 mixture as the feed gas, the total flow rate of 4 mL min^{-1}, and the desorption temperature of 393 K. The dead time has been deducted. The inset is the enlarged Y-axis showing the desorption curve of C_3H_8.

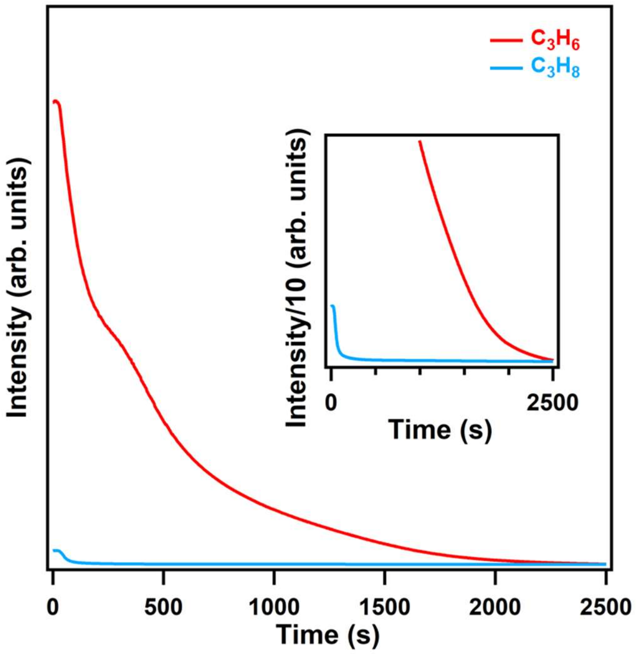

Supplementary Figure 56. The desorption curves of C_3H_6 and C_3H_8 in the breakthrough experiment with an equimolar C_3H_6/C_3H_8 mixture as the feed gas, the total flow rate of 4 mL min^{-1}, and the desorption temperature of 300 K. The dead time has been deducted. The inset is the enlarged Y-axis showing the desorption curve of C_3H_8.

Supplementary Tables

Section 1: Structures and physicochemical properties of C_3H_6 and C_3H_8

Supplementary Table 1. Structures and physicochemical properties of C_3H_6 and C_3H_8[S30].

	C_3H_6	C_3H_8
	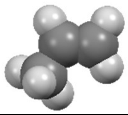	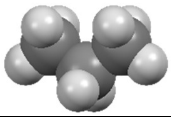
kinetic diameter (Å)	4.678	4.3–5.118
molecular size (Å^3)	6.44 × 4.65 × 4.16	6.61 × 4.52 × 4.02
standard boiling point (K)	225.46	231.02
critical temperature (K)	364.90	369.83
critical pressure (bar)	46.00	42.48
critical volume (m^3 kg^{-1})	184.60	200.00
polarizability (×10^{25} cm^3)	62.6	4.3–5.118

Section 2: Crystallographic data for as-synthesized and activated FDCs

Supplementary Table 2. Crystallographic data and structural refinement summary for as-synthesized FDC–4.

	as-synthesized **FDC–4**
crystal system	monoclinic
space group	I2 (#5)
empirical formula	$C_{25}H_{23}N_5O_{20}Cu_3$
a (Å)	21.6812(9)
b (Å)	18.6598(8)
c (Å)	23.7776(11)
α (°)	90
β (°)	91.563(4)
γ (°)	90
V (Å^3)	9616.0(7)
Z, d_{calcd} (g cm^{-3})	4, 0.624
diffractometer	four-circle diffractometer
μ (cm^{-1})	0.690
radiation type	Mo $K\alpha$
radiation wavelength (Å)	0.71073
F(000)	1820
goodness of fit	0.971
temperature (K)	293(2)
number of reflections collected/unique	25612/13914
R_{int}	0.0603
R_1 ($I > 2.00\sigma(I)$)[a]	0.0798
wR_2 ($I > 2.00\sigma(I)$)[b]	0.2059
CCDC deposition number	2236283

[a]$R_1 = \Sigma||F_o|-|F_c||/\Sigma|F_o|$, [b]$wR_2 = [\Sigma w|F_o^2-F_c^2|^2/\Sigma w(F_o^2)^2]^{1/2}$

Supplementary Table 3. Crystallographic data and structural refinement summary for activated FDC–4a.

	activated **FDC–4a**
crystal system	monoclinic
space group	C2/c (#15)
empirical formula	$C_{2.029}H_{1.324}N_{0.088}O_{0.441}Cu_{0.088}$
a (Å)	32.000(6)v
b (Å)	19.100(4)
c (Å)	21.300(4)
α (°)	90
β (°)	92.00(3)
γ (°)	90
V (Å^3)	13011(5)
Z, d_{calcd} (g cm^{-3})	272, 1.699
diffractometer	Bruker APEX-II CCD
μ (cm^{-1})	0.000
radiation type	electron
radiation wavelength (Å)	0.02508
F(000)	2110
goodness of fit	1.661
temperature (K)	293(2)
number of reflections collected/unique	9140/4771
R_{int}	0.2905
R_1 ($I > 2.00\sigma(I)$)[a]	0.2331
wR_2 ($I > 2.00\sigma(I)$)[b]	0.5141
CCDC deposition number	2236284

[a]$R_1 = \Sigma||F_o|-|F_c||/\Sigma|F_o|$, [b]$wR_2 = [\Sigma w|F_o^2-F_c^2|^2/\Sigma w(F_o^2)^2]^{1/2}$

Section 3: Comparison of C_3H_6/C_3H_8 adsorption performances of the benchmark materials

Supplementary Table 4. Summary of the C_3H_6 and C_3H_8 uptakes and Q_{st} in various porous materials.

Material	T (K)	P (bar)	C_3H_6 uptake (cm^3 g^{-1})	C_3H_8 uptake (cm^3 g^{-1})	Q_{st} of C_3H_6 (kJ mol^{-1})	Ref
UTSA-400	298	1	92.1[a]	2.5[a]	60.5	5
Y-abtc	298	1	43.3	2.5	50	6
Ni-NP	298	1	79.9	47.7	57	8
Co_2(dobdc)	298	1	164.2	120.6	--	9
Fe_2(m-dobdc)	298	1	163.8	132.8	73.0	10
MAF-23-O	298	1	30.2	22.4	54	11
ZJU-75a	296	1	104.3	73.5	65.9	12
MFM-520	298	1	52.2	45.5	48.5	13
HIAM-301	298	1	70.8	<6.72	27	14
PCP-IPA	298	1	55.0	54.2	37.73	15
KAUST-7	298	1	29.5	0.9	57.4	16
NTU-85-WNT	298	1	20.9	0.13	49.9	17
Co-gallate	298	1	37.5	4.2	41	18
JNU-3a	298	1	58.6	48.0	29.3	19
**FDC–4a**	**300**	**1**	**121.6**	**19.6**	**35.0**	**This work**

[a]The unit for uptake is cm^3 cm^{-3}.

Section 4: Calculated energies for C_3H_6 and C_3H_8 adsorptions and diffusions in FDC–4a

Supplementary Table 5. Calculated binding energy (kJ mol^{-1}) for C_3H_6 and C_3H_8 adsorption in FDC–4a.

	$BE^{PBE\text{-}D3}$		$BE^{SCS\text{-}MP2:PBE\text{-}D3}$	
	site I	site II	site I	site II
**C_3H_6**	−67.2	−55.6	−47.3	−34.9
**C_3H_8**	−73.6	−46.7	−48.7	−23.2

Supplementary References

1. Sheldrick, G. M. A short history of SHELX. *Acta Crystallogr. Sect. A: Found. Crystallogr.* **64**, 112–122 (2008).

2. Nishibori, E. *et al.* The large Debye–Scherrer camera installed at SPring-8 BL02B2 for charge density studies. *Nucl. Instrum. Methods Phys. Res. A* **467/468**, 1045–1048 (2001).

3. Takata, M. *et al.* High resolution Debye-Scherrer camera installed at SPring-8. *Advances in X-Ray Analysis* **45**, 377–384 (2002).

4. Xie, Y. *et al.* Tuning the topology of three-dimensional covalent organic frameworks via steric control: from pts to unprecedented ljh. *J. Am. Chem. Soc.* **143**, 7279–7284 (2021).

5. Kärger, J., Ruthven, D. M. & Theodorou, D. N. *Diffusion in nanoporous materials* (WILEY-VCH Verlag GmbH & Co. kGaA, 2012).

6. Kresse, G. & Furthmüller, J. Efficiency of ab-initio total energy calculations for metals and semiconductors using a plane-wave basis set. *Comput. Mater. Sci.* **6**, 15–50 (1996).

7. Kresse, G. & Furthmüller, J. Efficient iterative schemes for *ab initio* total-energy calculations using a plane-wave basis set. *Phys. Rev. B* **54**, 11169–11186 (1996).

8. Frenkel, D. & Smit, B. Understanding molecular simulation. Computational Science Series. Academic Press, San Diego Adcock SA, McCammon JA (2002).

9. Dubbeldam, D., Calero, S., Ellis, D. E. & Snurr, R. Q. RASPA: molecular simulation software for adsorption and diffusion in flexible nanoporous materials. *Mol. Simul.* **42**, 81–101 (2016).

10. Rappé, A. K., Casewit, C. J., Colwell, K., Goddard III, W. A. & Skiff, W. M. UFF, a full periodic table force field for molecular mechanics and molecular dynamics simulations. *J. Am. Chem. Soc.* **114**, 10024–10035 (1992).

11. Martin, M. G. & Siepmann, J. I. Transferable potentials for phase equilibria. 1. United-atom description of n-alkanes. *J. Phys. Chem. B* **102**, 2569–2577 (1998).

12. Wick, C. D., Martin, M. G. & Siepmann, J. I. Transferable potentials for phase equilibria. 4. United-atom description of linear and branched alkenes and of alkylbenzenes. *J. Phys. Chem. B* **104**, 8008–8016 (2000).

13. Perdew, J. P., Burke, K. & Ernzerhof, M. Generalized gradient approximation made simple. *Phys. Rev. Lett.* **77**, 3865–3868 (1996).

14. Grimme, S., Antony, J., Ehrlich, J. & Krieg, J. A consistent and accurate *ab initio* parametrization of density functional dispersion correction (DFT-D) for the 94 elements H-Pu. *J. Chem. Phys.* **132**, 154104-1–154104-19 (2010).

15. Blöchl, P. E. Projector augmented-wave method. *Phys. Rev. B* **50**, 17953–17979 (1994).

16. Kresse, G. & Joubert, D. From ultrasoft pseudopotentials to the projector augmented-wave method. *Phys. Rev. B* **59**, 1758–1775 (1999).

17. Dudarev, S. L., Botton, G. A., Savrasov, S. Y., Humphreys, C. J. & Sutton, A. P. Electron-energy-loss spectra and the structural stability of nickel oxide: an LSDA+U study. *Phys. Rev. B* **57**, 1505–1509 (1998).

18. Wang, L., Maxisch, T. & Ceder, G. Oxidation energies of transition metal oxides within the GGA+U framework. *Phys. Rev. B* **73**, 195107-1–195107-6 (2006).

19. Dunlap, B. I., Connolly, J. & Sabin, J. R. On some approximations in applications of Xα theory. *J. Chem. Phys.* **71**, 3396–3402 (1979).

20. Smith, D. G. A. *et al.* Psi4 1.4: open-source software for high-throughput quantum chemistry. *J. Chem. Phys.* **152**, 184108 (2020).

21. Dunning Jr., T. H. Gaussian basis sets for use in correlated molecular calculations. I. The atoms boron through neon and hydrogen. *J. Chem. Phys.* **90**, 1007–1023 (1989).

22. Kendall, R. A., Dunning Jr., T. H. & Harrison, R. J. Electron affinities of the first−row atoms revisited. Systematic basis sets and wave functions. *J. Chem. Phys.* **96**, 6796–6806 (1992).

23. Weigend, F., Köhn, A. & Hättig, C. Efficient use of the correlation consistent basis sets in resolution of the identity MP2 calculations. *J. Chem. Phys.* **116**, 3175–3183 (2002).

24. Hättig, C. Optimization of auxiliary basis sets for RI-MP2 and RI-CC2 calculations: Core–valence and quintuple-ζ basis sets for H to Ar and QZVPP basis sets for Li to Kr. *Phys. Chem. Chem. Phys.* **7**, 59–66 (2005).

25. Boys, S. F. & Bernardi, F. The calculation of small molecular interactions by the differences of separate total energies. Some procedures with reduced errors. *Mol. Phys.* **19**, 553–566 (1970).

26. Henkelman, G., Uberuaga, B. P. & Jónsson, H. A climbing image nudged elastic band method for finding saddle points and minimum energy paths. *J. Chem. Phys.* **113**, 9901–9904 (2000).

27. Frisch, M. J. *et al. Gaussian 09*, revision D.01; Gaussian, Inc: Wallingford, CT, 2013.

28. Gu, C. *et al.* Design and control of gas diffusion process in a nanoporous soft crystal. *Science* **363**, 387–391 (2019).

29. Su, Y., Otake, K.-i., Zheng, J.-J., Horike, S., Kitagawa, S. & Gu, C. Separating water isotopologues using diffusion-regulatory porous materials. *Nature* **611**, 289–294 (2022).

30. Li, J.-R., Kuppler, R. J. & Zhou, H.-C. Selective gas adsorption and separation in metal-organic frameworks. *Chem. Soc. Rev.* **38**, 1477–1504 (2009).
