# Supplementary material for: Diffusion-rate sieving of propylene and propane mixtures in a cooperatively dynamic porous crystal
Source: Nat Commun. 2024 Apr 4;15:2898. doi: 10.1038/s41467-024-47268-7 (PMC10995200; doi:10.1038/s41467-024-47268-7)
Supplement: Supplementary file 2 — Peer Review Filef [file 41467_2024_47268_MOESM2_ESM.pdf]

nature portfolio

Peer Review File

Diffusion-rate sieving of propylene/propane in a cooperatively dynamic porous crystal

Open Access This file is licensed under a Creative Commons Attribution 4.0 International License, which permits use, sharing, adaptation, distribution and reproduction in any medium or format, as long as you give appropriate credit to the original author(s) and the source, provide a link to the Creative Commons license, and indicate if changes were made. In the cases where the authors are anonymous, such as is the case for the reports of anonymous peer reviewers, author attribution should be to 'Anonymous Referee' followed by a clear attribution to the source work. The images or other third party material in this file are included in the article's Creative Commons license, unless indicated otherwise in a credit line to the material. If material is not included in the article's Creative Commons license and your intended use is not permitted by statutory regulation or exceeds the permitted use, you will need to obtain permission directly from the copyright holder. To view a copy of this license, visit http://creativecommons.org/licenses/by/4.0/.

Editorial Note: This manuscript has been previously reviewed at another journal that is not operating a transparent peer review scheme. This document only contains reviewer comments and rebuttal letters for versions considered at Nature Communications.

Reviewers' Comments:

Reviewer #1:
Remarks to the Author:
I have examined the manuscript in light to the responses given to reviewers 1,3, and 4. A good amount of revisions have been conducted and most comments addressed.

The proposed breakthrough measurements could not be taken at this time due to various reasons explained (mostly because the high quality sample cannot be re produced). While we appreciate the honesty of the authors in the difficulty of reproducing these materials in the original quality and in reproducing the original breakthrough data, and all the breakthrough data has been removed, this is a little worrying as only reproduceable results should be reported. Please take extra care in the future study.

A few reviewers have raised the point that IAST is not suitable for this system and I suggest that this should be removed as well to avoid confusions.

A final point, the concept of 'omnidynamics' clearly is not favoured by most reviewers. This I feel is unnecessary to support this study and should be removed too.

Reviewer #2:
Remarks to the Author:
Although this material is not a novel material since the synthesis strategy has already been reported (Science, 2019, 363, 387–391/Nature, 2022, 611, 289-294), it additionally demonstrates the synergy effect of global and local dynamics. In addition, the separation performance of the material shows relatively good performance among the reported materials. The paper has undergone numerous revisions compared to the previous version, so if the following changes are made, it is potentially suitable for publication in Nature Communications.

1. There are still limitations in considering 270-280 K as the gate-opening temperature. In Figure S28, the gate opening phenomenon appears as the temperature increases from below 270 K. Figure S28 is VT-PXRD under vacuum conditions, so if the increased gate opening temperature is due to affinity with C_3H_6, the peak of PXRD should show changes in coincident in-situ isobar/PXRD patterns during C_3H_6 adsorption. In Figure S23, the peaks are changed slightly as the temperature increases, but it is unclear by limiting it to the temperature of 270-280 K. I think that either additional evidence should be provided that it is the gate opening temperature or the relevant expression should be modified.

2. While the term 'omnidynamic' is introduced in this paper for the first time, the phenomenon itself is not novel. The sentence originally attributing it to the combination of thermodynamic and kinetic factors has been revised to explain it as a combination of global and local dynamics. However, this concept is not new; it has been previously reported in flexible MOFs. It is not advisable to use such an expression to creat the impression that a new phenomenon has been discovered. I recommend not using the term 'omnidynamics'.

3. The explanation of Figure 1 in the main text seems to be partially incorrect and needs to be confirmed.
- (Page 2, line 20) "sizes/kinetics (Fig. 1a, right top)"
- (Page 3, line 16) "unattainable thermodynamic equilibrium (Fig. 1a, middle)"
- (Page 4, line 8) "C3H8 is not able to induce the deformation of the framework (Fig. 1a, right)."

4. The revision is needed.
- (Supplementary Figure S24(a)) "C3H8 uptake --> C3H6 uptake"

Reviewer #5:
Remarks to the Author:
In this work, Gu et al. present a nice case of flexible MOF for the separation of propane and propylene. The detailed gas adsorption analysis and the comprehensive characterization is impressive. It shows certain unique findings compared to other work in this field. And the authors have made great efforts to address the reviewers' comments from the previous round. Personally, I fell the current results could merit publication in Nature Communications, although the manuscript falls short on completeness. However, the following concerns exist, which may not be easily addressed based on the responses from the authors.

Essentially no structural information regarding gas-framework interaction and detailed framework transformation was obtained through in-situ PXRD.

It has been recognized by more than one reviewers as well as the authors that the IAST selectivity calculation of the un-equilibrium isotherms in this work would lead to unreliable numbers. Thus there is no point to show Figure 3b and put only the current material in the so called ideal molecular sieves region.

Breakthrough data. This a crucial piece of information. The claimed high separation performance must be verified by breakthrough measurements. It is otherwise meaningless. The authors argued that single crystals could not be reproduced due to the change of climate and the obtained powder performed poorly. This is not convincing at all. In practical condition, there is no doubt powder will be used through large-scale synthesis.

"The essences of these approaches included the control of thermodynamics and sizes/kinetics, with the kinetically controlled process being an extreme scenario of size exclusive sieving." This is not correct. Size exclusive sieving is an extreme scenario of kinetically controlled process, not the opposite.

Response to Referee #1

I have examined the manuscript in light to the responses given to reviewers 1,3, and 4. A good amount of revisions have been conducted and most comments addressed.

The proposed breakthrough measurements could not be taken at this time due to various reasons explained (mostly because the high quality sample cannot be reproduced). While we appreciate the honesty of the authors in the difficulty of reproducing these materials in the original quality and in reproducing the original breakthrough data, and all the breakthrough data has been removed, this is a little worrying as only reproduceable results should be reported. Please take extra care in the future study.

===

We appreciate the comments from the reviewer endorsing the revision of our work. Regarding the breakthrough results mentioned by the reviewer, we would like to apologize again for this unexpected thing. This was the first time our group moved to another city, and we never expected the environment to play such an important role in reproducing the samples. With the reviewer's suggestion in mind, in the future study, we will pay more attention to reproducing the samples and will endeavor to complete all the required data before the initial submission. We have taken significant consideration of all the comments and suggestions from the reviewer and revised our manuscript accordingly. We believe that the revised manuscript has clarified all the requests and comments from the reviewer.

===

A few reviewers have raised the point that IAST is not suitable for this system and I suggest that this should be removed as well to avoid confusions.

===

We thank the reviewer for this constructive comment. Accordingly, we have removed all the "IAST selectivity" parts throughout the manuscript and the supplementary materials.

===

A final point, the concept of 'omnidynamics' clearly is not favoured by most reviewers. This I feel is unnecessary to support this study and should be removed too.

===

According to the suggestion from the reviewer, we have removed all the terms "omnidynamics".

According to the suggestion from the reviewer, we have revised our manuscript critically. We believe the revision has clarified all the issues raised by the reviewer. We sincerely hope the reviewer can understand our positive situation regarding the revision and be satisfied with the revisions we performed. We appreciate the reviewer for the above constructive comments that improve the manuscript.

===

Response to Referee #2

Although this material is not a novel material since the synthesis strategy has already been reported (Science, 2019, 363, 387–391/Nature, 2022, 611, 289-294), it additionally demonstrates the synergy effect of global and local dynamics. In addition, the separation performance of the material shows relatively good performance among the reported materials. The paper has undergone numerous revisions compared to the previous version, so if the following changes are made, it is potentially suitable for publication in Nature Communications.

==

We appreciate the comments from the reviewer endorsing the revision of our work. Regarding the concern raised by the reviewer, we have taken significant consideration of all the comments and suggestions from the reviewer and revised our manuscript accordingly. We believe that the revised manuscript has clarified all the requests and comments from the reviewer.

==

1. There are still limitations in considering 270-280 K as the gate-opening temperature. In Figure S28, the gate opening phenomenon appears as the temperature increases from below 270 K. Figure S28 is VT-PXRD under vacuum conditions, so if the increased gate opening temperature is due to affinity with C3H6, the peak of PXRD should show changes in coincident in-situ isobar/PXRD patterns during C3H6 adsorption. In Figure S23, the peaks are changed slightly as the temperature increases, but it is unclear by limiting it to the temperature of 270-280 K. I think that either additional evidence should be provided that it is the gate opening temperature or the relevant expression should be modified.

==

We appreciate the reviewer for this thought-provoking comment. We agree with the reviewer that the PXRD patterns changed not significantly in the 270-280 K. Furthermore, we found the gate-opening temperature was not that important for discussing the gas-adsorption behavior in this study. Therefore, according to the reviewer's suggestion, we removed the following sentence in the manuscript to avoid misleading:

"*The C_3H_6 adsorption isobar revealed an apparent decrease in uptake amount in the temperature of 270 to 280 K, which was attributed to the temperature threshold of gate opening (T_{go}).*".

==

2. While the term 'omnidynamic' is introduced in this paper for the first time, the phenomenon itself is not novel. The sentence originally attributing it to the combination of thermodynamic and kinetic factors has been revised to explain it as a combination of global and local dynamics. However, this concept is not new; it has been previously reported in flexible MOFs. It is not advisable to use such an expression to creat the impression that a new phenomenon has been discovered. I recommend not using the term 'omnidynamics'.

==

According to the suggestion from the reviewer, we have removed all the terms “omnidynamics”.

==

3. The explanation of Figure 1 in the main text seems to be partially incorrect and needs to be confirmed.

- (Page 2, line 20) “sizes/kinetics (Fig. 1a, right top)”

- (Page 3, line 16) “unattainable thermodynamic equilibrium (Fig. 1a, middle)”

- (Page 4, line 8) “C3H8 is not able to induce the deformation of the framework (Fig. 1a, right).”

==

According to the suggestion from the reviewer, we have revised the above explanations to the following sentences for better clarification:

“*The essence of these approaches included the control of thermodynamics (Fig. 1a, left top) and kinetics (Fig. 1a, right top), with the kinetically controlled process being an extreme scenario of size-exclusive sieving.*”;

“*However, these PCPs encounter sluggish kinetics in the separation process because the thermodynamic equilibrium is difficult to achieve (Fig. 1a, middle).*”;

“*C_3H_6 exhibits high gas-framework interaction energy and causes the PCP to globally deform accompanied by a gate-opening adsorption behavior, whereas the diffusion of C_3H_8 is regulated by the local motion of the ligand because the weak C_3H_8-PCP interaction renders the global structural change of PCP impossible (Fig. 1a, right).*”.

==

4. The revision is needed.

- (Supplementary Figure S24(a)) “C3H8 uptake --> C3H6 uptake”

==

We apologize for the typo. We have revised it in the new Supplementary Figure S23a.

According to the suggestion from the reviewer, we have revised our manuscript critically. We believe the revision has clarified all the issues raised by the reviewer. We sincerely hope the reviewer can understand our positive situation regarding the revision and be satisfied with the revisions we performed. We appreciate the reviewer for the above constructive comments that improve the manuscript.

==

Response to Referee #5

In this work, Gu et al. present a nice case of flexible MOF for the separation of propane and propylene. The detailed gas adsorption analysis and the comprehensive characterization is impressive. It shows certain unique findings compared to other work in this field. And the authors have made great efforts to address the reviewers' comments from the previous round. Personally, I fell the current results could merit publication in Nature Communications, although the manuscript falls short on completeness. However, the following concerns exist, which may not be easily addressed based on the responses from the authors.

==

We appreciate the comments from the reviewer endorsing the quality of our work. Regarding the concern raised by the reviewer, we have taken significant consideration of all the comments and suggestions from the reviewer and revised our manuscript accordingly. We believe that the revised manuscript has clarified all the requests and comments from the reviewer.

==

Essentially no structural information regarding gas-framework interaction and detailed framework transformation was obtained through in-situ PXRD.

==

We appreciate the reviewer for this thought-provoking comment. Indeed, the *in-situ* PXRD results showed some structural changes in the C_3H_6 adsorption, but these changes were gradual, indicating that the structural change was not very large. This was different from many PCPs which showed large structural changes and stepped gate-opening behaviors in adsorption processes. Such slight structural changes in our PCP system were difficult to observe from *in-situ* PXRD results. On the other hand, the C_3H_6-PCP interaction was weak (Q_{st} = 35.0 kJ mol^{-1}), rendering it difficult to investigate the structural information regarding gas-framework interaction through *in-situ* PXRD. Additionally, our collaborator was not able to measure the cRED under the C_3H_6 environment, making the determination of the adsorbed phase impossible. Therefore, currently, we are not able to provide more structural information regarding the gas-framework interaction and detailed framework transformation. However, with the reviewer's suggestion in mind, we have started to seek more collaborators and crystallographers to solve

this issue. We sincerely hope that the reviewer can understand the difficulty we encountered and recognize our positive attitude towards the structural confirmation.

==

It has been recognized by more than one reviewers as well as the authors that the IAST selectivity calculation of the un-equilibrium isotherms in this work would lead to unreliable numbers. Thus there is no point to show Figure 3b and put only the current material in the so called ideal molecular sieves region.

==

We thank the reviewer for this constructive comment. Accordingly, we have removed all the "IAST selectivity" parts throughout the manuscript and the supplementary materials.

==

Breakthrough data. This a crucial piece of information. The claimed high separation performance must be verified by breakthrough measurements. It is otherwise meaningless. The authors argued that single crystals could not be reproduced due to the change of climate and the obtained powder performed poorly. This is not convincing at all. In practical condition, there is no doubt powder will be used through large-scale synthesis.

==

Regarding the breakthrough results mentioned by the reviewer, we would like to apologize again for this unexpected thing. Indeed, not only the climate but all the conditions, even the vials, chemicals, and separation facilities, changed after our lab moved. This was the first time our group moved to another city, and we never expected the environment to play such an important role in reproducing the samples. We are annoyed by this. Currently, the precise and reliable data were the TPD results, which were fully performed in our previous lab. From the results, we can clearly see that our PCP selectively adsorbed C_3H_6 and excluded C_3H_8. Of course, we fully agree with the reviewer that the large-scale synthesis in practical conditions is powder, and we should improve the powder quality. With the reviewer's suggestion in mind, we will pay more attention to reproducing the samples and will endeavor to complete all the required data before the initial submission. We would like to reproduce the samples and report the breakthrough results in future manuscripts. We still believe that we found interesting chemistry in this manuscript, and it would be a little pity if the manuscript was rejected only

because the missing of breakthrough results. We sincerely hope that the reviewer can understand the difficulty we encountered and recognize our positive attitude in the preciseness of the manuscript.

==

"The essences of these approaches included the control of thermodynamics and sizes/kinetics, with the kinetically controlled process being an extreme scenario of size exclusive sieving." This is not correct. Size exclusive sieving is an extreme scenario of kinetically controlled process, not the opposite.

==

We are sorry for confusing the reviewer because of a careless typo. According to the suggestion from the reviewer, we have modified this sentence in the revised manuscript:

"*The essence of these approaches included the control of thermodynamics (Fig. 1a, left top) and kinetics (Fig. 1a, right top), with the size-exclusive sieving being an extreme scenario of the kinetically controlled process.*".

According to the suggestion from the reviewer, we have revised our manuscript critically. We believe the revision has clarified all the issues raised by the reviewer. We sincerely hope the reviewer can understand our positive situation regarding the revision and be satisfied with the revisions we performed. We appreciate the reviewer for the above constructive comments that improve the manuscript.

==

Reviewers' Comments:

Reviewer #1:
Remarks to the Author:
The authors have addressed my concerns and I am happy to recommend for publication.

Reviewer #2:
Remarks to the Author:
In this revised manuscript, the authors addressed all comments given by the reviewers. The current manuscript is now acceptable to Nature Communications.

Reviewer #5:
Remarks to the Author:
The auhtors have adequetely addressed the concerns and I would like to reccommend its publication in Nat. Commun.
